# Computational Insights on the Mechanism of the Chemiluminescence Reaction of New Group of Chemiluminogens—10-Methyl-9-thiophenoxycarbonylacridinium Cations

**DOI:** 10.3390/ijms21124417

**Published:** 2020-06-21

**Authors:** Milena Pieńkos, Beata Zadykowicz

**Affiliations:** Faculty of Chemistry, University of Gdańsk, Wita Stwosza 63, 80-308 Gdańsk, Poland; milena.pienkos@phdstud.ug.edu.pl

**Keywords:** mechanism of chemiluminescence, DFT and TD DFT calculations, acridinium derivatives, chemiluminogens

## Abstract

Immunodiagnostics, in which one of the promising procedures is the chemiluminescent labelling, is essential to facilitate the detection of infections in a human organism. One of the standards commonly used in luminometric assays is luminol, which characterized by low quantum yield in aqueous environments. Acridinium esters have better characteristics in this topic. Therefore, the search for new derivatives, especially those characterized by the higher quantum yield of chemiluminescence, is one of the aims of the research undertaken. Using the proposed mechanism of chemiluminescence, we examined the effect of replacing a single atom within a center of reaction on the efficient transformation of substrates into electronically excited products. The density functional theory (DFT) and time dependent (TD) DFT calculated thermodynamic and kinetic data concerning the chemiluminescence and competitive dark pathways suggests that some of the scrutinized derivatives have better characteristics than the chemiluminogens used so far. Synthesis of these candidates for efficient chemiluminogens, followed by studies of their chemiluminescent properties, and ultimately in chemiluminescent labelling, are further steps to confirm their potential applicability in immunodiagnostics.

## 1. Introduction

Chemical demonstrations are very popular and allow the promotion of science, especially among children and youth. Spectacular chemical experiments, especially those that are unexpected, explosive and colorful, attract the full auditoria. One of the most amazing chemical experiments successfully presented at chemical demonstrations is chemiluminescence (CL) [1], in which by mixing several substances a luminous product is formed [2]. In fact, chemiluminescence is a phenomenon of the emission of light as a result of a chemical reaction [3]. In this reaction, a product is formed in an electronically excited state [4,5]. When an electronically excited electron returns to a ground state, an energy is released in a form of light (as a photon) [5]. In addition to cognitive features, chemiluminescence has also found many utilitarian applications in clinical [6,7], pharmaceutical [8], chemical and biochemical analysis [9], and also in environmental [10] and food analysis [11].

**Scheme 1:**
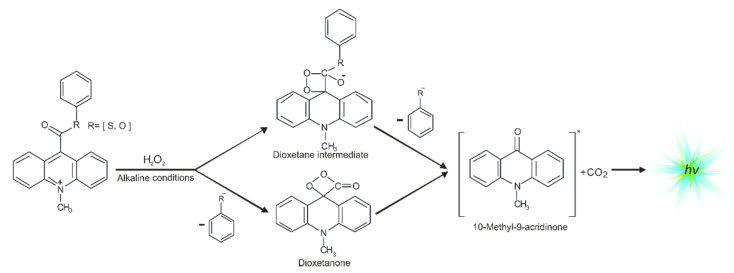
Proposed chemiluminescence mechanism of acridinium derivatives.

One of the most efficient chemiluminogens are compounds based on quaternary acridine derivatives substituted in the central ring at the endocyclic nitrogen atom and the opposing carbon atom (in position 9), especially 10-methyl-9-phenoxycarbonylacridnium cations (acridinium esters) [12,13,14,15,16]. These acridinium salts exhibit chemiluminogenic features as a result of reaction with oxidants in alkaline environments [12,17]. Acridinium esters are used in clinical [18,19], chemical [5,20], or environmental analysis [10,21] as fragments of chemiluminescent labels in commercial tests [22] in which the determination of concentration of macromolecules, e.g., α-fetoproteins [22], β-*d*-galactosidase [23,24], glucose-6-phosphate dehydrogenase [22], TSH [24,25], FT4 [22], or anti-HIV antibodies [26,27], are very precise (it is possible to determine the concentrations of macromolecules at the femtomole level [6,11,26,28,29,30,31,32,33]). Considering the utilitarian role of acridinium derivatives, it is important to understand how electronically excited products are formed. The mechanism of chemiluminescence of acridinium esters was suggested by McCapra in the late 1980s [1,34], and then was studied using quantum chemistry methods (first at the semiempirical [35] and then at the density functional theory level [17]). The chemiluminescence reaction of acridinium salts (Scheme 1) begins with the attack of oxidant, e.g., OOH^−^ on carbon atom in position 9 of acridine moiety, followed by the reaction of the addition product with OH^−^ [12,17], either the cyclization of the anion to cyclic intermediate [17], and the elimination of phenyl carbonate anion, leading to the energy-rich 10-methyl-9-acridinone, or the elimination of the adequate phenoxy anion and formed the cyclic intermediate—four-membered highly strained dioxetane structure [12,36,37,38,39,40,41], and the subsequent elimination of carbon dioxide leading to the energy-rich 10-methyl-9-acridinone. The theoretically predicted thermodynamic data indicated that these two pathways are possible for most compounds [12,17]. The relaxation of electronically excited product is accompanied by the emission of light, i.e., chemiluminescence [4,42].

The acridinium derivatives are one of the most important groups of compounds used in the chemiluminometric analysis. Their biggest advantage is the relatively high quantum yield of chemiluminescence—up to 7% [34]. It is also important that acridinium esters are easy to synthesize and oxidize in an aqueous alkaline environment [12,43]. However, the search for new derivatives, especially those characterized by the higher quantum yield of chemiluminescence, is one of the aims of the research undertaken. Taking into account previous investigations on the mechanism of chemiluminescence of acridinium derivatives, it is known that the type of the leaving group is affected by the efficiency of the formation of the electronically excited products [12,17,35]. Therefore, the studies on new chemiluminogens, in which a phenoxy group was replaced by a thiophenoxy group, were initiated.

In the current work, we computationally investigate a series of 10-methyl-9-thiophenoxycarbonylacridinium derivatives, comparing their susceptibility to oxidation in an alkaline media. We suggest the mechanism of the chemiluminescence reaction of new group of compounds based on acridine moiety, considering both, light—leading to the energy-rich product and dark—leading to competitive products, reaction pathways. Given the possibilities of modern quantum chemistry methods, we focused our study on the ground state reaction profiles and formation of the electronically excited 10-methyl-9-acridinone. By understanding the chemiluminescence mechanism, new potential chemiluminogens with specificity and susceptibility could be rationally designed.

## 2. Results and Discussion

### 2.1. Selection of Investigated Models

**Scheme 2:**
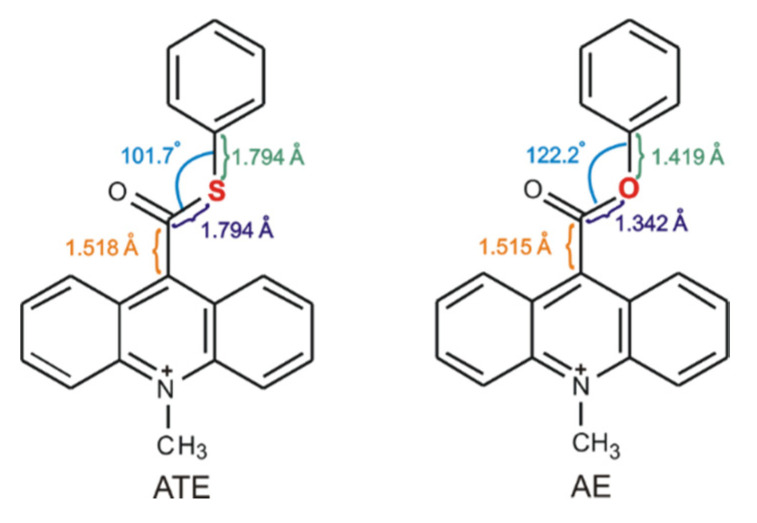
Comparison of structural properties within the leaving group of acridinium ester (AE) and investigated acridinium thioester (ATE).

Our previous investigations [12,13,17] on the mechanism of the chemiluminescence of acridinium derivatives indicate that the type of the leaving group is affected the efficiency of the formation of the electronically excited products. For this reason, we began to analyze the molecules, in which elimination of the leaving group could be much easier than in acridinium esters and thus transformation into cyclic intermediate, leading to the electronically excited product, much efficient. We decided to replace only one atom in the leaving group (Scheme 2) and check how such a small difference in the molecular structure of acridinium esters will affect the efficiency of the chemiluminescence reaction. The selection of the replacing atom was based on the electronegativity of chemical elements (χ in the Pauling scale is equal to 3.44 for oxygen atom and 2.58 for sulphur atom [44]) and structural properties within the leaving group of the investigated compounds. The bond lengths in the thiocarboxyl group are much longer than in the carboxyl group (the C–S bond is equal to 1.8 Å, the C–O bond is ca. 1.3 Å) and the C–C bond between thiocarboxyl group and acridine moiety in acridinium thioester (Scheme 2, ATE) increases slightly compared to the same bond in acridinium ester (Scheme 2, AE). The reduction of electronegativity and increasing the bond lengths within the leaving group suggest easier elimination of the leaving group and thus more efficient transformation to the electronically excited product. Other aspects to consider are feasible for the synthesis of new chemiluminogens after the oxygen-by-sulphur exchange and their stability of using in applications. Based on the literature [1,5,12,13,17], it can be predicted that the synthesis of acridinium thioesters will be similar to those of acridinium esters and will proceed in three steps: the conversion of acridine-9-carboxylic acid into 9-chlorocarbonyl acridine hydrochloride, the esterification of 9-chlorocarbonyl acridine to acridine thioester and the methylation of the endocyclic nitrogen atom to obtain acridinium salt. Considering the physical or/and chemical properties of the sulphur atom, it can be assumed that also acridinium thioesters will be stable in aqueous and organic environments. The chemiluminogens containing sulphur atom are known [13], so we expect the possibility of application of acridinium thioesters as a chemiluminogenic fragment in chemiluminescent labels.

**Scheme 3:**
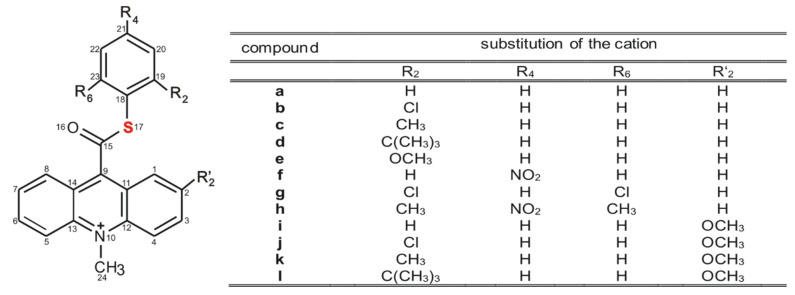
Structure of investigated acridinium thioesters (ATEs) with the numbering of atoms indicated.

The models selected to investigation are 10-methyl-9-(thiophenoxycarbonyl)acridinium cations substituted with various alkyl groups or an electronegative methoxy group, nitro group or halogen in the thiophenyl fragment (Scheme 3, a–h) and also in the acridine moiety (Scheme 3, i–l). These compounds were selected for investigations since the electron-attracting features of the substituents in the thiophenyl fragment may substantially affect their affinity for nucleophiles and susceptibility to oxidation (chemiluminescence efficiency), and consequently their applicability.

### 2.2. Orbitals

**Figure 1:**
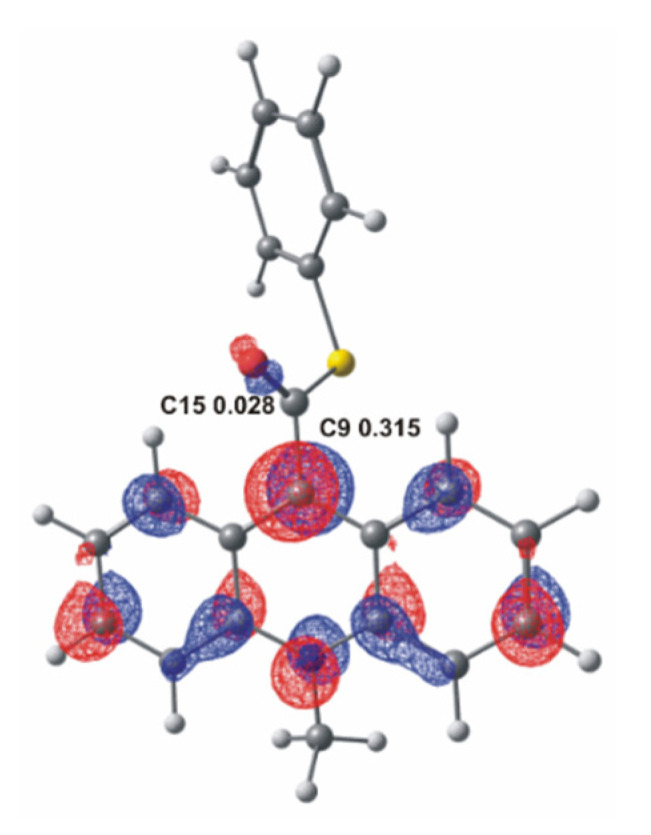
LUMO molecular orbital of acridinium thioester (a) with the values of LCAO coefficient of the p_z_ LUMO orbital at C9 and C15 atoms.

As is known from previous studies [12,13,17], acridinium derivatives may react with oxidants in alkaline media relatively easily, yielding electronically excited products that are able to emit light. In the environment of the reaction (alkaline media), an oxidant dissociates to the anionic form and then reacts with acridinium cation, which initiates the chemiluminescence reaction. The anion of the oxidant is the nucleophile and it will attack the electrophilic center in the acridinium cation. To better understand where the electrophilic center of the molecule is located, it can refer to the frontier molecular orbital theory [45]. According to this theory, which describes the electronic structure of molecules, the bonding between the molecules approximates the molecular orbitals as linear combinations of atomic orbitals (LCAO). Therefore, the Lowest Unoccupied Molecular Orbital (LUMO) distribution of an electrophile determines the molecular center sensitive to nucleophilic attack. The computationally predicted LUMO orbitals of acridinium thioester with indicating potential electrophilic centers: the endocyclic C9 and the carbonyl C15 atom, and the values of the LCAO coefficient of the p_z_ atomic orbital in LUMO orbitals at these centers are shown in Figure 1. The average value of the calculated LCAO coefficient of the p_z_ LUMO orbital of the investigated ATEs is equal to 0.317 at the endocyclic C9 atom and 0.014 at the carbonyl C15 atom (for more details see Table S1 in the Supplementary Materials). The ca. 20 times higher LCAO coefficient at C9 than at C15 atom implies that C9 rather than C15 makes the site of the primary nucleophilic attack of the anionic form of the oxidants. The Mulliken partial charges at the same potential electrophilic center are also shown in Table S1 in the Supplementary Materials. The computational result of Mulliken charge at the C9 is lower than at the C15 (ca. 2 times). These results suggest that the population analysis shows which atom has the largest deficit of the negative charge and does not indicate well the location of the electrophilic center. In this subject, the more appropriate approach is to use the frontier molecular orbital theory.

### 2.3. Mechanism of Chemiluminescence

**Scheme 4:**
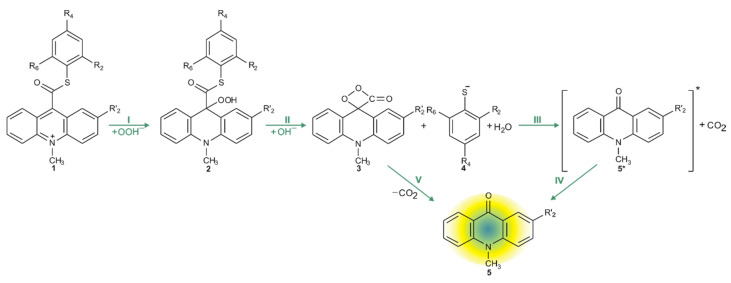
CL pathway of acridinium thioester (ATE).

**Scheme 5:**
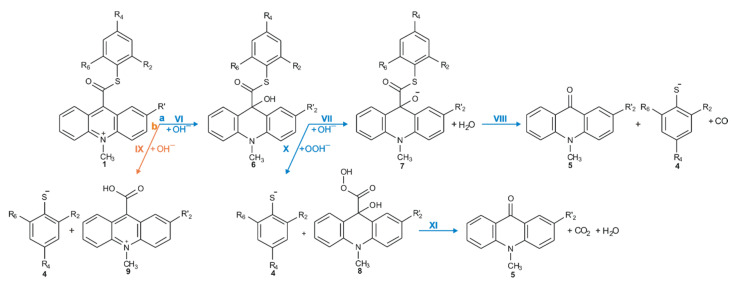
Non-CL pathways of ATEs: (**a**, blue) the transformation of the acridinium cation to the non-emitting product, and (**b**, orange) the hydrolysis of the acridinium molecule.

Considering the reaction of acridinium derivatives with oxidants (e.g., OOH^−^) in alkaline environment (e.g., in presence of OH^−^), two processes should be considered: the reaction leading to the light emission (chemiluminescence) and other competitive processes (so-called dark processes or non-CL processes). Scheme 4 and Scheme 5 present the processes that account for CL and non-CL processes, respectively (the summary of all investigated processes is shown in Scheme S1 in the Supplementary Materials). The theoretically predicted thermodynamic data for all reaction steps are given in Table 1, while Table 2 contains kinetic characteristics of the selected steps. Additionally, the density functional theory (DFT)-optimized geometries originating from the acridine molecules produced by the reaction of ATEs with OOH^−^ or OH^−^ are shown in Table S3 (lowest energy and transition state structures) in the Supplementary Materials.

#### 2.3.1. Chemiluminescence Pathway

The chemiluminescence reaction begins from nucleophilic attack of anionic form of oxidant (e.g., OOH^−^) at the C9 atom on acridinium moiety (step I). In the next steps, the addition product 2 reacts with an OH^−^ ion and after elimination of the thiophenyl anion, forms cyclic intermediate 3 (step II), which after the elimination of carbon dioxide leads to the energy-rich 10-methyl-9-acridinone (5 *, step III), and then followed by the relaxation of electronically excited 10-methyl-9-acridinone to the ground state with the emission of light (step IV).

**Figure 2:**
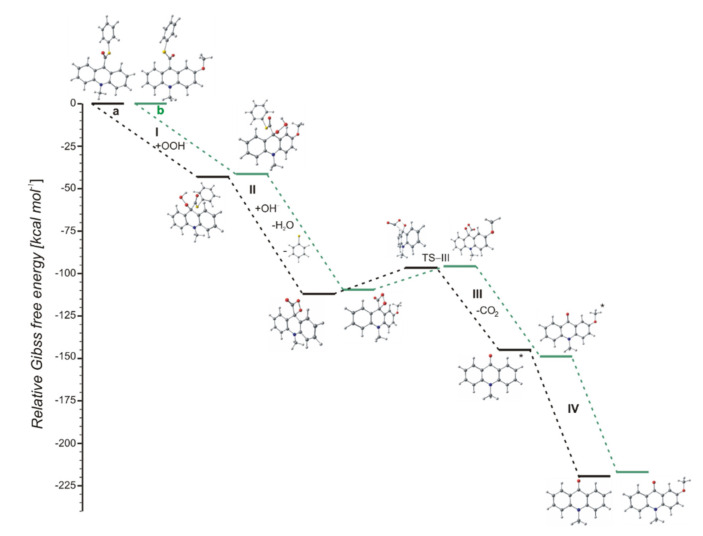
Relative Gibbs free energies of the chemiluminescence (CL) reaction steps (CL pathway) of acridinium thioesters: (**a**, black) unsubstituted and (**b**, green) substituted in the acridine moiety.

The computationally predicted thermodynamic data (Table 1) indicated that all proposed CL steps (I–V) are possible for all investigated compounds in gaseous and aqueous phases. Our calculations reveal that the activation barriers (Table 2) predicted for step III (formation of electronically excited product) are similar to the activation barriers of the same step predicted for acridinium esters (ca. 15.4 kcal mol^−1^) [12]. Boużyk et al. have shown that the necessary value of energy to cause the electronic excitation of 10-methyl-9-acridinone is 88.2 kcal mol^−1^ [46]. Our calculation reveal that the energy released in step V (Table 1) exceeds that which is necessary to cause the electronic excitation of product of CL reaction. Comparing the thermodynamic and kinetic results of CL pathway (Table 1 and Table 2, steps I–V) of unsubstituted (compounds a–h) and substituted (compounds i–l) in the acridine moiety derivatives, no significant differences in the values of enthalpies and Gibbs free energies can be seen. However, after analyzing the Gibbs free energy profiles of the full reaction leading to the generation of light (Figure 2) for acridinium thioesters without any substituent in the acridine moiety (derivative a) and with the methoxy group in the 2 position in the acridine moiety (derivative i), one can conclude that the addition of an electron-donor substituent, such as –OCH_3_, into the acridine moiety reduces the activation barrier with similar or slightly lower values of thermodynamic characteristics of individual steps of CL pathway.

Comparing the chemiluminescent properties of acridinium thioesters, the nature of the decomposition of cyclic intermediates (structure 3, Scheme 4) should be considered. This entity has two important bonds (O–O and C–C), which have an effect on the transformation in the electronically excited product—10-methyl-9-acridinone. In geometrical terms, the bond lengths of C–C and O–O are 1.532 Å and 1.491 Å, respectively and both appear to be covalent. The VDD population analysis results also present a typical distribution of charges (see Table S2 in the Supplementary Materials)—oxygen atoms have a negative charges and carbon atoms have positive charges. It can be concluded that the combination of negative charges of both oxygen atoms and the small difference between charge indicates a repulsive electrostatic interaction between oxygen atoms. In the case of carbon atoms, the charge differences are significantly higher, which also indicates a repulsive effect of interaction, but attenuated by the polarization effect. To better understand the nature of intramolecular interactions in a dioxetanone entity, the calculations were performed using the quantum theory of atoms in molecules (QTAIM) [47]. The topology of the electron localization function using a bond critical point (BCP) (see Table S2 in the Supplementary Materials) indicates that the C–C bond is a typical covalent bond (combination of negative value of Laplacian (∇^2^*ρ*(*r*)) with high value of electron density distribution (*ρ*(*r*)) and the O–O bond is charge-shifted (combination of positive value of Laplacian (∇^2^*ρ*(*r*)) with high value of electron density distribution (*ρ*(*r*)) [48,49]. To determine σ or π bond character, the values of the ellipticity (ε) were analysed (see Table S2 in the Supplementary Materials). The analysis of these parameters demonstrates that both O–O and C–C bonds represent a sigma bond character (larger values (>0.1) indicate π bond character and lower values indicate σ bond character [50]). However, the value of ellipticity for O–O bond is very close to the value that indicates π bond character. Finally, the ratio of –(G(r_b_)/V(r_b_)) was used as a criterion of the nature of bonds (Table S2, Supplementary Material). For O–O bond, value of these parameters demonstrate covalent bond, and for C–C bond, the –(G(r_b_)/V(r_b_)) ratio is lower than the range indicating partially covalent bond (larger values (–(G(r_b_)/V(r_b_)) > 1.0) indicate noncovalent bond character and values in the range (0.5 < –(G(r_b_)/V(r_b_)) < 1.0) indicate partially covalent bond character [51]). The results of calculation using quantum theory of atoms in molecules does not clearly indicate the covalent nature of the O–O and C–C bonds in the cyclic intermediates. On the other hand, the results of computational study on excited states of 1,2-dioxetane suggest that in the first step the O−O bond is broken and the molecule enters in a region of biradical character, and next the chemiexcitation occurs concomitantly with C–C bond breaking [52,53].

#### 2.3.2. Non-CL Processes

**Figure 3:**
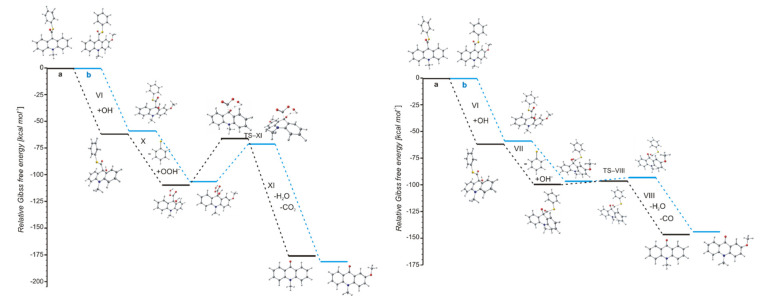
Relative Gibbs free energies of the reaction steps of two possibilities of non-CL pathways of acridinium thioesters: (**a**, black) unsubstituted and (**b**, blue) substituted in the acridine moiety.

The values of quantum yield of chemiluminescence of acridinium derivatives (2–7% in aqueous environments [54,55]) indicate such a small portion of chemiluminogen is transformed into electronically excited product, which leads to the light emission. Unfortunately, most of the molecules undergo competitive reactions (the dark processes), that do not lead to the formation of electronically excited 10-methyl-9-acridinone, but 10-methyl-9-acridinone in the ground state. The first group of transformation of acridinium derivatives, which not leading to the electronically excited products, is all reactions, which transform of the acridinium cation to the non-emitting product (Scheme 5, pathway starting with step VI), and the second group is the hydrolysis of the acridinium molecule (Scheme 5, pathway XI). Both above-mentioned groups significantly reduce the chemiluminescence quantum yield of acridinium derivatives. The first group of the dark processes starts from the nucleophilic attack of OH^−^ at the endocyclic carbon atom (C9) of acridine moiety, creating of so-called ‘pseudobase’. The computationally predicted thermodynamic data (Table 1) indicated that the two steps of competitive processes—leading to the CL (step I, addition of the OOH^−^) and the formation of the ‘pseudobase’ (step VI, addition of the OH^−^) are possible for all of investigated ATEs. Considering the values of Gibbs’ free energies in aqueous environment, one can be noted that the addition of OH^−^ into position 9 of the acridine moiety is thermodynamically more preferred than the OOH^−^. In this case, the kinetic factors will be determined the preference for the addition of ions. All proposed non-CL steps (VI–X) are possible for all investigated compounds in gaseous and aqueous phases. Our calculations reveal that the activation barriers (Table 2) predicted for step VIII are relatively small (in the range of 0.3–2.8 kcal mol^−1^). On the other hand, the thermodynamic data show that the energy released in this step exceeds that which is necessary to cause the electronic excitation of product of CL reaction. Therefore, steps VI–VIII will not lead to the formation of electronically excited 10-methyl-9-acridinone. The thermodynamic data suggest that step X can lead to the formation of product in electronically excited state (Table 1), but the activation barriers (Table 2) predicted for this step are very high (43.3 and 36.1 kcal mol^−1^ for unsubstituted (compounds a–h) and substituted (compounds i–l), respectively), so the step X will not occur. In this situation, both the above pathways may account for the dark transformation of ATEs. Comparing the thermodynamic and kinetic results of non-CL pathway, starting with the formation of ‘pseudobase’, of unsubstituted (compounds a–h) and substituted (compounds i–l) in the acridine moiety derivatives (Table 1 and Table 2, steps VI–X), no significant differences in the values of enthalpies and Gibbs free energies can be seen. The Gibbs free energy profiles of the full non-CL pathway (Figure 3) for acridinium thioesters without any substituent in the acridine moiety (derivative a) and with the methoxy group in the 2 position in the acridine moiety (derivative i), show that the addition of an electron-donor substituent, such as –OCH_3_, into the acridine moiety reduces the activation barrier with similar or slightly lower values of thermodynamic characteristics of individual steps of CL pathway.

Among the compounds investigated, f (electron-acceptor substituent in 4 position of thiophenoxy group), g (two electron-acceptor substituents in 2 and 6 positions of thiophenoxy group) and h (two electron-donor substituents in 2 and 6 positions and electron-acceptor substituent in 4 position of thiophenoxy group) pay attention. The thermodynamic results indicate decrease of Gibbs free energy values compared to the same values for other compounds investigated. The highest decrease can be seen in the case of II (formation of cyclic intermediate 3) and IX (hydrolysis of acridinium cation) steps. It can be assumed, that the addition of an electron-acceptor substituents in 2 and 6 positions or an electron-donor substituents in 2 and 6 position while an electron-acceptor substituent in 4 position, will affect the more efficient transformation into electronically excited product, but also sensitizes to the hydrolysis of ATEs.

Considering the utilitarian perspectives of the studied compounds, we compared the energetical values of the main steps (the chemiluminescence pathway and the formation of the pseudobase as affecting the whole process) between acridinium thioesters and currently used in the analysis–acridinium esters. Figure S2 in the Supplementary Materials presents the Gibbs free energy profiles of the CL pathway and the first step of the non-CL pathway (formation of the pseudobase) of acridinium thioesters and acridinium esters. The results show slight differences of the values of Gibbs free energies for step I (addition of OOH^−^ at the C9 atom on acridinium moiety; −44.8 and −43.1 kcal mol^−1^ for AE and ATE, respectively) and VI (formation of the pseudobase; −62.8 and −61.9 kcal mol^−1^ for AE and ATE, respectively) and significant differences of the values of Gibbs free energies for step II (formation of the cyclic intermediate; −47.6 and −69.0 kcal mol^−1^ for AE and ATE, respectively) between acridinium esters and acridinium thioesters. A comparison of these values suggests the more thermodynamically preferred formation of the cyclic intermediate for acridinium thioesters, and thus its decomposition to the electronically excited product. It can be concluded that acridinium thioesters will be more efficient chemiluminogens than acridinium esters.

### 2.4. Electronically Excitation

The mechanism of chemiluminescence reaction of ATEs indicates that CL pathway leads to energy-rich 10-methyl-9-acridinone, which returning to the ground state emits light. In our previous studies on acridinium esters [12,17], we reported formation of electronically excited product after elimination of phenyl carbonate anion [17] or firstly, phenoxy anion and secondly, carbon dioxide from dioxetane intermediate [12]. As mentioned in Section 2.3.1., the formation of energy-rich 10-methyl-9-acridinone in the CL reaction of acridinium thioesters follows by the transformation to the dioxetane intermediate after the elimination of the thiophenyl anion, and then the elimination of the carbon dioxide (Scheme 4, step III and IV). The formation of cyclic intermediate 3 (Scheme 4) and then its decomposition is a rate determining step of the chemiluminescence.

**Figure 4:**
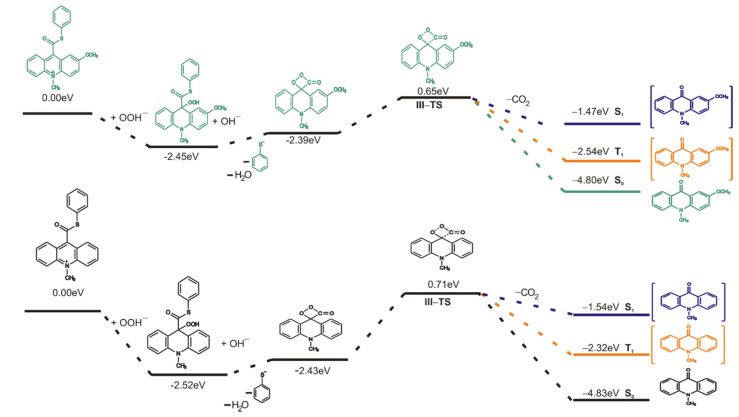
Energy diagram of the chemiluminescence reaction in the aqueous phase with energy of the lowest triplet (T_1_) and singlet (S_1_) states.

To better understand the nature of an electronically excited product, an investigation of excited states was carried out using the time dependent (TD) DFT calculation. As is known, the intersystem crossing (ISC) mechanism, which is the nonradiative transition between two electronic states of different multiplicity, plays an important role in photochemistry and explains well the properties of organic chromophores [56,57]. Taking into account the previous reports about the decomposition mechanism of 1,2-dioxetane [52], 1,2-dioxetanedione [58] and acridinium esters [5], the spin-orbit coupling between the S_0_ and the T_1_ states occurs along the chemiluminescence reaction pathway. The computed energy profiles on the singlet (S_1_), triplet (T_1_) and ground (S_0_) states started from the transition state of decomposition of cyclic intermediate 3 (TS-III) show that the potential energy curve of the triplet state (T_1_) lays closer than the potential energy curve of the singlet state (S_1_) to the potential energy curve of the ground state (S_0_). Figure 4 and Figure S3 in the Supplementary Materials show the energy diagram of CL pathway in aqueous and gaseous phases, respectively, including thermal decomposition (S_0_) and formation of excited states (S_1_ and T_1_). The vertical excitation energies in aqueous phase of 10-methyl-9-acridinone in equilibrium geometry are 2.51 and 2.26 eV for T_1_ state and 3.29 and 3.33 eV for S_1_ state for unsubstituted and substituted in the acridine moiety, respectively. Therefore, the emission energy from T_1_ corresponds well to experimental results that show the maximum emission speak at 451 nm (2.75 eV) for 10-methyl-9-acridinone [46]. It should be noted that the emission energy from S_1_ state in gaseous phase for unsubstituted in the acridine moiety product of the chemiluminescence reaction are higher than that for the product substituted in position 2 in acridine moiety (4.82 and 3.23 eV, respectively). This difference decreases in the aqueous phase as indicated above. According to this, the photoemission of the mixed state (from T_1_ and S_1_ states) of 10-methyl-9-acridinone in water solution is expected. On the other hand, the phosphorescence will be quenched in solution, and we expect that it may not play a significant role in the chemiluminescence of acridinium derivatives.

## 3. Materials and Methods

Unconstrained geometry optimizations of isolated molecules were carried out at the DFT [59] and TD DFT [60] level of theory to determine ground and excited states, respectively. All the calculations were conducted using the popular Becke’s three-parameter hybrid functional B3LYP [61,62] and the 6-31G** basis set [63,64] with the Gaussian09 package [65]. B3LYP functional is known to produce reliable results [5,12,17] for the thermodynamic data and have been proven to provide accurate qualitative results for the mechanism of chemiluminescence reaction of investigated molecules. After completion of each optimization, the Hessian (second derivatives of the energy as a function of the nuclear coordinates) was calculated and checked for positive definiteness to assess whether the structures were true minima. The solvent (water) effect included in the DFT calculations at the level of the polarizable continuum model (PCM) was used to mimic water environment (UAHF radii were used to obtain the molecular cavity) [66,67]. The transition energies for the electronically excited product of chemiluminescence reaction of acridinium thioesters—10-methyl-9-acridinone—were calculated from an excited state optimized structure using the state-specific approach. The Voronoi deformation density (VDD) population analysis was performed by using the Multiwfn code [68] with the same level of theory and basis set as geometry optimization. The electron density at the bond critical point (BCP) were analysed using the quantum theory of atoms in molecules (QTAIM) methodology [47]. Visualization of the results of the calculations of equilibrium structures of all molecules was made using the ChemCraft software [69].

The enthalpies (Δ_r,298_*H*^0^) and Gibbs free energies (free energies in the case of DFT(PCM)) (Δ_r,298_*G*^0^) of the reactions (r), as well as the enthalpies (Δ_a,298_*H*^0^) and Gibbs free energies (free energies in the case of DFT(PCM)) (Δ_a,298_*G*^0^) of activation (a) were calculated by following the basic rules of thermodynamics [44]. The rate constants (_298_*k*^0^) for the gaseous phase reactions were obtained by applying the equation:

(1) $${}_{298}k^{0}=\frac{RT}{Nh}\exp[-\Delta_{a,298}G^{0}/(RT)]$$

Resulting from the transition state theory, and reaction completion time (_298_*τ*_99_) from the formula:

(2) $${}_{298}\tau_{99}=\ln100/298k^{0}$$

where *R*, *T*, *N* and *h* denote the gas constant, temperature (298.15 K), Avogadro number and Planck’s constant, respectively [44].

## 4. Conclusions

Due to the rapid growth of population and factors which negatively affect health, the increasing number of diseases motivates the intensive search for new effective diagnostic methods. One of the promising immunological diagnostic procedures is chemiluminescent labelling, in which efficient chemiluminogens are used. Searching for new chemiluminogens with better features, especially higher quantum yields, is the main aim of this work. As acridinium esters are an efficient chemiluminogens, we modelled new group of compounds based on acridinium moiety—10-methyl-9-thiophenoxycarbonylacridinium derivatives. The proposed chemiluminescence reaction mechanism of these compounds was based on our previous computational and experimental studies [12,13,17]. The CL reaction begins with the nucleophilic attack of hydroperoxide anion, then forms highly strained dioxetanone ring after addition of hydroxyl anion, and finally energy-rich 10-methyl-9-acridinone as a result of decomposition of cyclic intermediate. The step, in which the decomposition of the cyclic dioxetanone ring is observed, is the rate-determining step in the whole process. All the considered compounds were computationally proven to easily transform into electronically excited products as a result of the chemiluminescence reaction. However, all investigated derivatives could be divided into two groups: unsubstituted and substituted in the acridine moiety. The addition of an electron-donor substituent, e.g., methoxy group, into the acridine moiety reduces the activation barrier with almost similar values of thermodynamic characteristics of individual steps of CL pathway. Additionally, the studied acridinium derivatives are susceptible to two reactions competing with chemiluminescence: formation of ‘pseudobase’ and hydrolysis. The computational results show that the possible pathway of the reaction, CL or one of the competitive pathways, seems to be exclusively dependent on kinetic factors. The most promising are the addition of an electron-acceptor substituents in the 2 and 6 positions or an electron-donor substituent in the 2 and 6 position while an electron-acceptor substituent in the 4 position moves into the thiophenoxy group. These substitutions will affect the more efficient transformation into an electronically excited product, but also sensitize it to the hydrolysis of investigated compounds. The computationally predicted emission energy from T_1_ for electronically excited products of CL reaction—10-methyl-9-acridinone corresponds well to experimental results. The theoretical studies of the excited state indicate that the photoemission of the mixed state (from T_1_ and S_1_ states) of 10-methyl-9-acridinone in water solution is expected.

To sum up, with the use of a simple computational approach, we were able to identify potential chemiluminogens for further experimental studies. Synthesis, followed by studies of their chemiluminescent properties, and ultimately in chemiluminescent labelling, are further steps to confirm their potential applicability in immunodiagnostics.

## Figures and Tables

**Table 1:** Thermodynamic data ^1^ of the elementary steps of the reaction of ATE with OOH^−^ and OH^−^.

Step No.	Compound	Gaseous Phase		Aqueous Phase	Step No.	Compound	Gaseous Phase		Aqueous Phase
		Δ_r,298_*H*^0^	Δ_r,298_*G*^0^	Δ_r,298_*G*^0^			Δ_r,298_*H*^0^	Δ_r,298_*G*^0^	Δ_r,298_*G*^0^
I	a	−166.2	−153.0	−43.1	II	a	−80.1	−93.2	−69.0
	b	−166.0	−153.1	−43.2		b	−86.3	−99.5	−73.3
	c	−167.1	−152.7	−44.9		c	−80.3	−93.9	−67.7
	d	−165.8	−152.0	−43.8		d	−83.3	−97.2	−67.5
	e	−163.6	−150.0	−42.3		e	−75.8	−89.7	−67.8
	f	−171.1	−157.4	−44.0		f	−101.7	−115.0	−79.3
	g	−166.0	−151.6	−44.1		g	−92.1	−105.1	−76.2
	h	−171.0	−158.0	−44.6		h	−102.2	−115.1	−78.9
	i	−162.3	−149.4	−41.3		i	−80.1	−93.1	−68.7
	j	−162.5	−149.5	−41.2		j	−85.9	−99.6	−73.3
	k	−162.6	−148.9	−41.6		k	−80.9	−94.5	−68.9
	l	−161.5	−148.2	−41.2		l	−83.7	−97.3	−68.2
III	a–h	−16.5	−27.2	−33.2	IV	a–h	−78.1	−77.6	−73.3
	i–l	−23.0	−32.6	−38.7		i–l	−72.3	−71.8	−68.2
V	a–h	−94.6	−103.9	−106.5	VII	a	−64.7	−63.9	−37.4
	i–l	−95.3	−104.5	−107.0		b	−76.2	−75.5	−38.9
VI	a	−197.1	−187.5	−61.9		c	−72.1	−72.6	−37.9
	b	−188.9	−179.5	−61.7		d	−73.6	−74.3	−37.4
	c	−189.9	−178.6	−62.3		e	−64.1	−63.8	−37.1
	d	−188.8	−178.5	−62.3		f	−83.0	−83.0	−41.2
	e	−186.4	−176.0	−60.7		g	−80.9	−81.3	−44.7
	f	−194.3	−183.7	−62.6		h	−82.4	−83.4	−40.4
	g	−188.9	−177.6	−62.1		i	−73.6	−73.2	−38.0
	h	−194.3	−183.6	−62.9		j	−77.3	−77.0	−39.3
	i	−185.1	−175.4	−59.1		k	−73.2	−74.0	−37.7
	j	−184.7	−175.3	−59.5		l	−74.4	−74.7	−37.5
	k	−185.7	−174.9	−60.3	IX	a	−92.0	−93.2	−68.9
	l	−184.8	−174.9	−60.5		b	−97.9	−99.6	−73.3
VIII	a	−13.2	−37.1	−48.6		c	−93.0	−93.6	−69.4
	b	−15.9	−39.8	−51.7		d	−94.8	−96.1	−68.0
	c	−14.0	−37.6	−48.2		e	−85.0	−86.7	−66.8
	d	−15.4	−38.7	−47.3		f	−118.6	−119.4	−80.1
	e	−11.7	−35.5	−48.0		g	−103.7	−103.7	−77.1
	f	−24.3	−48.1	−55.3		h	−118.9	−120.2	−80.3
	g	−16.9	−40.0	−49.3		i	−92.3	−93.4	−68.9
	h	−25.2	−48.4	−55.9		j	−98.3	−100.1	−73.4
	i	−13.0	−36.6	−49.2		k	−93.3	−94.4	−69.4
	j	−15.8	−39.6	−52.0		l	−95.1	−96.5	−68.3
	k	−13.9	−37.3	−48.8	X	a	−60.3	−58.8	−46.9
	l	−15.3	−38.7	−47.6		b	−74.5	−73.2	−51.4
XI	a–h	−83.5	−103.8	−109.9		c	−68.5	−68.0	−46.9
	i–l	−84.6	−104.8	−110.6		d	−71.4	−70.8	−45.5
						e	−64.1	−63.8	−45.9
						f	−89.7	−88.9	−57.3
						g	−80.2	−79.2	−54.7
						h	−90.0	−89.6	−57.1
						i	−68.0	−66.7	−47.2
						j	−74.5	−73.5	−51.4
						k	−68.4	−68.2	−46.6
						l	−71.4	−70.3	−45.2

^1^ Δ_r,298_*H*^0^ and Δ_r,298_*G*^0^ (both in kcal mol^−1^), respectively, represent the enthalpy and Gibbs’ free energy (gaseous phase) or free energy (aqueous phase) of the reaction corresponding to a given step number at standard temperature and pressure.

**Table 2:** Kinetic characteristics ^1^ of the selected steps of the CL reaction of ATEs.

Step No.	Compound	Gaseous Phase			Aqueous Phase
		Δ_r,298_*H*^0^	Δ_r,298_*G*^0^	_298_*k*^0^(_298_*τ*_99_)	Δ_r,298_*G*^0^
TS-III	a–h	12.9	14.2	2.5 × 10^2^ (1.9 × 10^−2^)	15.6
	i–l	12.8	14.2	2.4 × 10^2^ (1.9 × 10^−2^)	14.5
TS-VIII	a	2.1	0.3	4.1 × 10^12^ (1.1 × 10^−12^)	2.1
	b	2.3	1.2	8.9 × 10^11^ (5.2 × 10^−12^)	1.6
	c	1.9	0.9	1.3 × 10^12^ (3.4 × 10^−12^)	2.4
	d	2.4	1.7	3.8 × 10^11^ (1.2 × 10^−11^)	0.5
	e	2.4	1.1	1.0 × 10^12^ (4.5 × 10^−12^)	2.1
	f	1.1	0.4	3.2 × 10^12^ (1.5 × 10^−12^)	1.3
	g	3.1	2.2	1.5 × 10^11^ (3.1 × 10^−10^)	2.8
	h	0.5	0.4	3.4 × 10^12^ (1.3 × 10^−12^)	0.3
	i	2.7	1.5	4.8 × 10^11^ (9.5 × 10^−12^)	2.4
	j	2.3	1.2	8.6 × 10^11^ (5.4 × 10^−12^)	2.0
	k	2.5	1.5	4.9 × 10^11^ (9.3 × 10^−12^)	2.0
	l	2.5	0.8	1.7 × 10^12^ (2.7 × 10^−12^)	2.7
TS-XI	a–h	43.6	45.3	4.1 × 10^−21^(1.1 × 10^21^)	43.3
	i–l	35.1	36.9	6.0 × 10^−15^(7.7 × 10^14^)	36.1

^1^ Δ_a,298_*H*^0^ and Δ_a,298_*G*^0^ (both in kcal mol^−1^), respectively, represent the enthalpy and Gibbs’ free energy (gaseous phase) or free energy (aqueous phase) of activation at standard temperature and pressure; _298_*k*^0^ (in s^−1^) and _298_*τ*_99_, respectively, denote the rate constant and the time after which the reaction is 99% complete.

## Supplementary Materials

The following are available online at https://www.mdpi.com/1422-0067/21/12/4417/s1. Figure S1. The VDD population analysis results of the investigated dioxetanone molecules (a) unsubstituted and (b) substituted in the acridine moiety. Figure S2. Relative Gibbs free energies of the CL pathway (reaction steps: I–IV) and non-CL step VI (formation of pseudobase) of acridinium thioesters: (a, black) and acridinium esters (c, violet). Figure S3. Energy diagram of chemiluminescence reaction in the gaseous phase with energy of the lowest triplet (T1) and singlet (S1) states. Scheme S1. Pathways of the reactions of ATE with OOH− and OH− (CL pathway (green arrows), non-CL pathways: transformation of ATE to the non-emitting product (blue arrows) and hydrolysis of ATE (orange arrows)). Table S1. The calculated Mulliken atomic charges and LCAO coefficient of the pZ LUMO orbital at the endocyclic C9 and the carbonyl C15 atoms. Table S2. The VDD population analysis results of the investigated dioxetanone molecules (a) unsubstituted and (b) substituted in the acridine moiety. Table S3. Cartesian coordinates and structures of the lowest energy and transition state structures of investigated molecules.

## Abbreviations

AE	Acridinium ester
ATE	Acridinium thioester
BCP	Bond critical point
CL	Chemiluminescence
DFT	Density functional theory
FT4	Thyroxine hormone
HIV	Human immunodeficiency virus
ISC	Intersystem crossing
LCAO	Linear combination of atomic orbitals
LUMO	Lowest Unoccupied Molecular Orbital
PCM	Polarizable Continuum Model
QTAIM	Quantum theory of atoms in molecules
TD DFT	Time dependent density functional theory
TS	Transition State
TSH	Thyroid-stimulating hormone
VDD	Voronoi deformation density
