# Supplementary material for: Computational Insights on the Mechanism of the Chemiluminescence Reaction of New Group of Chemiluminogens—10-Methyl-9-thiophenoxycarbonylacridinium Cations
Source: Int J Mol Sci. 2020 Jun 21;21(12):4417. doi: 10.3390/ijms21124417 (PMC7420290; doi:10.3390/ijms21124417)

Supplementary Materials

Computational Insights on the Mechanism of the Chemiluminescence Reaction of New Group of Chemiluminogens – 10-Methyl-9-thiophenoxycarbonylacridinium Cations

Milena Pieńkos and Beata Zadykowicz*

Faculty of Chemistry, University of Gdańsk, Wita Stwosza 63, 80-308 Gdańsk, Poland

Correspondence: beata.zadykowicz@ug.edu.pl

Scheme S1. Pathways of the reactions of ATE with OOH^- and OH^- (CL pathway (green arrows), non-CL pathways: transformation of ATE to the non-emitting product (blue arrows) and hydrolysis of ATE (orange arrows)).

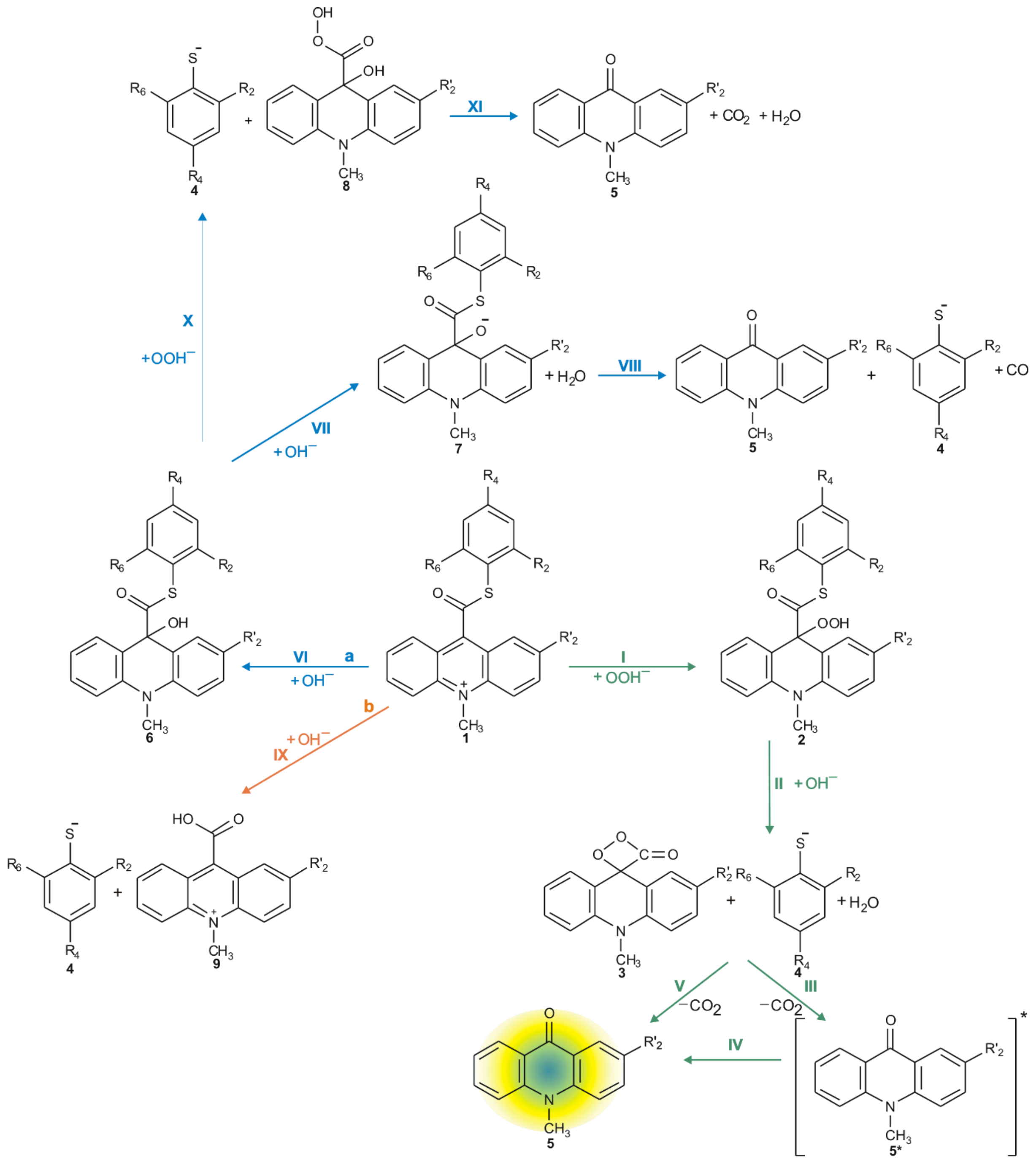

Table S1. The calculated Mulliken atomic charges and LCAO coefficient of the p_Z LUMO orbital at the endocyclic C9 and the carbonyl C15 atoms.

		Distribution charges				
**Compound**		**Mulliken population**			**p_Z LUMO**	
	**atom**	**C9**	**C15**		**C9**	**C15**
**a**		0.08203	0.15337		0.31536	0.02752
**b**		0.07367	0.15911		0.31483	0.02922
**c**		0.08186	0.15316		0.31051	0.02844
**d**		0.07880	0.14881		0.29330	0.02792
**e**		0.07246	0.16747		0.31103	0.02353
**f**		0.07734	0.15280		0.31551	0.03508
**g**		0.06877	0.15230		0.31847	0.02548
**h**		0.07893	0.15328		0.30948	0.03537
**i**		0.07028	0.15414		0.30883	0.03460
**j**		0.06231	0.16334		0.30767	0.03941
**k**		0.07044	0.15451		0.30670	0.03618
**l**		0.06614	0.14913		0.29821	0.03732
	AVE	0.074	0.155	AVE	0.309	0.032
					$\frac{0.309}{0.032} = 9{,}8$ ratio≈1:10	

Figure S1. and **Table S2.** The VDD population analysis results of the investigated dioxetanone molecules (a) unsubstituted and (b) substituted in the acridine moiety.

Results of the VDD population analysis, at the PCM-B3LYP/6-31G (d, p) level of theory, for all dioxetanones

a	O_{29}	O_{30}	C_{11}	C_{15}
	-0,088273	-0,120393	0,199079	0,05318
b	O_{16}	O_{17}	C_{10}	C_{15}
	-0,0918	-0,1227	0,0516	0,1991

$\nabla^2\rho(r)$, $\rho(r)$, ε, and-($G(r_b)/V(r_b)$) values for the C_{15}-C_{11}, O_{29}-O_{30}, C_{10}-C_{15}, O_{16}-O_{17} *BCP, for all dioxetanones*

	bonds	$\nabla^2\rho(r)$	$\rho(r)$	ε	$-(G(r_b)/V(r_b))$
a	C_{15}-C_{11}	-0,6277	0,2584	0,085475	0,2141
	O_{29}-O_{30}	0,0137	0,2561	0,110024	0,5043
b	C_{10}-C_{15}	-0,6248	0,2579	0,0852	0,2147
	O_{16}-O_{17}	0,0157	0,2555	0,1094	0,5052

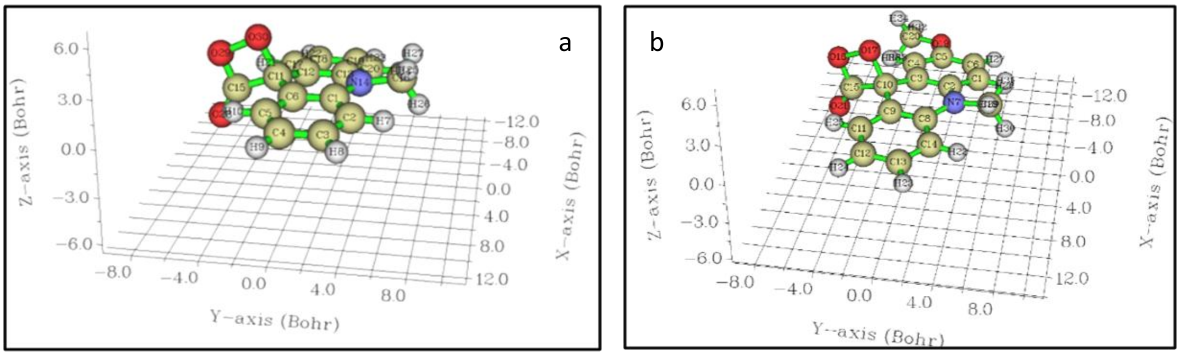

Figure S2. Relative Gibbs free energies of the CL pathway (reaction steps: I–IV) and non-CL step VI (formation of pseudobase) of acridinium thioesters: (a, black) and acridinium esters (c, violet).

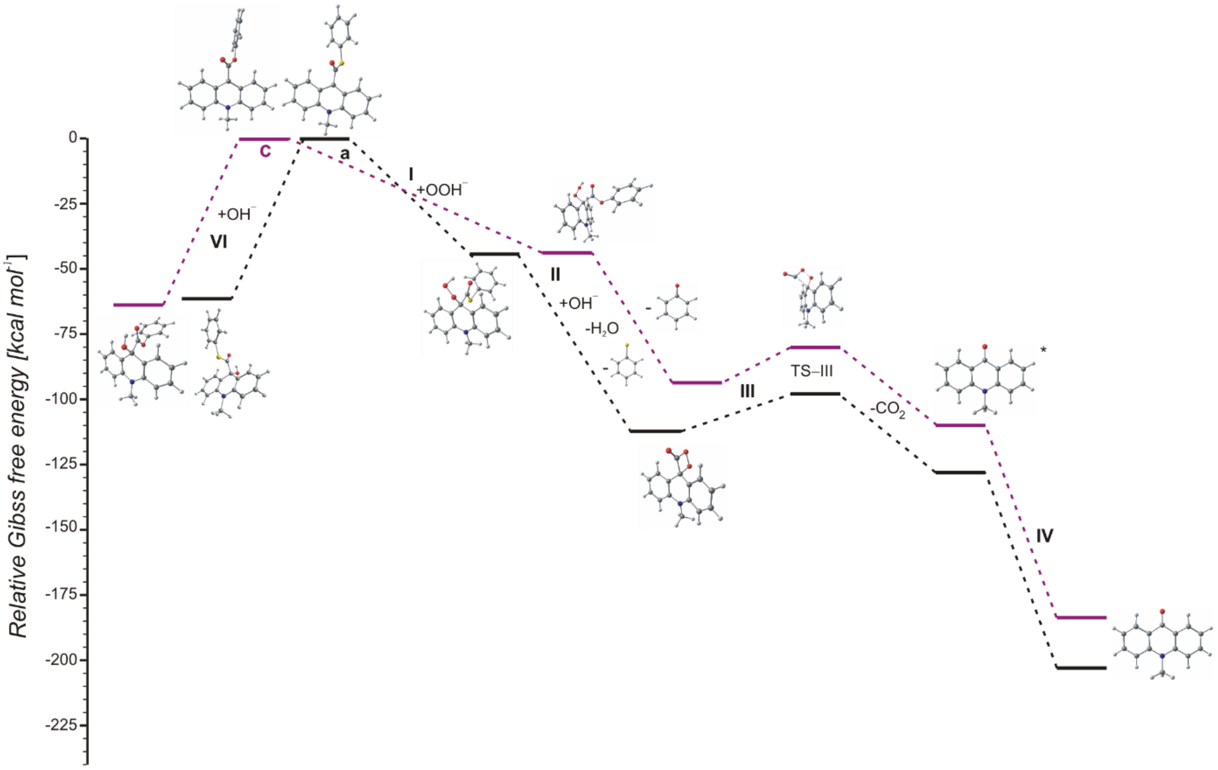

Figure S3. Energy diagram of the chemiluminescence reaction in the gaseous phase with energy of the lowest triplet (T_1) and singlet (S_1) states.

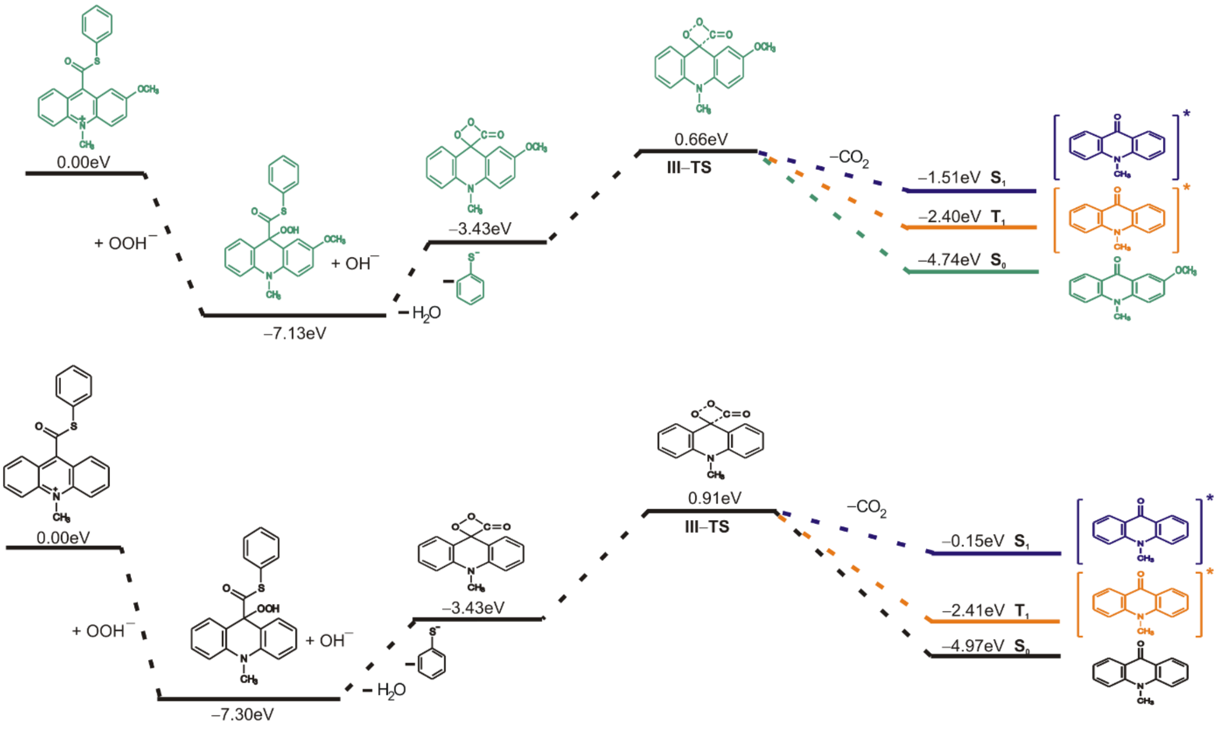

Table S3. Cartesian coordinates and structures of the lowest energy and transition state structures of investigated molecules. list of abbreviations: * – excited product resp. S_1 – first singlet state, T_1 – first triplet state; TS – the number corresponding to the reaction steps

R_2=H, R_4=H, R_6=H, R_2'=H				
**Geom.No**<br>**1 = -1337.85539377 Ha**				
Atom	X	Y	Z	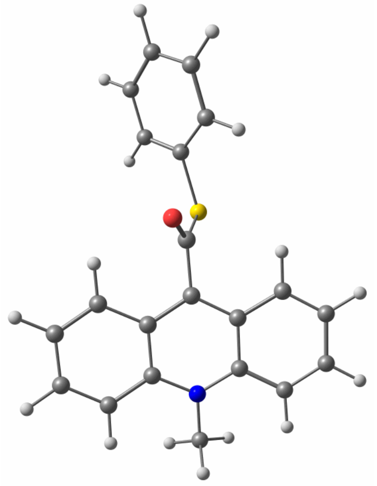
C	0.000005141	0.000002460	0.000001020	
C	-0.000001929	-0.000000641	-0.000002190	
C	-0.000000882	-0.000000015	0.000000137	
C	-0.000002944	0.000000564	0.000000982	
C	-0.000000450	0.000000787	0.000000546	
C	-0.000006599	-0.000001125	0.000000626	
H	-0.000001101	-0.000000764	-0.000000570	
H	-0.000003055	-0.000000329	-0.000000539	
H	-0.000002269	0.000000477	0.000000491	
H	-0.000001364	0.000000259	0.000001420	
C	0.000015223	0.000003447	-0.000002034	
C	-0.000006863	-0.000001665	-0.000003759	
C	0.000002233	-0.000001880	-0.000007022	
N	-0.000010991	-0.000001081	0.000003348	
C	-0.000000290	-0.000023107	0.000012694	
C	0.000006699	-0.000000984	-0.000005459	
C	0.000002648	0.000000434	0.000001377	
C	-0.000002471	-0.000000404	-0.000001200	
C	0.000003016	0.000000107	-0.000005104	
C	0.000000511	0.000000735	-0.000000122	
H	-0.000000536	-0.000001113	-0.000000996	
H	0.000002534	-0.000000481	-0.000001382	
H	0.000001336	-0.000000122	-0.000002317	
H	0.000000205	0.000000181	-0.000002588	
H	-0.000003441	0.000000191	-0.000002211	
H	-0.000002329	-0.000000399	-0.000002776	
H	-0.000001837	-0.000000398	-0.000002815	
S	-0.000022514	0.000012014	0.000016851	
O	0.000005964	0.000007881	-0.000005402	
C	0.000012771	-0.000001242	-0.000019809	
C	-0.000000008	-0.000000720	0.000004706	
C	0.000003825	0.000002531	0.000001326	
C	0.000001526	-0.000003067	0.000001882	
C	-0.000001553	0.000003212	0.000005885	
C	0.000004465	0.000002102	0.000001651	
H	0.000001483	0.000000470	0.000001213	
H	0.000001996	0.000000082	0.000002515	
H	0.000002023	0.000000448	0.000003416	
H	0.000001205	0.000000200	0.000002894	
H	-0.000001377	0.000000949	0.000003315	

Geom. No
2 = -1489.01584057 Ha

Atom	X	Y	Z
C	-0.000000045	-0.000000727	-0.000000761
C	-0.000003028	0.000004438	-0.000004578
C	-0.000001480	0.000000451	0.000000408
C	0.000000890	0.000001176	-0.000000488
C	-0.000000935	0.000001581	0.000000208
C	-0.000001223	0.000000875	-0.000000154
S	0.000000001	-0.000007132	0.000003455
C	-0.000000795	0.000011861	0.000000446
O	0.000002794	-0.000004366	-0.000002700
C	0.000001057	0.000006862	-0.000001836
C	-0.000000744	-0.000003940	-0.000000333
C	-0.000000662	0.000000094	-0.000002026
N	-0.000004089	0.000000872	0.000005389
C	0.000001627	-0.000004223	-0.000005614
C	0.000005551	0.000000190	0.000004100
C	0.000002626	-0.000000078	0.000002229
C	-0.000001237	0.000000587	0.000000300
C	-0.000000008	-0.000001596	-0.000002198
C	0.000002147	0.000001512	0.000003890
C	-0.000002337	-0.000001549	-0.000001283
C	-0.000001505	0.000000906	0.000001310
C	0.000002583	-0.000001493	0.000001718
C	-0.000002798	-0.000000445	-0.000003174
O	0.000005870	-0.000008149	0.000003827
O	-0.000003565	0.000004145	-0.000002323
C	0.000002235	-0.000001201	-0.000001163
H	0.000000372	-0.000001354	0.000001397
H	0.000000021	-0.000001757	0.000000308
H	0.000000079	-0.000001095	0.000000179
H	0.000000236	-0.000000306	0.000000003
H	-0.000000024	-0.000001270	-0.000001903
H	0.000000969	0.000000514	-0.000000262
H	0.000000914	-0.000000269	-0.000000203
H	-0.000000752	-0.000000984	-0.000000152
H	-0.000001597	-0.000000788	0.000001298
H	-0.000000756	-0.000000892	0.000001004
H	-0.000000628	-0.000000305	0.000000115
H	-0.000000642	0.000000804	0.000000377
H	-0.000000994	0.000001066	-0.000000234
H	-0.000000778	0.000001655	-0.000000526
H	-0.000000239	0.000001843	-0.000000839
H	0.000000222	0.000001185	0.000000379
H	0.000000667	0.000001302	0.000000411

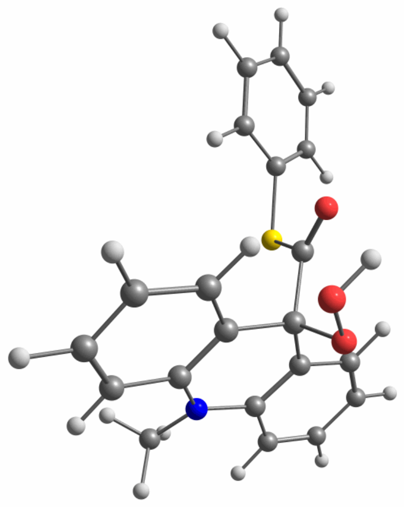

Geom.No
3-TS-III = -858.562621852 Ha

Atom	X	Y	Z
C	0.000001668	0.000001693	-0.000001706
C	-0.000001089	-0.000001851	0.000001165
C	-0.000000367	-0.000001169	-0.000000734
C	-0.000002814	0.000000924	0.000001399
C	-0.000000362	0.000001022	-0.000004683
C	0.000002700	0.000001164	0.000003988
H	-0.000002124	-0.000001075	-0.000000977
H	-0.000000648	-0.000000525	0.000000174
H	-0.000000321	-0.000000268	-0.000000218
H	-0.000000691	0.000002504	0.000001310
C	-0.000017086	0.000054670	0.000000418
C	0.000010024	-0.000005601	0.000004297
C	0.000000008	0.000002454	0.000003356
N	0.000000508	-0.000004494	-0.000002943
C	0.000010417	-0.000023592	-0.000005269
C	0.000002427	0.000000477	0.000001251
C	-0.000002474	0.000002539	-0.000002411
C	0.000001761	0.000000169	-0.000000460
C	-0.000000665	-0.000000821	0.000001710
C	-0.000000299	-0.000001818	-0.000000104
H	-0.000000966	0.000001462	-0.000000044
H	-0.000000352	0.000000638	0.000000331
H	-0.000000532	-0.000000897	0.000000546
H	-0.000000829	-0.000002301	0.000003179
H	0.000001748	-0.000002958	0.000000892
H	-0.000001643	-0.000002553	-0.000000051
H	-0.000000226	-0.000002195	-0.000003133
O	-0.000004137	0.000004980	0.000005157
O	-0.000005940	0.000022784	0.000000099
O	0.000012303	-0.000045363	-0.000006540

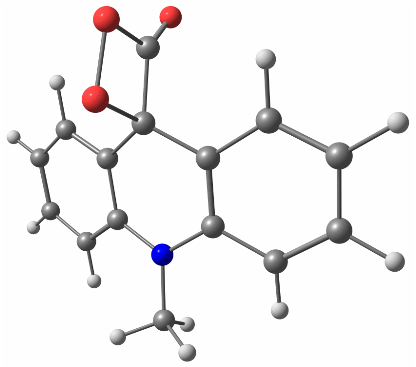

Geom.No
III-TS = -858.539892575

Atom	X	Y	Z
C	-0.000082363	-0.000038892	0.000037500
C	0.000032791	-0.000030846	0.000225778
C	0.000161450	0.000378174	0.000337975
C	-0.000061672	-0.000008641	0.000077345
C	0.000020070	-0.000050955	-0.000037902
C	0.000041833	0.000028367	-0.000007898
C	0.020149337	0.039957791	0.019733155
C	0.000415896	0.000443185	0.000185666
C	0.000238951	0.000089401	-0.000067893
N	-0.000002827	0.000030221	-0.000023235
C	-0.000005093	-0.000073002	0.000063661
C	0.000030045	0.000008444	-0.000002110
C	0.000008916	-0.000041645	0.000009151
C	0.000014720	0.000047682	-0.000148996

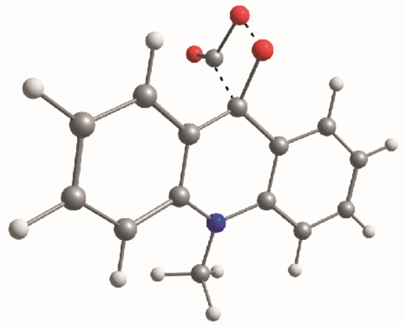

C	0.000039302	-0.000019208	-0.000062086	
O	0.002837912	0.005252437	0.002684135	
O	-0.002888466	-0.005466409	-0.002744421	
C	-0.020652570	-0.035748422	-0.019044535	
O	-0.000356916	-0.004803418	-0.001217003	
H	-0.000000711	0.000005013	-0.000001124	
H	0.000010644	0.000027531	-0.000001426	
H	-0.000002409	0.000000456	-0.000000951	
H	0.000002828	0.000007652	0.000042657	
H	0.000022694	-0.000025094	0.000020409	
H	-0.000003865	0.000024078	0.000013725	
H	0.000002544	0.000004486	0.000002480	
H	-0.000003213	0.000001957	-0.000008981	
H	0.000002325	0.000007882	-0.000023715	
H	0.000020396	-0.000010426	-0.000041999	
H	0.000007450	0.000002202	0.000000638	

**Geom.No<br>4 =-629.885916797 Ha**				
Atom	X	Y	Z	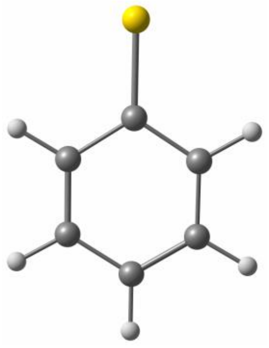
C	0.000056325	0.000000000	-0.000039334	
C	-0.000021910	0.000000000	0.000013498	
C	0.000003350	0.000000000	-0.000002065	
C	-0.000010628	0.000000000	0.000010765	
C	0.000010333	0.000000000	-0.000041218	
C	-0.000027728	0.000000000	-0.000015098	
H	-0.000001505	0.000000000	-0.000005770	
H	0.000000466	0.000000000	-0.000000612	
H	0.000003933	0.000000000	-0.000004726	
H	0.000015669	0.000000000	0.000030511	
S	-0.000021108	0.000000000	0.000042392	
H	-0.000007197	0.000000000	0.000011658	

**Geom.No<br>5=-670.130875334 Ha**				
Atom	X	Y	Z	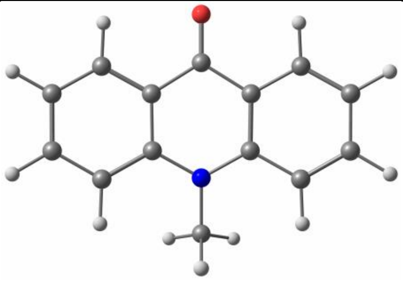
C	-0.000001670	-0.000000453	-0.000001193	
C	0.000008374	-0.000005822	-0.000001257	
C	0.000002869	0.000003676	-0.000003162	
C	-0.000004216	0.000000572	0.000006893	
C	-0.000005048	0.000000891	-0.000003333	
C	0.000004889	-0.000001754	-0.000002000	
N	-0.000012637	0.000017666	-0.000007600	
C	0.000006051	-0.000000082	0.000013394	
C	-0.000000121	0.000000061	-0.000000647	
C	0.000007382	-0.000003957	0.000003724	
C	0.000002584	0.000000596	-0.000005419	
C	-0.000003789	-0.000000740	-0.000003533	
C	0.000000399	0.000001187	0.000002142	
C	-0.000007344	-0.000001750	0.000004438	

O	-0.000006036	0.000000369	-0.000002764	
H	0.000004494	-0.000002110	-0.000007293	
H	0.000001227	-0.000000156	0.000000932	
H	0.000001465	0.000000453	0.000000462	
H	-0.000001085	0.000000180	0.000002749	
H	0.000001397	0.000000188	-0.000001976	
H	0.000001000	0.000000064	0.000001035	
H	0.000000113	0.000000643	0.000000580	
H	-0.000002946	-0.000001652	0.000004237	
C	0.000015838	-0.000020655	0.000006897	
H	-0.000003439	0.000010208	-0.000003254	
H	-0.000003390	0.000004209	-0.000004274	
H	-0.000006362	-0.000001834	0.000000222	

Geom.No
5*–S$_1$ = -669.953518683 Ha

Atom	X	Y	Z	
C	0.001468800	0.001306003	-0.014311619	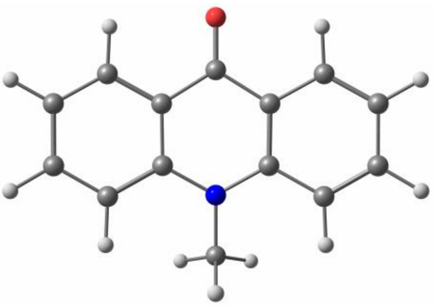
C	0.004149509	-0.000735113	0.018325511	
C	-0.007929979	0.000236431	0.003322763	
C	0.006366228	-0.000302096	-0.001943657	
C	0.002246243	-0.000461559	0.001194686	
C	-0.005244037	-0.001119324	-0.002114223	
C	0.002234971	0.002222086	0.001039014	
C	-0.001297352	-0.000930055	-0.008452168	
C	0.017660061	-0.003111007	-0.005658745	
N	-0.006147717	-0.003163889	-0.003149872	
C	0.001646542	0.000527929	0.006435157	
C	0.002147453	-0.000444212	0.001369963	
C	-0.004625234	-0.001228109	-0.003212401	
C	-0.011272831	0.003546751	0.008307965	
C	0.002077794	0.004103694	0.000763843	
O	-0.002882271	-0.000726284	-0.001551682	
H	0.000157316	0.000901467	0.000726183	
H	0.000013596	-0.000065916	0.000470644	
H	0.000088075	0.000455664	0.000087984	
H	-0.000411324	0.000239363	0.000014109	
H	-0.000182535	0.000199133	-0.000392060	
H	0.000158968	0.000443207	-0.000037869	
H	0.000401083	-0.000134057	-0.000217245	
H	0.000774354	0.000792950	-0.000369232	
H	-0.000473261	-0.000738933	0.000646528	
H	0.000239722	-0.000864334	-0.000619243	
H	-0.001364177	-0.000949789	-0.000674335	

Geom.No
5*–T_1= -670.036771603 Ha

Atom	X	Y	Z
C	0.000031763	-0.000004276	0.000001298
C	-0.000032977	0.000036818	-0.000001408
C	-0.000009151	-0.000035414	0.000003644
C	0.000010651	0.000010191	-0.000002532
C	-0.000004088	0.000020294	0.000000904
C	-0.000014282	-0.000033896	-0.000001649
C	-0.000000047	-0.000012189	0.000010570
C	0.000006692	-0.000030848	0.000005949
C	0.000034209	0.000039840	-0.000014051
N	0.000011567	-0.000023637	0.000007235
C	-0.000009968	0.000009827	-0.000000632
C	0.000003729	0.000017675	-0.000001404
C	0.000010766	-0.000032138	-0.000000229
C	-0.000051526	-0.000000693	0.000007657
C	0.000234381	0.000096483	0.000233334
O	-0.000000733	0.000034594	-0.000007049
H	-0.000010537	-0.000009488	-0.000005728
H	0.000001303	0.000000604	-0.000000975
H	0.000000837	-0.000005251	0.000000877
H	-0.000008965	-0.000000708	-0.000000433
H	0.000008256	-0.000000499	-0.000000331
H	-0.000000869	-0.000005464	0.000001639
H	-0.000002009	0.000001781	-0.000000604
H	-0.000003426	0.000004825	0.000003783
H	-0.000236222	-0.000075194	-0.000248065
H	-0.000014891	-0.000010629	-0.000018703
H	0.000045538	0.000007393	0.000026903

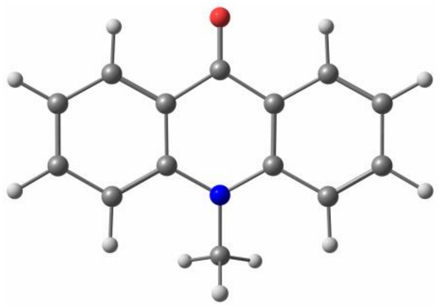

Geom.No
6 =–1413.88738546 Ha

Atom	X	Y	Z
C	-0.000000218	0.000000835	-0.000000316
C	0.000002711	0.000001939	0.000000233
C	0.000000657	0.000001222	-0.000000643
C	0.000000961	0.000000192	-0.000000360
C	-0.000000477	0.000000474	-0.000000692
C	-0.000000935	0.000001068	-0.000000389
H	0.000002102	0.000001477	-0.000000821
H	0.000001418	0.000001133	-0.000000843
H	0.000000261	0.000000164	-0.000000307
H	-0.000000769	-0.000000779	0.000000236
C	-0.000000520	0.000003589	-0.000000687
C	0.000000099	0.000004150	0.000001993
C	0.000001405	0.000000981	0.000000289
N	0.000002309	0.000001689	-0.000002028
C	0.000001296	0.000000296	-0.000001486
C	-0.000000162	0.000004161	0.000000287
C	-0.000001670	0.000001118	-0.000000216

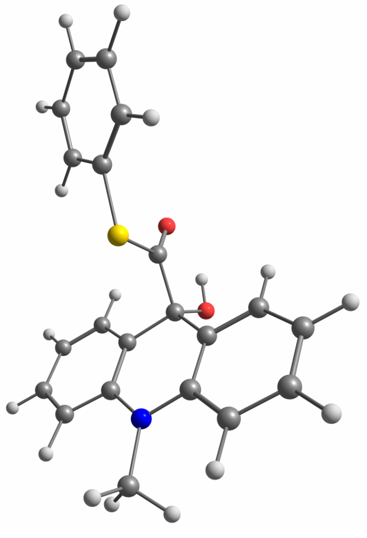

C	0.000000325	0.000000179	0.000000339
C	0.000000003	0.000002822	0.000000630
C	-0.000000507	0.000001906	-0.000001993
H	-0.000000418	-0.000000280	0.000001354
H	-0.000001030	0.000001431	0.000000660
H	0.000000338	0.000002253	-0.000000104
H	0.000001274	0.000002126	-0.000000911
H	0.000001246	0.000003234	-0.000001228
H	0.000002060	0.000001979	-0.000000975
H	0.000001806	0.000002825	-0.000000730
S	-0.000004044	-0.000002698	0.000003055
O	-0.000002092	0.000000395	0.000001028
C	0.000001593	-0.000003486	-0.000001056
C	-0.000000243	-0.000003109	0.000000290
C	-0.000000516	-0.000003580	-0.000000041
C	-0.000000724	-0.000004620	0.000000184
C	-0.000000930	-0.000003532	0.000000429
C	-0.000000314	-0.000002288	0.000000633
H	0.000000214	-0.000003243	-0.000000170
H	-0.000000174	-0.000004662	-0.000000103
H	-0.000000931	-0.000005000	0.000000441
H	-0.000001340	-0.000003594	0.000000686
H	-0.000000788	-0.000002248	0.000000333
O	-0.000001442	-0.000001449	0.000001527
H	-0.000001831	0.000000929	0.000001472

Geom.No
7 = -1413.30814707 Ha

Atom	X	Y	Z	
C	-0.000001673	0.000011571	0.000014517	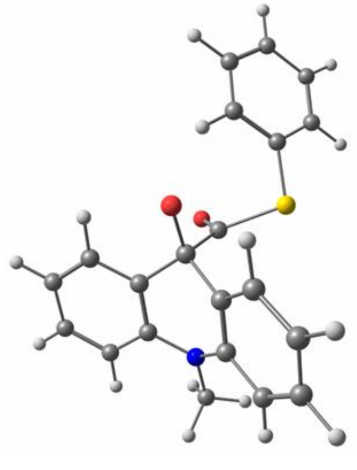
C	-0.000027109	0.000008782	0.000024874	
C	-0.000014545	0.000005282	0.000014447	
C	0.000004553	0.000003355	-0.000002373	
C	-0.000002235	0.000003608	0.000005216	
C	0.000003625	0.000001661	-0.000002317	
S	0.000030777	-0.000018630	-0.000041175	
C	-0.000003812	-0.000025243	-0.000016172	
O	-0.000003776	0.000043364	0.000013533	
H	-0.000001348	0.000000884	-0.000011652	
C	0.000008288	-0.000024319	0.000021536	
C	0.000002548	0.000003574	-0.000000581	
C	-0.000002220	0.000000592	0.000002097	
N	0.000003033	-0.000002339	0.000003385	
C	-0.000003787	-0.000004146	-0.000004196	
C	0.000005026	0.000004413	-0.000000177	
C	-0.000000956	-0.000002659	-0.000001560	
C	-0.000003064	-0.000000199	0.000002466	
C	-0.000001534	0.000000876	-0.000002757	
C	-0.000000805	-0.000004449	-0.000001400	
C	0.000001174	-0.000001934	0.000000134	
C	0.000001856	-0.000003199	0.000000160	

C	0.000001032	0.000000001	0.000000347
C	0.000000255	-0.000004488	-0.000001428
O	0.000005607	0.000009381	-0.000011815
C	-0.000000428	0.000000192	-0.000000559
H	-0.000002756	-0.000000356	-0.000000746
H	-0.000001916	-0.000000150	-0.000001150
H	-0.000002620	-0.000001039	-0.000000602
H	-0.000001569	-0.000001435	-0.000000328
H	0.000000971	-0.000001889	-0.000000829
H	0.000000969	-0.000000472	0.000000502
H	0.000002479	-0.000002000	-0.000000017
H	0.000002231	-0.000002639	-0.000000164
H	0.000001254	-0.000000864	0.000000087
H	0.000000072	-0.000000630	0.000000281
H	-0.000000032	-0.000001939	-0.000000243
H	-0.000000824	0.000004670	0.000001344
H	0.000000141	0.000003031	-0.000000727
H	0.000001120	0.000001305	0.000000781
H	-0.000000002	-0.000001523	-0.000002741

Geom.No
VIII-TS.

Atom	X	Y	Z
C	-0.000000188	0.000002811	-0.000001865
C	0.000004359	-0.000000004	0.000002820
C	-0.000001200	0.000000607	0.000001121
C	0.000000943	0.000001093	-0.000002201
C	-0.000000316	0.000002864	-0.000000364
C	-0.000001609	0.000004094	-0.000001265
C	0.000002669	-0.000004405	-0.000003726
C	0.000005846	0.000000113	-0.000002078
C	0.000002248	0.000000189	-0.000004387
N	0.000000458	-0.000004277	-0.000002644
C	0.000000419	-0.000004797	-0.000002071
C	0.000001386	-0.000003936	-0.000004155
C	-0.000000517	-0.000002650	-0.000004281
C	-0.000000648	-0.000003145	-0.000002630
C	0.000007643	0.000012635	-0.000002217
O	0.000004237	-0.000005200	0.000002528
O	-0.000016286	0.000001237	-0.000007837
C	-0.000002535	-0.000000846	-0.000002133
S	-0.000008164	-0.000003874	0.000009965
C	-0.000001329	0.000002161	0.000003175
C	-0.000000381	0.000001822	0.000002806
C	0.000000264	0.000002987	0.000004605
C	0.000000286	0.000002363	0.000005472
C	0.000000411	0.000000752	0.000006060
C	0.000001043	-0.000000253	0.000005151
H	0.000000882	-0.000001848	0.000005492
H	0.000000139	-0.000003873	-0.000004368
H	-0.000000026	-0.000000332	-0.000003084

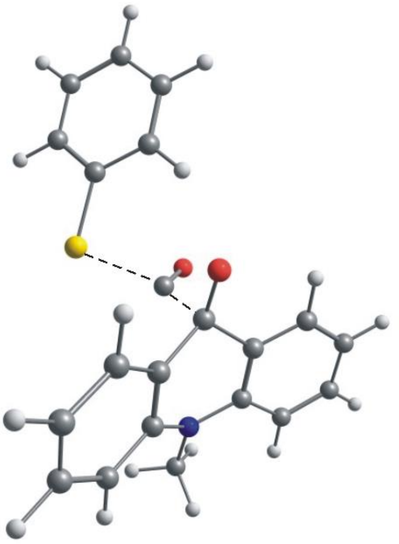

H	0.000000437	-0.000005755	-0.000004629
H	0.000000323	-0.000004247	-0.000003223
H	-0.000000023	0.000000389	-0.000001607
H	-0.000000902	0.000003879	-0.000000860
H	-0.000001020	0.000005514	-0.000001081
H	-0.000000720	0.000003524	-0.000001472
H	0.000000321	-0.000002176	-0.000000857
H	0.000000339	-0.000004095	-0.000001124
H	0.000000351	-0.000002905	-0.000002716
H	0.000000915	0.000000325	0.000007499
H	0.000000240	0.000003312	0.000006751
H	-0.000000152	0.000004447	0.000003830
H	-0.000000144	0.000001500	0.000001601

Geom.No
8 = -935.003763764 Ha

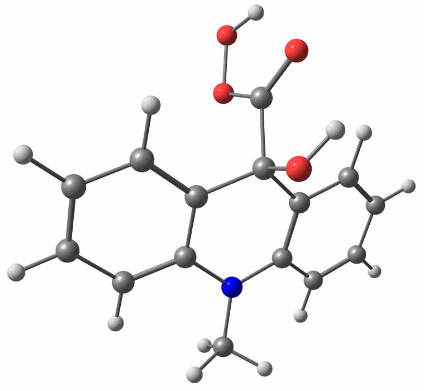

Atom	X	Y	Z
C	-0.000011816	-0.000000055	0.000006695
C	-0.000001296	0.000001799	-0.000005911
C	0.000001697	0.000003643	0.000000960
C	-0.000003509	0.000004590	0.000003896
C	-0.000004051	0.000002330	0.000000919
C	0.000012610	-0.000004624	-0.000011272
H	-0.000000780	0.000000920	0.000000441
H	-0.000003545	0.000003169	-0.000000018
H	-0.000001036	0.000005354	0.000000397
H	0.000000274	0.000003163	0.000000536
C	0.000001520	0.000009386	0.000007886
C	-0.000001172	-0.000004289	0.000001156
C	0.000002923	0.000000690	0.000001562
N	0.000004470	0.000000468	-0.000000024
C	-0.000000565	-0.000011272	-0.000003795
C	-0.000005465	0.000000412	-0.000000619
C	-0.000000927	-0.000001821	-0.000004717
C	0.000004641	-0.000003592	-0.000000118
C	-0.000000077	-0.000003952	0.000001369
C	-0.000001306	-0.000002856	-0.000004454
H	0.000002667	-0.000001908	0.000002584
H	0.000001472	-0.000003911	-0.000000527
H	0.000000899	-0.000004825	-0.000002191
H	-0.000000580	-0.000003445	-0.000002943
H	-0.000002232	-0.000001361	-0.000002680
H	-0.000000279	-0.000002631	-0.000003116
H	0.000000207	-0.000003881	-0.000000705
O	0.000001740	0.000001780	0.000000210
H	0.000001324	-0.000000014	0.000003050
O	0.000002002	0.000007228	0.000002219
O	-0.000004766	0.000001095	0.000004190
H	0.000002681	0.000000204	-0.000001820
O	0.000002275	0.000008205	0.000006842

Geom.No XI-TS.				
Atom	X	Y	Z	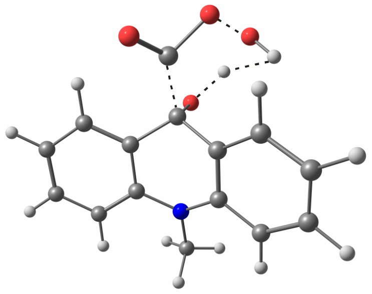
C	-0.000000393	0.000000061	-0.000001301	
C	-0.000009261	-0.000006492	-0.000009743	
C	-0.000020159	-0.000020949	0.000029853	
C	0.000002833	0.000011480	-0.000011960	
C	0.000008803	-0.000004319	-0.000000674	
N	-0.000002642	-0.000002612	0.000006807	
C	0.000010156	0.000021830	-0.000014382	
C	0.000004439	-0.000012396	0.000017802	
C	0.000006194	-0.000006840	-0.000004570	
C	0.037752729	-0.010613958	0.016223306	
C	-0.000009805	-0.000003220	0.000017682	
C	0.000007399	0.000005947	-0.000004797	
C	0.000002793	-0.000000742	-0.000001355	
C	-0.000008021	-0.000000193	-0.000002468	
O	0.066534301	0.030771640	-0.017672502	
C	-0.037746893	0.010725529	-0.016272031	
O	-0.000070513	0.000021237	-0.000030949	
O	-0.000008667	0.000018909	0.000065223	
C	-0.000000771	-0.000007573	-0.000001219	
O	0.000009894	-0.000036501	0.000030209	
H	0.000001325	-0.000001663	0.000006532	
H	-0.000000183	0.000001885	0.000002085	
H	-0.000001523	0.000001716	0.000001194	
H	-0.000001182	0.000004167	-0.000003993	
H	0.000000902	-0.000004177	0.000006900	
H	-0.000001527	-0.000000107	0.000000810	
H	-0.000001441	-0.000002085	-0.000000646	
H	0.000002540	-0.000002983	-0.000002224	
H	-0.000002208	-0.000004773	0.000000559	
H	-0.000000011	0.000002981	-0.000003092	
H	0.000000068	-0.000003665	0.000002148	
H	-0.066453197	-0.030839426	0.017658142	
H	-0.000005979	-0.000012708	-0.000031343	

Geom.No<br>9= -783.845241706 Ha			
Atom	X	Y	Z
C	-0.000003697	0.000002446	-0.000014793
C	0.000002314	-0.000000933	0.000008333
C	-0.000001217	-0.000003198	0.000002700
C	0.000004336	0.000000328	0.000001581
C	0.000004926	-0.000001305	0.000003524
C	-0.000007755	-0.000001029	-0.000007555
N	0.000007393	0.000000704	0.000000979
C	-0.000005213	-0.000002967	-0.000001330
C	0.000000330	0.000003971	0.000003834
C	-0.000011000	-0.000008618	-0.000007880
C	-0.000002247	0.000003865	-0.000007202
C	-0.000002250	0.000001186	0.000006776
C	0.000003876	0.000000304	0.000005644
C	0.000009818	0.000004769	-0.000005549
C	-0.000012508	0.000004987	0.000019564
O	0.000003593	0.000002240	0.000001128
C	-0.000008468	-0.000005079	-0.000006976
O	-0.000006529	-0.000007501	-0.000010075
H	0.000001778	0.000001712	0.000001095
H	-0.000000735	0.000001005	0.000000100
H	0.000002823	0.000000808	0.000000856
H	0.000009003	0.000001827	-0.000001928
H	0.000001635	0.000000603	0.000003297
H	0.000001306	-0.000000319	0.000001102
H	-0.000001737	-0.000001334	-0.000000322
H	-0.000001556	-0.000001767	-0.000002097
H	0.000002183	0.000002078	-0.000006761
H	-0.000000947	-0.000001576	0.000003175
H	0.000002443	0.000002699	-0.000000949
H	0.000008102	0.000000095	0.000009727

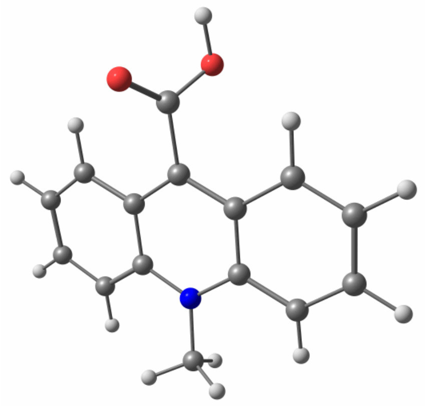

R_2=OCH_3, R_4=H, R_6=H, $R_{2'}$=H				
**Geom.No<br>1 =-1452.38473080 Ha**				
Atom	X	Y	Z	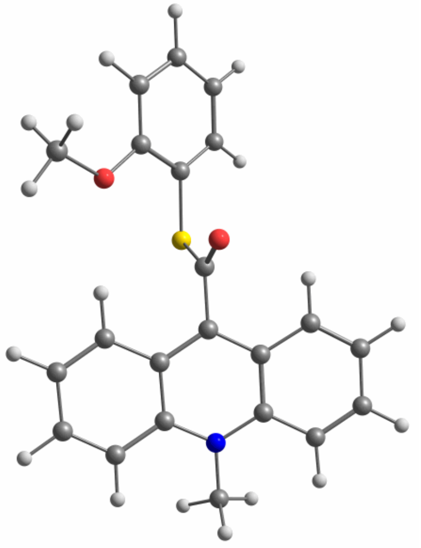
C	-0.000024540	-0.000016557	0.000002846	
C	-0.000009473	0.000004615	-0.000004909	
C	0.000013116	0.000003547	-0.000002309	
C	-0.000006419	-0.000002262	0.000006811	
C	-0.000003536	0.000002151	-0.000015756	
C	0.000014992	0.000004608	0.000001497	
S	0.000009109	0.000015219	-0.000004765	
C	-0.000008617	-0.000034747	-0.000005876	
O	0.000006327	0.000009540	0.000004884	
C	-0.000020918	0.000031864	-0.000022584	
C	0.000012682	-0.000012334	0.000013550	
C	-0.000002387	-0.000001549	0.000011383	
N	-0.000003348	0.000005175	-0.000003412	
C	0.000008201	-0.000006411	-0.000001109	
C	0.000012672	-0.000009094	0.000002754	
C	-0.000005943	-0.000000019	0.000001690	
C	0.000001260	0.000002081	-0.000002776	
C	-0.000002322	-0.000001999	0.000003238	
C	0.000002947	0.000001407	-0.000004064	
C	0.000001503	0.000002802	0.000000687	
C	0.000003084	-0.000002599	0.000002134	
C	-0.000001277	0.000000404	0.000000441	
C	-0.000001535	0.000000617	0.000001403	
C	0.000000976	-0.000001886	0.000003995	
H	0.000000410	-0.000000859	-0.000000345	
H	-0.000000947	-0.000001952	0.000003246	
H	-0.000000366	-0.000002077	0.000001054	
H	-0.000000905	-0.000000069	0.000000039	
H	0.000001444	-0.000001963	-0.000000417	
H	0.000003927	0.000001373	-0.000001633	
H	0.000003422	0.000000347	0.000000482	
H	0.000000303	0.000001249	0.000001782	
H	0.000002079	-0.000006218	0.000003665	
H	0.000003277	-0.000002118	0.000003142	
H	0.000005850	-0.000001040	0.000005472	
H	-0.000000672	0.000001599	-0.000002741	

H	-0.000000977	0.000001460	-0.000002638
H	-0.000004085	0.000003772	-0.000003750
H	-0.000003964	0.000002470	-0.000002028
O	0.000001592	0.000012352	-0.000010725
C	0.000003476	-0.000006020	0.000023703
H	-0.000001482	-0.000000525	-0.000003394
H	-0.000006599	0.000000688	-0.000002639
H	-0.000002338	0.000002960	-0.000002027

Geom.No
2= -1603.54078905 Ha

Atom	X	Y	Z
C	0.000003439	-0.000003421	-0.000001403
C	0.000007086	-0.000003088	-0.000004246
C	-0.000005368	-0.000003032	0.000010069
C	0.000002881	-0.000003766	-0.000000956
C	0.000002064	-0.000004828	0.000004144
C	0.000000603	0.000000841	0.000000362
C	-0.000004923	-0.000009587	0.000002566
C	0.000005622	0.000003023	0.000003885
C	-0.000006241	-0.000003683	-0.000007908
N	0.000007282	0.000008048	-0.000003971
C	-0.000003226	0.000002764	0.000006314
C	0.000001669	0.000001851	-0.000000035
C	-0.000000069	0.000001189	-0.000005723
C	-0.000004043	0.000002992	0.000002124
C	0.000002211	0.000011527	0.000004952
O	-0.000000360	-0.000001355	-0.000005826
O	-0.000004977	0.000002108	-0.000003854
O	0.000010314	0.000004394	-0.000002111
C	-0.000002863	-0.000005329	0.000001139
S	0.000001952	-0.000001856	-0.000004753
C	-0.000014241	-0.000002698	0.000016651
C	0.000009354	0.000013762	-0.000005890
C	-0.000004502	-0.000000606	0.000002766
C	-0.000000898	0.000002495	-0.000004777
C	-0.000003012	0.000001047	0.000002765
C	-0.000000456	0.000001732	-0.000011568
H	0.000000452	-0.000002189	0.000003452
H	0.000001742	-0.000003192	0.000001467
H	0.000003461	-0.000004512	0.000000648
H	0.000001309	0.000001802	0.000000022
H	0.000001204	0.000001642	-0.000002586
H	-0.000000708	0.000003297	-0.000001719
H	-0.000002747	0.000001601	-0.000000881
H	-0.000001891	0.000000493	-0.000000221
H	0.000000288	-0.000001569	0.000001670
H	-0.000000758	-0.000001755	0.000001763
H	-0.000001128	-0.000000644	0.000001209

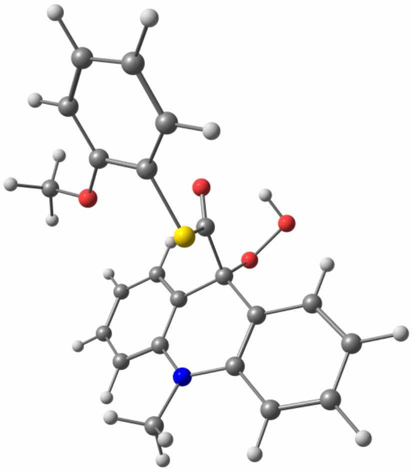

O	0.000003092	-0.000011805	0.000002978
H	0.000000499	-0.000002144	0.000000633
H	-0.000001260	0.000001224	-0.000000685
H	-0.000003042	0.000004930	-0.000001254
H	-0.000001134	0.000001170	0.000001650
H	0.000002284	-0.000000513	-0.000006798
C	-0.000005837	-0.000001321	-0.000002414
H	0.000001243	-0.000001096	-0.000001257
H	0.000001850	0.000001689	0.000003962
H	0.000001781	-0.000001633	0.000003645

Geom.No
4= -744.403700456 Ha

Atom	X	Y	Z
C	-0.000077524	0.000000000	-0.000082393
C	-0.000097055	0.000000000	-0.000018789
C	0.000114717	0.000000000	0.000006667
C	-0.000082441	0.000000000	0.000078147
C	-0.000047342	-0.000000000	-0.000085946
C	0.000199964	-0.000000000	0.000113931
H	0.000044865	-0.000000000	0.000028725
H	-0.000012032	-0.000000000	-0.000000998
H	0.000022320	0.000000000	-0.000015395
H	0.000007623	-0.000000000	0.000026424
O	-0.000118585	0.000000000	-0.000071098
C	0.000091589	-0.000000000	0.000033946
H	-0.000030818	-0.000000000	0.000013472
H	-0.000003050	0.000005254	-0.000011278
H	-0.000003050	-0.000005254	-0.000011278
S	-0.000009183	0.000000000	-0.000004137

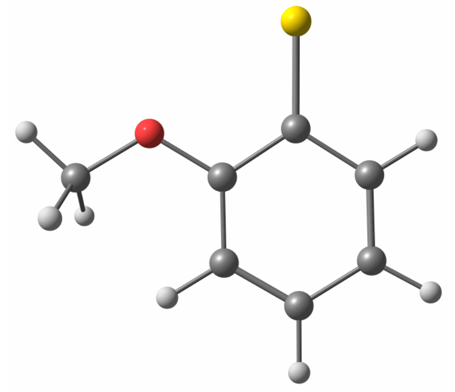

Geom.No
6= -1528.41187944 Ha

Atom	X	Y	Z
C	-0.000009300	0.000003948	0.000001894
C	0.000006824	0.000002705	0.000002157
C	0.000000863	0.000001456	-0.000003866
C	-0.000001550	0.000003264	0.000000710
C	-0.000003134	0.000001880	-0.000004465
C	0.000003354	0.000000346	-0.000001756
S	-0.000003357	0.000004261	-0.000001267
C	0.000001309	-0.000004207	-0.000000525
O	0.000001074	0.000003070	0.000000581
C	0.000001079	-0.000001140	-0.000000628
C	0.000001399	0.000002711	0.000001473
C	0.000000874	-0.000000447	-0.000000728
N	0.000000909	-0.000000368	-0.000001904
C	-0.000002421	-0.000001179	0.000002446
C	0.000000792	-0.000002814	0.000001195
C	0.000002451	0.000000700	0.000002403

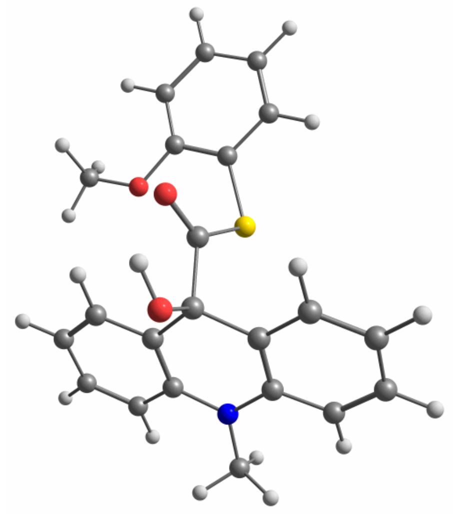

C	0.000002329	0.000000530	-0.000000785	
C	0.000000419	0.000002044	-0.000000029	
C	0.000000173	0.000000539	0.000001036	
C	-0.000000340	-0.000003066	0.000000802	
C	-0.000000275	-0.000002715	0.000000027	
C	-0.000000945	-0.000001778	-0.000000094	
C	0.000000852	-0.000002022	0.000000904	
O	-0.000002000	0.000000381	-0.000001004	
C	-0.000000125	-0.000000264	0.000001277	
H	0.000000304	-0.000000161	0.000000462	
H	0.000001180	0.000001465	0.000000200	
H	0.000002007	0.000002510	0.000000591	
H	0.000001499	0.000001192	0.000000124	
H	0.000000588	-0.000001594	0.000001249	
H	-0.000000304	-0.000003040	0.000000795	
H	-0.000001610	-0.000003300	0.000000886	
H	-0.000000995	-0.000002587	-0.000000532	
H	-0.000000344	-0.000001948	0.000000865	
H	-0.000000400	-0.000001876	0.000000184	
H	0.000000130	-0.000002160	0.000001790	
H	-0.000000430	0.000003229	-0.000001048	
H	-0.000000397	0.000003138	-0.000002309	
H	-0.000000426	0.000002694	-0.000000657	
H	-0.000001887	0.000001672	-0.000000873	
O	0.000001333	-0.000006364	-0.000004920	
H	0.000003357	-0.000000359	0.000001614	
C	-0.000000794	0.000003257	0.000004195	
H	-0.000001139	-0.000001018	-0.000000496	
H	-0.000001125	-0.000001423	-0.000000990	
H	-0.000001803	-0.000001162	-0.000000983	

**Geom.No 7= -1527.82834549 Ha**				
Atom	X	Y	Z	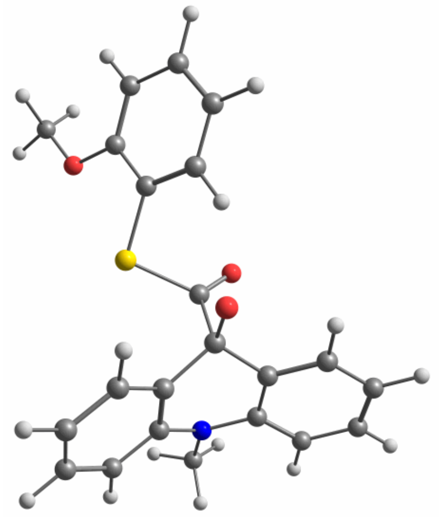
C	0.000000289	-0.000000065	-0.000001086	
C	0.000006480	0.000002773	0.000001457	
C	-0.000001755	-0.000000921	-0.000002175	
C	0.000000147	0.000000188	-0.000001609	
C	0.000000812	-0.000000151	0.000001188	
C	-0.000001930	0.000000076	-0.000000389	
C	-0.000028712	-0.000022825	-0.000022647	
C	0.000002832	0.000002678	0.000003038	
C	-0.000001149	-0.000000850	-0.000001716	
N	0.000000595	0.000002017	0.000000299	
C	-0.000000565	-0.000001611	0.000000640	
C	0.000000832	-0.000000262	-0.000000180	
C	-0.000001392	0.000000207	-0.000001656	
C	-0.000000574	-0.000000774	0.000000857	
C	0.000007925	0.000024193	0.000000970	
O	-0.000002355	-0.000006478	0.000004173	
O	0.000019095	0.000001229	0.000013168	

C	0.000000547	-0.000000475	-0.000000836	
S	-0.000002077	-0.000000943	0.000001619	
C	-0.000002783	0.000004895	0.000002944	
C	-0.000004762	0.000002357	-0.000005562	
C	0.000006186	-0.000004760	0.000001830	
C	-0.000003135	0.000005583	0.000006687	
C	-0.000004175	-0.000000704	-0.000009482	
C	0.000009337	-0.000004617	0.000009453	
O	-0.000006213	0.000003190	0.000001921	
H	-0.000000182	-0.000000387	-0.000000198	
H	-0.000000950	0.000000416	-0.000000388	
H	-0.000000273	-0.000000587	-0.000000615	
H	-0.000000677	-0.000000583	-0.000000437	
H	-0.000000172	-0.000000411	-0.000000315	
H	-0.000000477	0.000000483	-0.000000195	
H	-0.000000017	0.000000483	-0.000000278	
H	-0.000000321	0.000000014	-0.000000411	
H	0.000000039	-0.000000877	-0.000000275	
H	0.000000223	-0.000000189	-0.000000306	
H	-0.000000305	-0.000000690	-0.000000179	
H	0.000001167	0.000001145	0.000001677	
H	0.000000955	0.000000734	0.000000055	
H	-0.000000681	0.000000940	0.000000263	
H	0.000001921	0.000000232	0.000001203	
C	0.000004653	-0.000004577	-0.000005473	
H	0.000000689	-0.000000255	0.000000135	
H	0.000000348	0.000000086	0.000000975	
H	0.000000560	0.000000070	0.000001852	

**Geom.No<br>VIII-TS= -1527.82296751 Ha**				
Atom	X	Y	Z	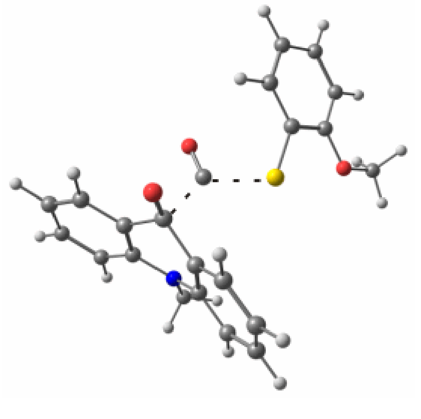
C	-0.000002454	0.000001144	0.000000134	
C	0.000000491	-0.000001682	-0.000002126	
C	-0.000000965	-0.000001746	-0.000003369	
C	-0.000002379	-0.000001236	-0.000002306	
C	-0.000002581	0.000000452	-0.000002231	
C	-0.000002152	0.000002125	-0.000001349	
S	-0.000001815	0.000001326	-0.000002881	
C	-0.000003522	0.000002138	-0.000000505	
O	0.000000972	-0.000002737	-0.000000809	
O	-0.000002683	0.000004642	-0.000000413	
C	-0.000002954	0.000003747	-0.000000620	
C	-0.000000478	-0.000003737	0.000005815	
C	0.000003534	-0.000001867	-0.000001461	
C	0.000002206	-0.000000655	0.000001163	
N	0.000002704	0.000000083	-0.000000061	
C	-0.000000218	0.000000401	0.000001350	
C	0.000000072	-0.000001176	-0.000001869	
C	0.000003803	-0.000002048	0.000002266	
C	0.000004372	-0.000003078	0.000002151	

C	0.000004071	-0.000004443	0.000000875	
C	0.000002650	-0.000004191	0.000001044	
C	0.000000023	0.000001839	0.000001037	
C	-0.000001342	0.000001521	-0.000000061	
C	-0.000001966	0.000000755	-0.000001226	
C	-0.000001190	-0.000000422	-0.000001099	
O	0.000000488	-0.000003097	-0.000001519	
C	0.000002192	0.000001353	0.000003650	
H	-0.000002798	0.000000946	-0.000001889	
H	-0.000001690	-0.000001273	-0.000002695	
H	-0.000001748	0.000002800	0.000000209	
H	0.000000360	0.000002507	0.000001650	
H	0.000004375	-0.000000927	0.000003363	
H	0.000002175	-0.000004583	-0.000001062	
H	0.000004571	-0.000005490	0.000000974	
H	0.000005648	-0.000003503	0.000002966	
H	0.000002752	0.000001112	0.000003410	
H	0.000001676	0.000002541	0.000002608	
H	0.000002573	0.000001097	0.000003185	
H	-0.000003156	0.000002885	-0.000000912	
H	-0.000002447	0.000000579	-0.000001995	
H	-0.000001660	-0.000001798	-0.000002587	
H	-0.000001708	-0.000000956	-0.000002613	
H	-0.000003363	0.000005153	0.000000029	
H	-0.000002688	0.000004602	0.000000578	
H	-0.000003751	0.000004896	-0.000000798	

R_2=H, R_4= NO_2 R_6=H $R_{2'}$=H				
**Geom.No<br>1=-1542.34610948 Ha**				
Atom	X	Y	Z	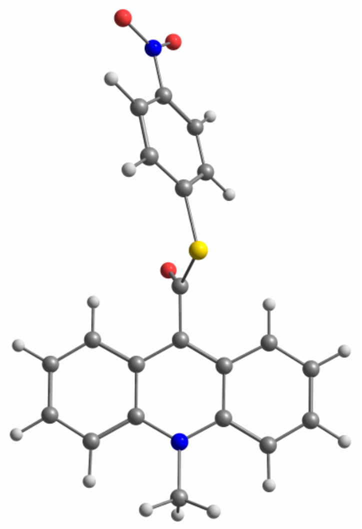
C	0.000000222	-0.000005670	-0.000010069	
C	0.000005789	0.000001850	0.000002651	
C	0.000005558	-0.000000324	0.000000149	
C	-0.000007683	-0.000009180	-0.000008521	
C	-0.000020287	-0.000002303	0.000004942	
C	-0.000002699	0.000005418	0.000009166	
S	-0.000003077	0.000006642	-0.000002458	
C	-0.000007132	-0.000014429	0.000007558	
O	0.000001638	0.000003516	-0.000000306	
C	0.000003437	0.000004427	-0.000006131	
C	0.000000636	0.000002224	0.000002033	
C	-0.000002298	0.000002141	0.000003899	
N	-0.000000611	0.000001891	-0.000002650	
C	0.000000149	0.000002156	0.000000836	
C	0.000006603	-0.000001119	0.000001238	
C	-0.000001005	-0.000002139	-0.000001620	
C	-0.000001763	-0.000000041	0.000001923	
C	-0.000000845	0.000000645	0.000000971	
C	0.000000551	0.000000854	-0.000001083	
C	0.000003241	-0.000000682	0.000004925	

C	0.000000383	0.000001454	0.000001826	
C	-0.000001480	0.000002009	-0.000000231	
C	-0.000001830	-0.000000682	0.000001012	
C	-0.000001851	0.000002947	0.000000222	
H	-0.000000114	0.000001093	0.000001092	
H	-0.000000490	0.000000078	0.000001117	
H	-0.000000142	-0.000000722	0.000000446	
H	-0.000001672	-0.000000698	0.000000385	
H	0.000000330	0.000000999	0.000000014	
H	0.000000945	0.000001145	-0.000000186	
H	0.000001183	0.000002066	0.000000998	
H	0.000003356	0.000001786	0.000000629	
H	0.000000192	0.000000278	0.000002045	
H	0.000000581	0.000001712	0.000002643	
H	-0.000001534	0.000004681	0.000000023	
H	0.000002504	-0.000000125	-0.000003064	
H	-0.000001566	0.000000267	0.000001838	
N	0.000040498	-0.000003280	-0.000015698	
H	-0.000001899	-0.000004351	-0.000003866	
H	0.000001774	-0.000002003	-0.000000011	
O	-0.000011805	-0.000001915	0.000003888	
O	-0.000007786	-0.000002617	-0.000002578	

**Geom.No 2=-1693.51451378 Ha**				
Atom	X	Y	Z	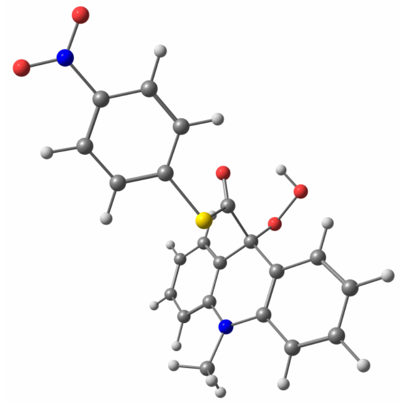
C	0.000000437	0.000002030	0.000001399	
C	-0.000001054	0.000001277	-0.000000436	
C	-0.000001294	-0.000002474	-0.000000170	
C	0.000001348	0.000000919	-0.000002212	
C	0.000000408	0.000000694	-0.000000144	
C	-0.000001999	-0.000000317	0.000000545	
C	-0.000006050	0.000003146	0.000001834	
C	0.000003846	-0.000000525	0.000002413	
C	0.000000311	-0.000001407	-0.000001928	
N	-0.000001250	0.000001658	0.000004230	
C	0.000002757	-0.000002517	-0.000002977	
C	0.000003540	-0.000001677	0.000001614	
C	0.000000247	-0.000001313	0.000000003	
C	0.000001989	-0.000000424	-0.000001158	
C	-0.000000387	-0.000008110	-0.000002486	
O	0.000003708	0.000004351	0.000005189	
O	0.000003011	0.000001334	-0.000000383	
O	0.000002864	0.000000235	0.000001431	
C	0.000000215	-0.000000882	0.000002940	
S	-0.000001630	0.000002576	-0.000004799	
C	-0.000005671	0.000000820	-0.000006002	
C	0.000003080	0.000000858	0.000004088	
C	0.000001816	0.000001727	-0.000005348	
C	-0.000003360	-0.000010298	-0.000008898	
C	0.000001461	0.000007159	0.000002432	

C	-0.000008286	-0.000002822	0.000001462	
H	-0.000000618	-0.000000102	-0.000002155	
H	-0.000000482	0.000000166	-0.000000314	
H	-0.000000955	0.000001299	0.000000078	
H	-0.000001204	0.000001271	0.000000146	
H	0.000000415	-0.000000531	0.000001733	
H	0.000001419	-0.000001018	0.000001320	
H	0.000002241	-0.000002352	0.000000887	
H	0.000005094	-0.000003598	-0.000000026	
H	-0.000000824	-0.000002369	0.000001548	
H	0.000001160	-0.000002034	-0.000000689	
H	-0.000001206	-0.000000285	0.000002678	
H	-0.000002909	0.000000067	-0.000002024	
H	-0.000003741	-0.000001786	-0.000002241	
N	-0.000009197	-0.000018259	0.000000359	
H	-0.000001961	0.000000351	-0.000001531	
H	0.000001550	-0.000000178	0.000000816	
H	0.000003033	0.000000438	0.000002890	
O	0.000009856	0.000012302	0.000001707	
O	-0.000001727	0.000020600	0.000002178	

**Geom.No 4=-834.419684305 Ha**				
Atom	X	Y	Z	
C	0.000048163	-0.000000000	-0.000067563	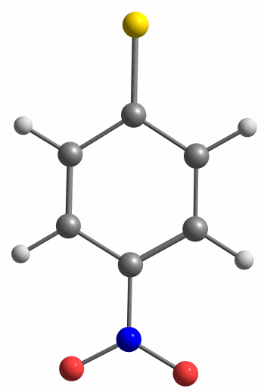
C	-0.000000018	0.000000000	0.000029121	
C	0.000007243	0.000000000	0.000003015	
C	0.000057919	0.000000000	0.000015703	
C	0.000003620	0.000000000	0.000024608	
C	-0.000081027	0.000000000	0.000017390	
H	0.000000726	0.000000000	0.000000802	
H	-0.000014509	0.000000000	-0.000000926	
N	-0.000028024	-0.000000000	-0.000016886	
H	0.000007317	0.000000000	0.000003904	
H	0.000001115	-0.000000000	-0.000003308	
S	-0.000019255	-0.000000000	-0.000010800	
O	0.000007622	-0.000000000	-0.000006854	
O	0.000009108	0.000000000	0.000011793	

**Geom.No 6= -1618.38613968 Ha**				
Atom	X	Y	Z	

C	0.000001527	-0.000001374	0.000001171	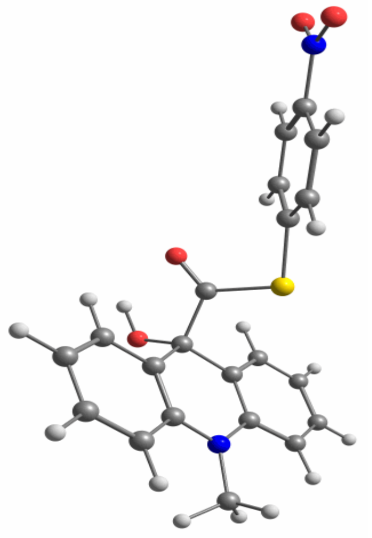
C	0.000001762	-0.000001170	0.000001287	
C	0.000000986	-0.000002428	0.000000353	
C	0.000000031	0.000000042	0.000003536	
C	0.000001578	-0.000000573	0.000001783	
C	0.000003184	-0.000001405	0.000001143	
C	-0.000001536	0.000006998	-0.000005429	
C	-0.000001728	0.000000100	0.000000311	
C	-0.000001505	-0.000000135	0.000000348	
N	-0.000000792	0.000003449	0.000002020	
C	-0.000002308	0.000001668	-0.000000519	
C	-0.000003044	0.000001606	-0.000000463	
C	-0.000002328	0.000001136	-0.000001301	
C	-0.000001237	0.000000622	-0.000001428	
C	0.000016537	-0.000022716	0.000007973	
O	-0.000010935	0.000003128	-0.000007339	
O	0.000003411	-0.000000357	0.000000243	
C	-0.000002789	-0.000000501	0.000001291	
S	-0.000002783	0.000013231	-0.000002664	
C	-0.000005659	-0.000007485	0.000003236	
C	0.000005873	0.000005917	-0.000004453	
C	-0.000001256	-0.000003040	-0.000001433	
C	0.000004496	0.000003802	-0.000000966	
C	0.000000652	-0.000000080	-0.000000625	
C	0.000004329	0.000002146	0.000002547	
H	0.000001743	-0.000002883	0.000000439	
H	-0.000002021	0.000001090	-0.000001076	
N	0.000000151	-0.000003026	-0.000001638	
O	-0.000000690	0.000000408	-0.000001981	
O	0.000000802	-0.000002364	-0.000001071	
H	-0.000002330	0.000001666	0.000001059	
H	-0.000003762	0.000002110	-0.000000429	
H	-0.000003116	0.000001768	-0.000002176	
H	-0.000001015	0.000000515	-0.000002237	
H	0.000002207	-0.000001075	-0.000000381	
H	0.000003090	-0.000001642	0.000001384	
H	0.000002219	-0.000001080	0.000003159	
H	0.000000649	0.000000250	0.000003273	
H	-0.000002280	0.000001041	0.000001929	
H	-0.000001329	0.000000959	0.000002642	
H	-0.000000701	0.000000885	0.000001644	
H	-0.000001349	-0.000000262	-0.000001115	
H	0.000002857	-0.000000951	-0.000001515	
H	-0.000001594	0.000000005	-0.000002530	

Geom.No 7=-1617.82381048 Ha				
Atom	X	Y	Z	

C	0.000005532	0.000002709	0.000004115	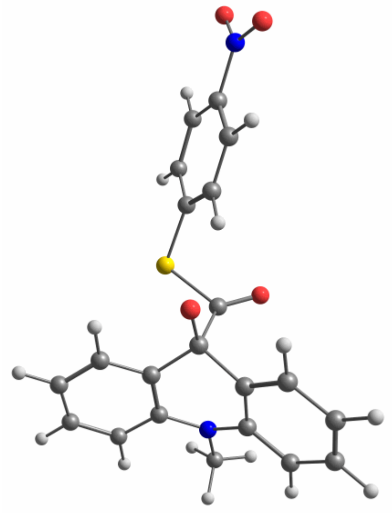
C	0.000002578	0.000012129	0.000004692	
C	0.000001559	0.000002045	0.000001592	
C	0.000002392	0.000004359	0.000002114	
C	0.000006586	0.000006290	0.000006889	
C	0.000002060	0.000006888	0.000001288	
C	-0.000045599	-0.000075026	-0.000068271	
C	0.000001865	0.000006255	0.000006932	
C	-0.000003246	-0.000000254	-0.000000831	
N	-0.000000415	0.000005013	0.000002273	
C	-0.000002624	-0.000003199	0.000001868	
C	-0.000001313	-0.000002908	-0.000001850	
C	-0.000002719	-0.000002631	-0.000004778	
C	0.000001863	0.000000017	-0.000006643	
C	-0.000017062	0.000060468	0.000047866	
O	0.000006292	-0.000001458	-0.000003447	
O	0.000047750	0.000001682	0.000029005	
C	-0.000000047	0.000001262	0.000004505	
S	0.000001563	0.000001514	-0.000017259	
C	0.000007557	0.000002598	-0.000005698	
C	-0.000005814	0.000004962	-0.000001699	
C	0.000006594	-0.000013819	-0.000015659	
C	-0.000003028	0.000000961	0.000004873	
C	-0.000013341	0.000007223	0.000006855	
C	0.000002708	-0.000014677	0.000000263	
H	-0.000004476	-0.000009581	0.000002794	
H	-0.000000951	-0.000005360	-0.000005457	
H	-0.000000647	-0.000002574	-0.000003341	
H	-0.000003116	-0.000004371	-0.000003096	
H	-0.000002828	-0.000001167	0.000000472	
H	0.000001906	0.000005610	0.000005965	
H	0.000006146	0.000004446	-0.000002069	
H	0.000007139	0.000006835	0.000002638	
H	0.000005004	0.000007890	0.000005406	
H	-0.000002243	0.000002792	0.000006516	
H	-0.000003031	0.000000425	0.000004191	
H	-0.000001178	0.000001597	0.000004515	
H	-0.000007787	-0.000006016	-0.000011566	
N	0.000009598	-0.000010274	-0.000002402	
H	-0.000002837	0.000009248	0.000000403	
H	0.000004726	-0.000010900	0.000003780	
O	-0.000010029	0.000012937	0.000014343	
O	0.000002915	-0.000013937	-0.000022084	

**Geom.No<br>VIII-TS= -1617.82071294 Ha**				
Atom	X	Y	Z	

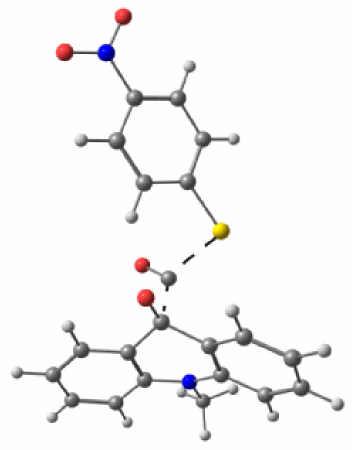

C	0.000004906	-0.000007499	-0.000000753
C	-0.000005837	0.000017288	-0.000000229
C	-0.000003422	-0.000000153	0.000004746
C	0.000003343	0.000003376	0.000004467
C	0.000003139	0.000003443	0.000001614
C	0.000001443	0.000000570	0.000001928
C	0.000002847	-0.000011317	0.000006682
C	0.000007843	-0.000008067	0.000009928
C	-0.000002820	-0.000001883	-0.000000487
N	0.000003921	0.000004144	0.000003415
C	0.000002881	0.000001763	0.000001947
C	0.000008141	0.000002337	-0.000019040
C	-0.000011499	0.000000723	0.000003634
C	-0.000006851	0.000000536	-0.000004872
O	-0.000000682	-0.000003164	-0.000002983
C	-0.000000871	0.000002713	-0.000000541
C	0.000003913	0.000004439	0.000001384
O	-0.000006424	0.000002066	-0.000001412
S	-0.000005847	0.000002487	-0.000001970
C	-0.000006233	0.000005344	-0.000001440
C	-0.000002868	0.000002316	0.000001597
C	0.000000706	-0.000003885	-0.000000888
C	0.000004592	-0.000004698	-0.000000169
C	0.000004334	-0.000003599	0.000001546
C	0.000003388	-0.000000529	0.000000335
N	0.000005247	0.000000698	-0.000005297
O	0.000000645	-0.000006994	-0.000002955
O	0.000001718	0.000007500	0.000002537
H	0.000003098	-0.000002673	0.000000771
H	0.000006558	-0.000003583	0.000002170
H	0.000004596	0.000000668	0.000001840
H	0.000004245	-0.000006347	0.000000252
H	-0.000000282	-0.000006677	-0.000000531
H	-0.000007252	-0.000000569	-0.000003393
H	-0.000002386	0.000005039	-0.000000520
H	-0.000005826	0.000006394	-0.000000430
H	-0.000007831	0.000004140	-0.000002956
H	-0.000004756	-0.000003221	-0.000002264
H	-0.000001931	-0.000005393	-0.000003052
H	-0.000004586	-0.000004202	-0.000001977
H	0.000003287	-0.000001288	0.000001082
H	0.000001714	0.000004818	0.000002074
H	0.000001694	0.000002939	0.000004209

$R_2=CH_3$, $R_4=NO_2$, $R_6=CH_3$, $R_{2'}=H$				
**Geom.No 1=-1620.98688253 Ha**				
Atom	X	Y	Z	

C	0.000002867	-0.000002847	0.000000737	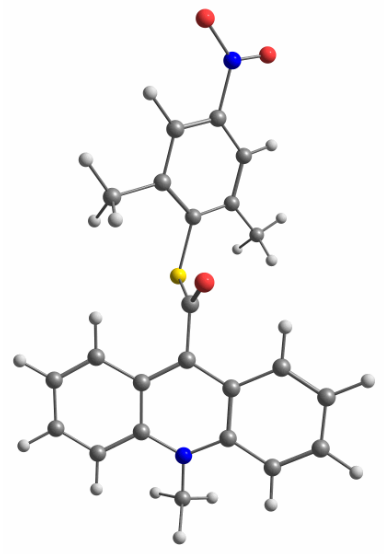
C	-0.000003737	-0.000002437	-0.000002344	
C	0.000000824	-0.000000122	0.000001567	
C	0.000001681	0.000001064	0.000004030	
C	0.000000199	-0.000002012	0.000001243	
C	0.000001582	-0.000003443	-0.000001195	
S	0.000000204	0.000009199	0.000001350	
C	0.000007636	-0.000031971	-0.000004779	
O	-0.000002936	0.000013362	0.000002883	
C	-0.000002177	0.000007609	0.000006408	
C	-0.000001047	0.000002969	-0.000004296	
C	0.000000732	0.000001608	-0.000002793	
N	-0.000000058	0.000001022	0.000000611	
C	-0.000003650	0.000002636	-0.000000446	
C	-0.000003578	0.000001978	-0.000001513	
C	0.000002056	-0.000002594	0.000000159	
C	0.000000509	-0.000001131	-0.000001258	
C	0.000000731	-0.000000525	-0.000002213	
C	-0.000000258	-0.000000094	-0.000000252	
C	-0.000002447	0.000002598	0.000000385	
C	-0.000003169	0.000004117	0.000000722	
C	-0.000000642	0.000002976	0.000000287	
C	0.000001881	-0.000000849	0.000001479	
C	0.000000143	0.000001426	-0.000002845	
H	-0.000000471	0.000000775	-0.000001729	
H	0.000000791	-0.000000920	-0.000002580	
H	0.000001488	-0.000001713	-0.000001889	
H	0.000000389	-0.000001467	-0.000000968	
H	-0.000001230	0.000001482	0.000001669	
H	-0.000002290	0.000002299	0.000002502	
H	-0.000002859	0.000003609	0.000001442	
H	-0.000001969	0.000003438	0.000000781	
H	-0.000001406	0.000001883	-0.000002316	
H	-0.000001329	0.000002220	-0.000000539	
H	-0.000001836	0.000003103	-0.000001041	
C	0.000000830	0.000001635	0.000005362	
H	0.000000772	-0.000000972	-0.000000067	
N	0.000011076	-0.000002305	-0.000003586	
H	0.000002398	-0.000002979	0.000001272	
C	-0.000003541	-0.000005628	-0.000002482	
O	-0.000001827	-0.000001363	0.000008443	
O	-0.000005610	-0.000004052	0.000001025	
H	0.000002027	-0.000003058	0.000001089	
H	0.000003572	0.000000123	0.000001299	
H	0.000004524	-0.000002071	-0.000000195	
H	-0.000001168	0.000000604	-0.000000668	
H	0.000000805	-0.000000229	-0.000001992	
H	-0.000000483	0.000001048	-0.000002762	

**Geom.No**
**2=-1772.15500104 Ha**

Atom	X	Y	Z
C	0.000000543	-0.000001159	0.000000292
C	0.000006239	-0.000000203	-0.000000190
C	-0.000003592	-0.000005210	0.000003222
C	0.000001336	-0.000000722	-0.000002534
C	0.000000519	-0.000001821	0.000000539
C	-0.000001417	-0.000000982	-0.000001380
C	-0.000006442	-0.000004012	-0.000009329
C	0.000003908	-0.000000023	0.000007767
C	0.000002206	-0.000005376	-0.000005930
N	-0.000000594	0.000010980	-0.000003463
C	-0.000000081	0.000001112	0.000001688
C	0.000003973	0.000000710	-0.000000894
C	0.000000888	0.000000239	-0.000001165
C	-0.000001151	0.000001191	0.000000476
C	-0.000002064	0.000011895	-0.000003667
O	0.000002010	-0.000002504	0.000002458
O	0.000007779	-0.000000987	0.000001014
O	-0.000003466	0.000002679	-0.000002206
C	0.000004304	-0.000004994	0.000001143
S	0.000006479	-0.000006262	0.000000012
C	-0.000000501	-0.000000784	-0.000001947
C	0.000005040	0.000015944	0.000010830
C	-0.000005964	-0.000012884	-0.000004995
C	0.000012116	0.000023533	0.000011781
C	-0.000005372	0.000000231	0.000001603
C	-0.000012196	0.000005751	0.000003499
H	0.000001204	-0.000002299	-0.000000009
H	0.000000427	-0.000002097	-0.000001001
H	0.000000740	-0.000001726	-0.000000480
H	0.000000210	-0.000000760	-0.000000343
H	0.000002527	0.000000648	0.000000937
H	0.000002151	0.000001993	0.000000163
H	0.000001872	0.000000176	-0.000000247
H	0.000001834	-0.000000792	-0.000000372
H	0.000000574	-0.000000957	-0.000001465
H	0.000000272	-0.000001892	-0.000001168
H	0.000000669	-0.000001116	-0.000000737
C	-0.000012708	-0.000004021	-0.000000169
H	-0.000001600	0.000000131	0.000001157
N	-0.000010069	-0.000021667	-0.000011282
H	-0.000002448	0.000000625	-0.000000042
C	0.000012271	-0.000004696	-0.000004262
H	-0.000001257	0.000000936	0.000000943
O	-0.000010560	0.000008219	0.000007000
O	0.000005846	0.000003275	0.000001005
H	-0.000003391	-0.000001887	0.000000579
H	-0.000002199	0.000000994	0.000002319
H	-0.000000352	-0.000000501	-0.000000029
H	-0.000001275	0.000001673	-0.000000380
H	0.000001736	-0.000001246	-0.000000431

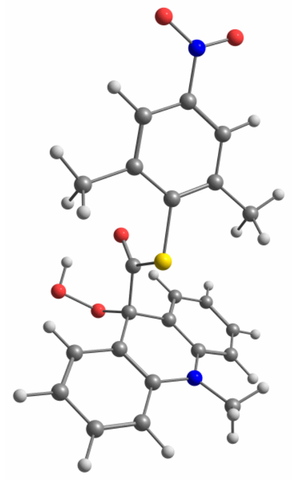

H	-0.000000970	0.000000645	-0.000000308	

**Geom.No 4= -913.060667296 Ha**				
Atom	X	Y	Z	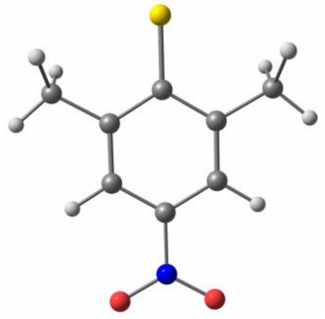
C	0.000010771	-0.000000000	-0.000014502	
C	0.000004752	-0.000000000	0.000068237	
C	0.000001675	-0.000000000	-0.000034361	
C	-0.000023535	0.000000000	-0.000015308	
C	-0.000014432	0.000000000	0.000032931	
C	0.000038178	0.000000000	-0.000013598	
C	0.000036581	-0.000000000	-0.000030807	
S	-0.000001648	-0.000000000	0.000005860	
C	0.000015682	0.000000000	0.000007730	
N	0.000003931	-0.000000000	-0.000011827	
O	0.000000965	-0.000000000	0.000044629	
O	-0.000013317	-0.000000000	-0.000015782	
H	-0.000004762	-0.000000000	0.000004805	
H	-0.000045135	-0.000000000	-0.000024251	
H	-0.000003185	0.000000000	-0.000004171	
H	0.000002781	0.000007661	-0.000019079	
H	0.000002781	-0.000007661	-0.000019079	
H	-0.000001997	0.000000000	0.000021960	
H	-0.000005043	0.000001879	0.000008305	
H	-0.000005043	-0.000001879	0.000008305	

**Geom.No 6= -1697.02689959 Ha**				
Atom	X	Y	Z	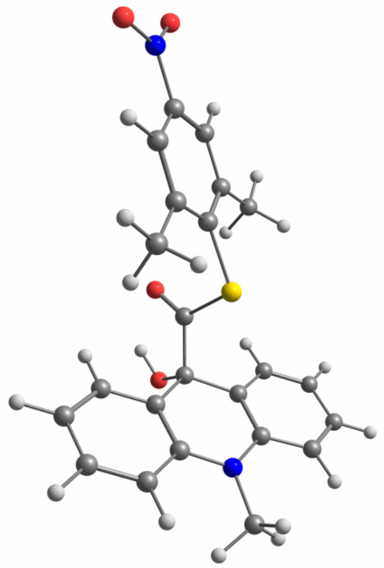
C	-0.000004515	-0.000002453	-0.000006064	
C	0.000000314	-0.000003155	-0.000017003	
C	-0.000014619	0.000002914	0.000008839	
C	0.000012974	0.000003917	-0.000000388	
C	-0.000034913	-0.000000977	0.000003740	
C	0.000008159	0.000003982	-0.000003318	
S	0.000006312	0.000015041	0.000011198	
C	-0.000014607	-0.000025871	-0.000002110	
O	0.000003785	0.000013401	-0.000002082	
C	0.000024708	-0.000019379	0.000013298	
C	0.000003815	-0.000003453	0.000001811	
C	0.000001440	0.000005997	0.000005437	
N	-0.000015940	-0.000014963	0.000009903	
C	-0.000004255	0.000006394	0.000004900	
C	-0.000010780	0.000001134	-0.000021036	
C	0.000001704	-0.000000929	-0.000002760	
C	-0.000001348	-0.000000585	0.000003116	
C	0.000008321	-0.000005889	0.000000012	
C	-0.000003580	-0.000003059	-0.000006829	

C	-0.000000661	0.000000254	0.000001323	
C	0.000001770	-0.000000620	-0.000001140	
C	-0.000005122	0.000007663	0.000001100	
C	0.000002789	-0.000001236	0.000008816	
O	-0.000011434	0.000019878	-0.000002058	
C	0.000016833	0.000004501	0.000000935	
H	0.000005075	-0.000004586	0.000004674	
H	0.000001732	-0.000004297	0.000000107	
H	0.000003402	-0.000002893	-0.000003117	
H	-0.000000036	-0.000001925	-0.000004739	
H	0.000000117	0.000003663	-0.000001433	
H	-0.000001876	0.000002869	0.000002710	
H	-0.000001398	0.000002947	0.000004523	
H	0.000003673	0.000000363	-0.000005748	
H	-0.000000486	-0.000002576	0.000003057	
H	0.000002124	-0.000001158	0.000004257	
H	0.000002482	-0.000002471	0.000012912	
C	0.000008063	-0.000012653	-0.000000385	
H	-0.000000841	-0.000001465	-0.000002071	
N	0.000026192	0.000004939	-0.000012460	
H	-0.000001116	0.000001800	-0.000002676	
C	-0.000004250	0.000002532	0.000008313	
H	-0.000001695	-0.000001267	0.000000009	
O	-0.000006522	-0.000000665	-0.000002401	
O	-0.000005483	-0.000001624	-0.000001393	
H	-0.000002744	0.000001233	-0.000003781	
H	-0.000002097	0.000003740	-0.000003872	
H	0.000003005	0.000006204	-0.000001504	
H	0.000001442	0.000000482	-0.000002028	
H	0.000000638	0.000002521	-0.000000836	
H	-0.000000548	0.000001781	-0.000001761	

**Geom.No 7=-1696.46324230 Ha**				
Atom	X	Y	Z	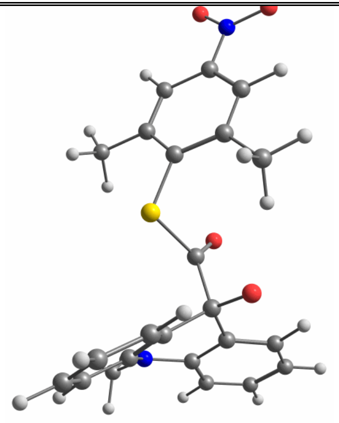
C	0.000002583	0.000005008	0.000000891	
C	-0.000000168	0.000003525	0.000000635	
C	0.000001564	0.000003954	0.000004090	
C	0.000000491	0.000005087	0.000004332	
C	0.000002131	0.000006982	0.000002558	
C	0.000005563	0.000007106	0.000002294	
C	0.000012843	0.000002181	0.000005979	
C	-0.000001629	-0.000000949	-0.000004765	
C	0.000001791	0.000000957	-0.000000666	
N	-0.000000596	-0.000001319	-0.000000058	
C	-0.000000269	-0.000000892	-0.000001642	
C	-0.000000907	-0.000003278	-0.000003257	
C	0.000001813	-0.000003770	-0.000003915	
C	0.000001748	0.000000259	-0.000004499	
C	0.000009009	0.000004928	-0.000017636	
O	-0.000001279	-0.000003142	0.000006700	

O	-0.000003755	0.000001551	-0.000006258
C	-0.000000925	0.000001349	0.000003461
S	-0.000014328	0.000000073	0.000008419
C	0.000002021	-0.000004919	-0.000004235
C	0.000001158	-0.000004506	-0.000003267
C	0.000002188	-0.000001440	0.000001024
C	-0.000011188	0.000006107	-0.000006923
C	-0.000001111	-0.000005115	0.000005908
C	-0.000003196	-0.000002682	0.000002986
C	-0.000004186	-0.000005303	0.000001072
H	0.000001073	-0.000003948	-0.000006507
H	0.000002467	-0.000001364	-0.000004578
H	-0.000000517	-0.000004093	-0.000003519
H	-0.000001230	-0.000001832	0.000000074
H	0.000000858	0.000004903	0.000004338
H	0.000004931	0.000005084	-0.000000496
H	0.000004799	0.000008469	0.000002187
H	0.000003244	0.000008215	0.000004970
H	-0.000002048	0.000001925	0.000004625
H	-0.000002904	-0.000000504	0.000003060
H	-0.000001434	0.000001620	0.000003008
H	-0.000004773	-0.000003712	0.000002065
N	0.000007781	-0.000011015	0.000005199
H	0.000000860	-0.000001334	-0.000002191
C	0.000005141	0.000003798	-0.000000729
O	-0.000004428	-0.000001317	-0.000000860
O	-0.000005211	0.000000482	0.000001303
H	0.000002065	-0.000000055	-0.000003233
H	0.000004077	-0.000000197	-0.000002643
H	0.000000522	-0.000003561	-0.000003949
H	-0.000006334	-0.000004947	0.000001906
H	-0.000005657	-0.000005353	0.000000110
H	-0.000004648	-0.000003019	0.000002633

Geom.No
TS-VIII= -1696.46129789 Ha

Atom	X	Y	Z
C	0.000001164	-0.000000538	-0.000001900
C	-0.000000744	0.000001844	-0.000000897
C	-0.000001156	0.000001015	-0.000000071
C	0.000000759	-0.000000466	0.000000028
C	-0.000000019	-0.000000376	0.000000002
C	0.000000113	-0.000001468	0.000000286
S	-0.000006966	0.000000390	-0.000008793
C	0.000001792	-0.000001881	-0.000000734
C	0.000001715	0.000004262	0.000003280
C	0.000005214	0.000006543	0.000007628
C	0.000001305	-0.000001911	0.000000649
C	0.000000887	-0.000006799	0.000001135
C	0.000001985	-0.000000231	0.000002590
C	0.000002689	-0.000006298	0.000000073

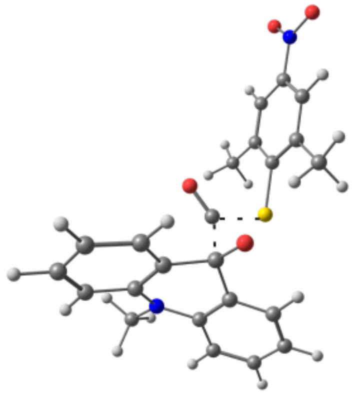

S	0.000002109	-0.000009714	0.000000553	
C	0.000007546	-0.000004280	0.000009073	
O	-0.000002999	0.000000594	-0.000006364	
C	0.000001050	-0.000000517	0.000002843	
N	0.000002425	-0.000000414	0.000002048	
O	0.000002549	-0.000003358	0.000002987	
C	0.000001978	-0.000003760	-0.000003159	
O	0.000004094	-0.000001067	0.000003928	
C	-0.000016412	0.000000833	-0.000000600	
C	0.000003611	0.000003190	-0.000000326	
C	0.000000140	0.000000473	-0.000001495	
N	0.000001507	-0.000000228	-0.000002087	
C	-0.000006921	0.000001513	-0.000001303	
C	0.000001188	0.000001586	0.000000993	
C	-0.000000827	-0.000000211	-0.000000639	
C	0.000000146	-0.000000030	0.000000052	
C	0.000000589	0.000000826	0.000000595	
C	0.000002080	0.000002036	0.000000161	
C	-0.000002802	0.000001278	-0.000003153	
C	-0.000003460	0.000002249	-0.000003295	
C	-0.000002385	0.000003240	-0.000003308	
C	0.000000006	0.000002767	-0.000001971	
O	0.000000732	0.000000518	-0.000002130	
C	-0.000005061	-0.000000974	-0.000001604	
H	0.000002035	0.000001066	0.000001191	
H	0.000002239	0.000001807	0.000000536	
H	0.000000049	-0.000000339	0.000000224	
H	-0.000002090	-0.000000862	-0.000001309	
H	-0.000003900	0.000000750	-0.000003637	
H	-0.000001210	0.000003315	-0.000001698	
H	-0.000002429	0.000003851	-0.000003389	
H	-0.000004323	0.000002509	-0.000004228	
H	-0.000002297	-0.000000197	-0.000002164	
H	-0.000004061	-0.000001384	-0.000001004	
H	-0.000003459	-0.000001743	-0.000003628	
H	0.000001185	-0.000004198	0.000001852	
H	0.000003153	0.000001044	0.000002972	
H	0.000004452	0.000004300	0.000002547	
H	0.000004344	0.000010633	-0.000002799	
H	0.000001601	-0.000000466	0.000003408	
H	0.000000833	-0.000001333	0.000003046	
H	-0.000003029	-0.000005514	0.000001808	
H	0.000002228	-0.000005354	-0.000000881	

**R_2=Cl, R_4=H, R_6=H , $R_{2'}$=H**				
**Geom.No**<br>**1=-1797.44584492 Ha**				
Atom	X	Y	Z	

C	-0.000002385	0.000001344	0.000002147	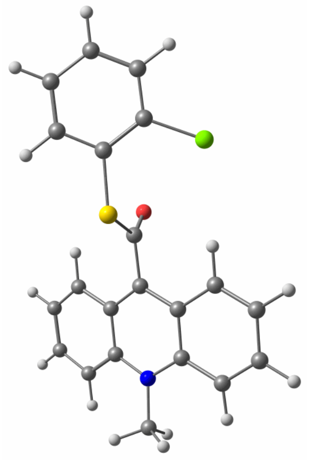
C	0.000001217	0.000002076	-0.000000564	
C	0.000002280	0.000000824	-0.000000603	
C	0.000001252	0.000000258	-0.000000798	
C	0.000000248	0.000000256	-0.000000564	
C	0.000005388	0.000001168	-0.000001101	
H	0.000001644	0.000002582	0.000000304	
H	0.000001868	0.000001493	-0.000000333	
H	0.000001640	0.000000473	-0.000001004	
H	0.000000931	-0.000000837	-0.000001092	
C	-0.000003486	-0.000000592	-0.000001575	
C	-0.000001300	0.000000735	0.000001297	
C	-0.000001210	0.000001221	-0.000000341	
N	0.000001732	0.000001113	0.000000155	
C	0.000003463	-0.000003669	-0.000000615	
C	0.000000067	0.000003093	0.000002777	
C	0.000000368	0.000000973	0.000001076	
C	-0.000000737	0.000000521	0.000000463	
C	-0.000000096	0.000001714	0.000000546	
C	-0.000000156	0.000002425	0.000001701	
H	-0.000000248	-0.000000204	0.000000348	
H	-0.000001496	0.000000567	0.000000951	
H	-0.000000998	0.000001622	0.000001307	
H	-0.000000077	0.000002529	0.000000507	
H	0.000000084	0.000001991	-0.000000069	
H	0.000000517	0.000003030	0.000000431	
H	0.000000195	0.000002462	0.000001582	
S	-0.000000095	0.000001327	0.000001704	
O	-0.000000768	0.000003048	0.000000583	
C	-0.000004292	-0.000008635	0.000003587	
C	-0.000000337	0.000001447	-0.000004083	
C	-0.000005291	-0.000002897	-0.000002621	
C	0.000001362	-0.000004904	0.000003321	
C	0.000001078	-0.000001655	-0.000004665	
C	-0.000001350	-0.000001776	-0.000000733	
Cl	0.000001106	-0.000002526	0.000000621	
H	-0.000000028	-0.000003387	-0.000000431	
H	-0.000001391	-0.000003641	-0.000001917	
H	-0.000001315	-0.000003609	-0.000000432	
H	0.000000614	-0.000001961	-0.000001867	

Geom.No<br>2=-1948.60575950 Ha				
Atom	X	Y	Z	

C	0.000001005	0.000000242	-0.000000417
C	-0.000006018	-0.000002838	0.000006450
C	-0.000000704	0.000005276	-0.000006324
C	-0.000000486	-0.000001212	-0.000002031
C	-0.000000111	-0.000001216	-0.000002675
C	-0.000001902	-0.000000401	-0.000004226
C	0.000006331	-0.000031100	-0.000030534
C	-0.000004639	0.000013143	0.000013846
C	0.000002511	0.000005154	0.000002661
N	0.000003071	-0.000007958	0.000001989
C	0.000005027	-0.000005393	-0.000007411
C	-0.000002094	-0.000002985	0.000003330
C	0.000004222	0.000002687	0.000002868
C	0.000003316	-0.000001639	-0.000005431
C	-0.000007179	-0.000094036	0.000036402
O	0.000004077	-0.000003746	-0.000004417
O	0.000018714	0.000004745	-0.000001495
O	-0.000020770	0.000006967	0.000000416
C	0.000003317	0.000002140	-0.000002027
S	0.000009341	0.000111441	-0.000016465
C	-0.000017676	-0.000003867	0.000002645
C	0.000039188	-0.000012813	-0.000008881
C	-0.000001585	0.000001974	0.000001851
C	-0.000003848	0.000003405	0.000005144
C	-0.000001324	0.000001903	0.000000377
C	0.000000338	0.000003701	0.000003062
Cl	-0.000037280	0.000013307	0.000012099
H	0.000001788	-0.000001729	0.000001877
H	0.000004632	-0.000001556	0.000000272
H	0.000002486	-0.000002231	0.000000738
H	0.000001303	-0.000001679	0.000001323
H	0.000000081	0.000000044	-0.000001970
H	-0.000002717	-0.000000592	-0.000002168
H	-0.000001275	-0.000000136	-0.000003071
H	0.000000333	-0.000001659	-0.000003345
H	0.000001719	-0.000001594	0.000000088
H	0.000000515	-0.000002293	-0.000001996
H	0.000001048	-0.000000743	-0.000001255
H	-0.000000002	0.000001082	0.000002562
H	-0.000001104	0.000002562	0.000004061
H	-0.000002186	0.000003234	0.000002495
H	-0.000003750	0.000002707	0.000001083
H	0.000002285	-0.000002297	-0.000001501

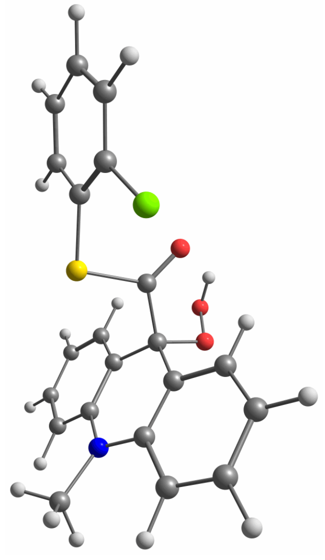

Geom.No
4= -1089.48594898 Ha

Atom	X	Y	Z

C	0.000036562	0.000000000	0.000218856
C	-0.000115830	0.000000000	-0.000235487
C	0.000207937	0.000000000	0.000001679
C	-0.000148877	0.000000000	0.000101248
C	-0.000007875	0.000000000	-0.000026266
C	0.000007320	0.000000000	-0.000024318
H	0.000035890	0.000000000	0.000069407
H	-0.000056299	0.000000000	0.000003611
H	0.000027151	0.000000000	-0.000036677
H	-0.000009015	0.000000000	0.000005834
CL	0.000018581	0.000000000	-0.000086856
S	0.000004457	0.000000000	0.000008970

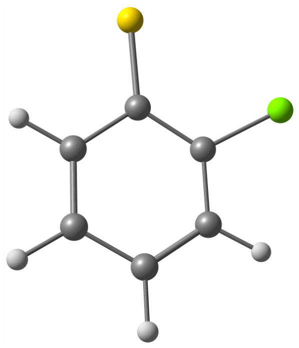

Atom	X	Y	Z
C	-0.000016311	-0.000000200	0.000011791
C	-0.000002006	0.000001660	-0.000003012
C	0.000002889	0.000001416	-0.000025394
C	-0.000002125	0.000003017	-0.000006994
C	-0.000001767	0.000000910	-0.000003484
C	0.000008116	0.000002498	-0.000012692
C	-0.000004705	0.000004871	0.000002091
C	-0.000007246	-0.000003520	0.000009263
C	-0.000001122	0.000000339	0.000001079
N	0.000003684	0.000003381	0.000027421
C	0.000003041	-0.000001708	0.000000083
C	0.000001586	-0.000001672	-0.000000585
C	0.000002957	-0.000002467	0.000001081
C	0.000012711	-0.000000511	-0.000008554
C	-0.000002433	-0.000004316	0.000003180
O	-0.000000612	0.000001998	0.000001987
O	0.000000711	-0.000005286	0.000002995
C	0.000001814	0.000002100	-0.000000006
S	0.000001168	0.000000605	0.000000688
C	-0.000001228	0.000000018	-0.000001152
C	0.000002177	-0.000000536	-0.000001149
C	-0.000001051	0.000003287	0.000002760
C	-0.000002580	-0.000002758	0.000002127
C	0.000002719	0.000000776	-0.000001571
C	-0.000001882	-0.000003436	0.000006749
C	0.000004342	-0.000001001	0.000001187
CL	0.000004289	-0.000000412	-0.000002707
H	0.000005088	-0.000001931	0.000000167
H	0.000002910	-0.000002394	0.000002881
H	0.000000384	-0.000001378	0.000003232
H	-0.000003714	0.000000986	-0.000000037
H	-0.000002703	0.000002222	-0.000001880
H	-0.000003151	0.000003630	-0.000003392
H	-0.000001697	0.000000800	-0.000005211
H	0.000000460	-0.000000600	-0.000001607

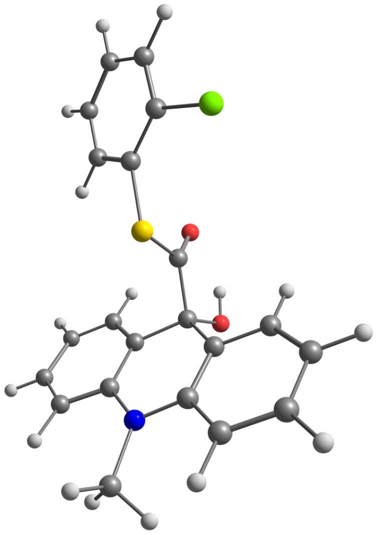

H	0.000001696	0.000000602	-0.000003765
H	-0.000000586	0.000001054	-0.000003413
H	0.000001983	-0.000001987	0.000001786
H	-0.000000729	-0.000000480	0.000000728
H	-0.000002972	-0.000000745	0.000000582
H	-0.000001915	0.000000894	-0.000000309
H	-0.000002190	0.000000273	0.000003054

Geom.No
7=-1872.90368892 Ha

Atom	X	Y	Z
C	0.000000117	0.000001756	0.000001936
C	0.000011562	0.000004039	-0.000000951
C	0.000002070	-0.000001540	-0.000004592
C	-0.000000181	0.000002900	0.000004991
C	0.000001824	0.000003184	0.000002334
C	0.000003476	0.000002226	0.000000835
C	-0.000006389	-0.000021922	0.000004060
C	0.000001488	0.000003968	-0.000002142
C	-0.000003253	-0.000001104	0.000004726
N	0.000000154	0.000008825	0.000004026
C	-0.000000325	-0.000000033	-0.000001210
C	-0.000002153	-0.000000594	-0.000000370
C	-0.000000216	-0.000000442	0.000000843
C	-0.000000286	-0.000002294	-0.000002673
C	-0.000009455	0.000005009	-0.000006612
O	-0.000000255	0.000009349	-0.000006335
O	0.000007171	0.000002597	0.000002744
C	0.000001538	-0.000001585	0.000000966
S	-0.000002631	-0.000010242	0.000012917
C	0.000004325	0.000012972	-0.000018820
C	0.000017536	-0.000015985	0.000005148
C	-0.000008989	0.000009078	-0.000002941
C	-0.000004140	-0.000006357	-0.000011578
C	0.000013089	-0.000006574	-0.000001812
C	-0.000018888	0.000001262	-0.000007397
Cl	-0.000002286	-0.000001727	0.000019333
H	-0.000000001	-0.000001961	-0.000002390
H	0.000000879	-0.000002089	-0.000001776
H	-0.000000406	-0.000001069	-0.000000973
H	-0.000001539	-0.000000224	0.000001057
H	0.000001950	0.000002855	0.000003965
H	0.000002717	0.000000521	-0.000000109
H	0.000003809	0.000002723	0.000001378
H	0.000002870	0.000003212	0.000003037
H	-0.000000767	0.000002577	0.000002192
H	-0.000001749	0.000001649	0.000001398
H	-0.000000212	0.000002126	0.000002702
H	-0.000004647	-0.000002002	-0.000001431
H	-0.000002249	-0.000003161	-0.000000697
H	0.000001675	-0.000002669	-0.000002805

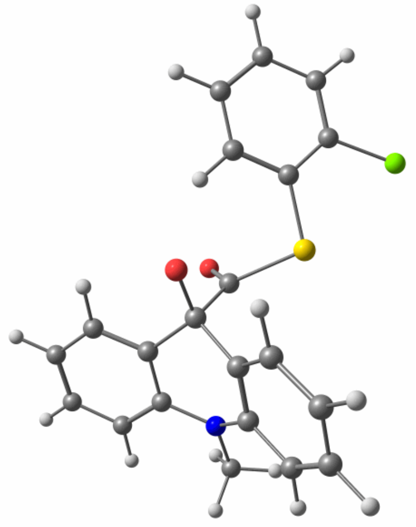

H	-0.000007231	0.000000742	-0.000002975	

Geom.No
VIII-TS=-1872.89862260 Ha

Atom	X	Y	Z	
C	-0.000003440	0.000001371	0.000012564	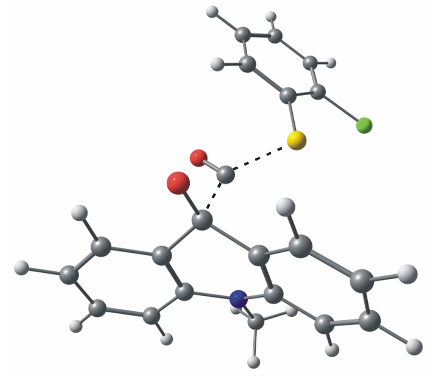
C	-0.000004888	0.000000075	-0.000013338	
C	0.000012758	-0.000003587	0.000006103	
C	-0.000003952	-0.000001096	0.000007307	
C	-0.000005485	0.000001395	-0.000004921	
C	0.000010833	-0.000000300	-0.000000159	
S	0.001456674	0.001381989	0.001341436	
C	-0.003253222	-0.001005131	-0.001009434	
O	-0.000002983	0.000002279	0.000000125	
CL	-0.000000104	-0.000001890	-0.000001083	
C	0.001802146	-0.000363739	-0.000336389	
C	0.000002122	-0.000003018	0.000010784	
C	-0.000002375	0.000001942	-0.000001311	
N	-0.000000673	-0.000002884	0.000000415	
C	0.000000256	0.000006850	-0.000004276	
C	-0.000002189	-0.000015832	-0.000002229	
C	0.000000257	-0.000001534	-0.000000420	
C	-0.000001572	-0.000003248	0.000001419	
C	-0.000001158	-0.000001024	0.000001011	
C	0.000003801	-0.000001035	0.000000989	
C	-0.000001797	0.000001062	-0.000002271	
C	0.000000441	0.000001970	-0.000001755	
C	-0.000002221	0.000002801	-0.000001872	
C	0.000001450	0.000004467	-0.000000088	
O	-0.000004203	0.000001129	0.000004157	
C	-0.000000562	-0.000001143	-0.000003325	
H	0.000001050	-0.000001915	0.000001999	
H	0.000000729	0.000000241	0.000001203	
H	0.000000916	-0.000003526	0.000000666	
H	0.000000002	-0.000003353	-0.000001004	
H	-0.000000925	-0.000000112	-0.000001805	
H	0.000000145	0.000003029	0.000000572	
H	-0.000000357	0.000003696	-0.000001438	
H	-0.000000716	0.000002284	-0.000002486	
H	-0.000001839	-0.000001159	-0.000003111	
H	-0.000000402	-0.000001524	-0.000001331	
H	-0.000000727	-0.000000935	-0.000001981	
H	0.000001037	-0.000001651	0.000000212	
H	0.000001271	0.000000525	0.000002321	
H	-0.000000774	0.000001768	0.000002280	
H	0.000000673	0.000000766	0.000000464	

R_2=Cl, R_4=H, R_6=CL, $R_{2'}$=H

Geom.No
1= -2257.03357189 Ha

Atom	X	Y	Z
C	0.000010930	-0.000010619	-0.000018459
C	-0.000001969	0.000018039	0.000018962
C	-0.000014100	-0.000013909	0.000000942
C	0.000011278	-0.000004113	-0.000011889
C	0.000005258	0.000013467	0.000010272
C	-0.000013882	-0.000007306	0.000007211
S	-0.000002107	0.000002638	-0.000010918
C	0.000006771	-0.000008121	0.000010641
O	-0.000001736	0.000001307	-0.000002425
Cl	0.000001875	0.000003193	0.000002771
C	0.000004258	-0.000002328	-0.000007945
C	-0.000006817	0.000003518	0.000005573
C	0.000005678	0.000005329	-0.000002839
N	-0.000003971	-0.000005805	-0.000002566
C	0.000003228	0.000006128	0.000005168
C	-0.000001319	0.000004854	0.000003851
C	0.000001038	-0.000004145	-0.000000815
C	-0.000003309	0.000002548	0.000001941
C	0.000003281	0.000001290	0.000000545
C	-0.000001577	-0.000002703	-0.000001559
C	-0.000002036	-0.000007261	-0.000005477
C	-0.000005290	0.000003946	0.000002946
C	0.000007169	0.000001806	-0.000001072
C	-0.000002081	-0.000006626	-0.000005192
C	0.000001382	0.000004149	-0.000002233
H	-0.000001148	0.000000004	-0.000000984
H	-0.000000687	0.000000701	-0.000001209
H	0.000000581	-0.000000260	-0.000000038
H	0.000000482	0.000000561	0.000000527
H	0.000000317	0.000000646	-0.000000129
H	-0.000000943	-0.000000123	-0.000000131
H	0.000000379	-0.000000529	-0.000000334
H	0.000000964	-0.000000460	-0.000001607
H	0.000000784	0.000001371	0.000000881
H	0.000000103	0.000000594	-0.000000799
H	-0.000001021	0.000001494	0.000000292
H	-0.000001249	-0.000000448	0.000002773
H	-0.000000059	-0.000002692	-0.000000093
H	0.000002160	-0.000000348	-0.000000121
Cl	-0.000002615	0.000000215	0.000003540

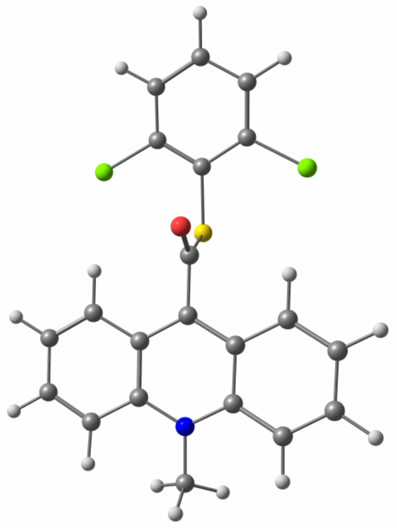

Geom.No
2=-2408.19359459 Ha

Atom	X	Y	Z

C	0.000010645	-0.000006259	-0.000010190	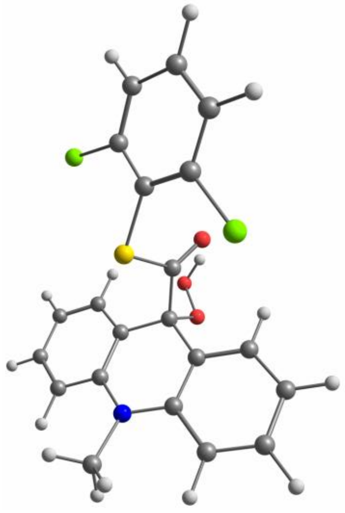
C	-0.000002604	-0.000008641	0.000013095	
C	-0.000023281	-0.000002907	-0.000009351	
C	0.000007316	-0.000007582	-0.000005697	
C	0.000000425	0.000004929	0.000007354	
C	-0.000011038	-0.000003339	0.000003996	
C	-0.000013711	0.000014684	0.000004557	
C	-0.000006369	-0.000019149	-0.000034830	
C	0.000002579	0.000012612	-0.000006716	
N	0.000018835	-0.000030090	0.000019171	
C	-0.000000223	-0.000000502	-0.000001391	
C	0.000006286	-0.000000024	-0.000001088	
C	-0.000002211	0.000005404	-0.000001116	
C	0.000006659	0.000005545	0.000008898	
C	0.000029579	0.000028550	0.000054393	
O	-0.000020099	-0.000002272	-0.000024789	
O	-0.000004482	-0.000010697	0.000001897	
O	-0.000002390	0.000005625	0.000009984	
C	0.000003141	0.000002107	-0.000001711	
S	-0.000051994	0.000004641	-0.000080983	
C	0.000047044	-0.000002979	0.000069997	
C	0.000038309	-0.000009014	-0.000005700	
C	0.000001613	-0.000000925	-0.000010638	
C	0.000000158	0.000000877	0.000001075	
C	-0.000006782	0.000005182	0.000000348	
C	0.000054448	-0.000031371	-0.000108170	
Cl	-0.000042284	0.000007706	0.000010214	
Cl	-0.000054612	0.000035360	0.000105888	
H	0.000006426	0.000002806	-0.000003704	
H	0.000003123	0.000004629	-0.000002465	
H	0.000002994	0.000002766	0.000000965	
H	-0.000000874	0.000004729	0.000001719	
H	0.000012659	0.000000741	0.000003211	
H	-0.000002284	-0.000003611	0.000001300	
H	-0.000002431	-0.000005587	-0.000000569	
H	0.000000299	-0.000002816	0.000002508	
H	-0.000002961	0.000003701	-0.000002922	
H	-0.000002249	-0.000003149	-0.000001352	
H	0.000003325	-0.000002191	0.000000561	
H	0.000001852	0.000002740	-0.000001791	
H	0.000000654	0.000001254	-0.000000987	
H	-0.000001663	-0.000000549	0.000000409	
H	-0.000003825	-0.000002933	-0.000005378	

**Geom.No**
**4=-1549.08306463 Ha**

Atom	X	Y	Z
C	-0.000083887	0.000000000	0.000139745
C	0.000029647	0.000000000	-0.000014795
C	0.000000000	0.000000000	0.000009175
C	-0.000029647	0.000000000	-0.000014795
C	0.000083887	0.000000000	0.000139745
C	0.000000000	0.000000000	-0.000214606
H	-0.000007245	0.000000000	-0.000001997
H	0.000000000	0.000000000	-0.000001169
H	0.000007245	0.000000000	-0.000001997
Cl	0.000001956	0.000000000	-0.000030942
S	0.000000000	0.000000000	0.000022578
Cl	-0.000001956	0.000000000	-0.000030942

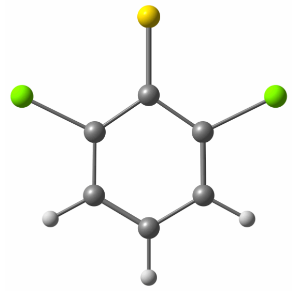

Geom.No
6=-2333.06497014 Ha

Atom	X	Y	Z
C	0.000006631	0.000000486	-0.000006837
C	-0.000077321	0.000047779	0.000040692
C	0.000162883	0.000094815	0.000063727
C	0.000002687	0.000001863	-0.000002033
C	0.000000479	0.000001619	-0.000003730
C	-0.000000982	0.000005075	0.000000755
S	0.000066734	0.000020891	-0.000144477
C	0.000006391	-0.000096630	0.000115368
O	-0.000005678	0.000001130	-0.000016732
C	-0.000023697	0.000012268	0.000011570
C	0.000016742	0.000008076	0.000001932
C	0.000000998	-0.000004288	-0.000003678
N	0.000009623	-0.000004427	0.000006455
C	-0.000006579	-0.000000805	-0.000001221
C	0.000004453	0.000001165	-0.000011819
C	-0.000008913	-0.000005645	0.000001388
C	0.000007774	0.000003733	-0.000003778
C	-0.000002569	0.000000125	0.000006318
C	-0.000001935	-0.000005116	-0.000000823
C	0.000003305	0.000002662	0.000001901
C	-0.000004448	-0.000002163	0.000003906
C	-0.000001800	-0.000002241	-0.000000351
C	0.000002832	0.000008116	0.000001774
O	-0.000014060	-0.000002635	-0.000014469
C	-0.000006760	0.000003600	0.000003741
H	0.000000244	-0.000001177	-0.000001540
H	0.000000852	-0.000001722	-0.000000904
H	0.000000822	-0.000000945	0.000000090
H	0.000002587	0.000002452	-0.000001723
H	-0.000007194	0.000004311	0.000007118
H	-0.000001254	0.000000376	0.000001280
H	-0.000000895	-0.000000371	0.000001098
H	-0.000000315	-0.000001027	0.000000852

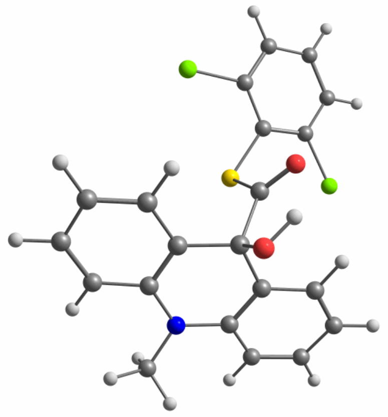

H	0.000000425	-0.000000722	0.000002677
H	0.000000392	-0.000002947	-0.000001411
H	-0.000001039	-0.000003926	0.000002036
Cl	-0.000162915	-0.000095572	-0.000059190
H	0.000001471	0.000002143	-0.000002338
H	0.000001280	0.000002585	-0.000002191
H	0.000000763	0.000002609	-0.000001835
Cl	0.000015702	-0.000003215	0.000002511
H	0.000012282	0.000007696	0.000003890

Geom.No
7=-2332.49890674 Ha

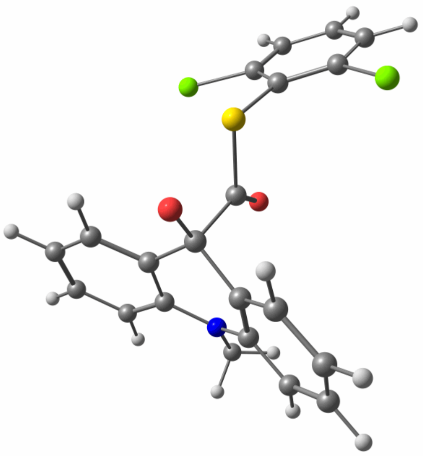

Atom	X	Y	Z
C	0.000001203	-0.000000059	-0.000005666
C	0.000014586	-0.000003738	0.000003070
C	-0.000001751	-0.000003907	-0.000001421
C	0.000001979	0.000000925	-0.000007041
C	-0.000001081	0.000001013	0.000000023
C	-0.000006026	-0.000000313	-0.000002165
C	-0.000041902	0.000050966	-0.000004474
C	0.000018695	-0.000016314	0.000024556
C	-0.000003560	-0.000002328	-0.000008102
N	-0.000000930	0.000013456	0.000003549
C	0.000002099	0.000003336	-0.000001431
C	0.000006674	0.000002637	0.000000705
C	0.000001642	-0.000000915	-0.000000087
C	0.000000069	0.000001210	0.000000313
C	-0.000020694	-0.000052778	-0.000037202
O	0.000014771	0.000023259	0.000015769
O	0.000009479	-0.000001085	0.000004458
C	-0.000000009	-0.000004267	-0.000000265
S	0.000013007	0.000008345	0.000020758
C	-0.000001714	-0.000046845	-0.000016776
C	-0.000014783	0.000001096	0.000015689
C	0.000003287	0.000003858	-0.000000022
C	-0.000001089	-0.000001313	0.000001833
C	-0.000002404	0.000004594	-0.000000427
C	0.000004027	0.000009470	-0.000001459
Cl	-0.000001875	-0.000000235	0.000003714
Cl	0.000004118	-0.000001441	-0.000001976
H	0.000004271	0.000000047	0.000004007
H	0.000001848	0.000000547	0.000000645
H	0.000005708	0.000001237	0.000001249
H	0.000005428	0.000001859	0.000000479
H	-0.000000808	0.000001775	-0.000005968
H	-0.000004378	-0.000000467	0.000001597
H	-0.000002980	-0.000000472	-0.000001558
H	-0.000002189	0.000000933	-0.000003199
H	-0.000000433	0.000003955	-0.000000386
H	0.000001652	-0.000000085	-0.000001799
H	0.000003098	0.000002748	-0.000004479

H	-0.000004757	0.000000433	-0.000002889	
H	-0.000003463	-0.000000775	0.000000994	
H	-0.000000814	-0.000000361	0.000005385	

Geom.No
VIII-TS=-2332.49254956 Ha

Atom	X	Y	Z	
C	0.000000382	0.000007030	-0.000010585	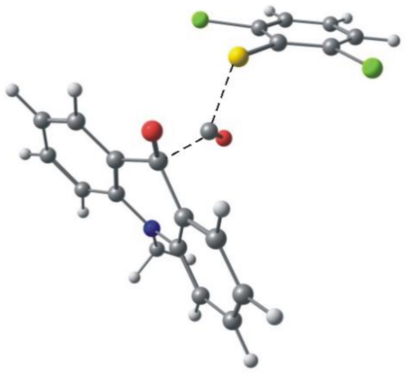
C	-0.000014772	0.000009050	0.000007021	
C	0.000013945	-0.000000031	0.000010137	
C	-0.000007529	0.000005870	-0.000000415	
C	0.000002226	0.000005416	0.000002662	
C	-0.000003748	0.000004621	0.000008800	
S	-0.003201825	-0.010534701	-0.001485938	
C	0.009470510	0.010548800	-0.002527779	
O	0.000012605	-0.000031222	-0.000019634	
Cl	-0.000003392	0.000001562	-0.000002286	
Cl	-0.000001846	0.000001878	0.000009063	
C	-0.006283130	0.000017905	0.004034579	
C	0.000001459	-0.000010710	-0.000003994	
C	0.000011774	0.000004624	-0.000003149	
N	-0.000021606	-0.000006773	0.000010103	
C	0.000007697	0.000005155	-0.000008825	
C	0.000001077	-0.000009924	-0.000003370	
C	0.000001310	0.000003694	-0.000001484	
C	0.000002005	-0.000007163	-0.000002263	
C	0.000000053	0.000003826	0.000002472	
C	-0.000000907	0.000001550	0.000000708	
C	0.000004033	0.000001299	0.000000561	
C	0.000002302	-0.000010793	-0.000006129	
C	0.000000307	0.000002255	-0.000000263	
C	0.000000547	-0.000002849	-0.000000193	
O	-0.000006250	-0.000003447	0.000005311	
C	0.000005898	0.000000270	-0.000005601	
H	0.000001239	-0.000005512	-0.000001342	
H	-0.000001562	-0.000004065	0.000002788	
H	0.000002061	-0.000004083	-0.000003046	
H	0.000001095	-0.000003776	-0.000005530	
H	0.000002564	-0.000003265	-0.000000656	
H	-0.000003622	-0.000002380	0.000001720	
H	-0.000000560	-0.000001911	0.000000572	
H	0.000000504	-0.000000488	-0.000000910	
H	0.000001489	0.000001019	-0.000004939	
H	0.000004656	-0.000000394	-0.000003335	
H	0.000004801	-0.000000999	-0.000004084	
H	-0.000002328	0.000006820	0.000002476	
H	-0.000002128	0.000007592	0.000003143	
H	-0.000001335	0.000004247	0.000003629	

R_2=CH_3, R_4=H, R_6=H, $R_{2'}$=H

Geom.No
1=-1377.17512149 Ha

Atom	X	Y	Z
C	-0.000000692	0.000005866	-0.000003920
C	-0.000036742	0.000010067	0.000028748
C	0.000002307	0.000001892	-0.000005670
C	0.000000107	0.000000825	0.000000592
C	-0.000001920	0.000007830	-0.000005361
C	0.000000538	0.000000833	-0.000001242
S	0.000054316	-0.000008899	-0.000006952
C	-0.000018942	0.000003871	-0.000036288
O	0.000000322	-0.000000968	0.000006770
C	0.000000062	0.000002593	-0.000003557
C	-0.000015809	0.000000654	0.000012743
C	0.000014224	-0.000001747	-0.000006898
C	-0.000008562	-0.000005656	0.000011733
N	0.000003786	0.000000436	0.000002141
C	-0.000001005	-0.000011267	-0.000000825
C	0.000004530	-0.000004828	-0.000002216
C	-0.000002921	0.000002611	0.000003136
C	-0.000001221	-0.000002655	0.000002953
C	0.000000371	-0.000002700	0.000001773
C	-0.000001566	-0.000000063	0.000001726
C	0.000003598	0.000009699	0.000005363
C	0.000007511	-0.000004683	-0.000005582
C	-0.000004206	-0.000003595	0.000003227
C	0.000000508	0.000005730	-0.000000127
C	-0.000003306	0.000002559	0.000001904
H	0.000000026	-0.000002777	0.000001073
H	-0.000001985	-0.000002346	0.000003778
H	-0.000002364	-0.000000984	0.000003687
H	-0.000002164	-0.000000701	0.000001705
H	-0.000000459	0.000000734	-0.000001287
H	0.000000935	0.000000360	-0.000002258
H	0.000002530	-0.000002207	0.000001270
H	0.000003083	-0.000005087	0.000001862
H	0.000003390	-0.000007537	0.000003359
H	0.000001561	-0.000005534	0.000002296
H	0.000001111	-0.000004283	0.000001780
H	-0.000000829	0.000004786	-0.000004562
H	-0.000001552	0.000004990	-0.000004353
H	-0.000003705	0.000004859	-0.000001753
H	-0.000000456	0.000002310	-0.000002215
H	0.000000999	0.000001586	-0.000003862
H	0.000002939	0.000001657	-0.000002721
H	0.000001652	0.000001767	-0.000001970

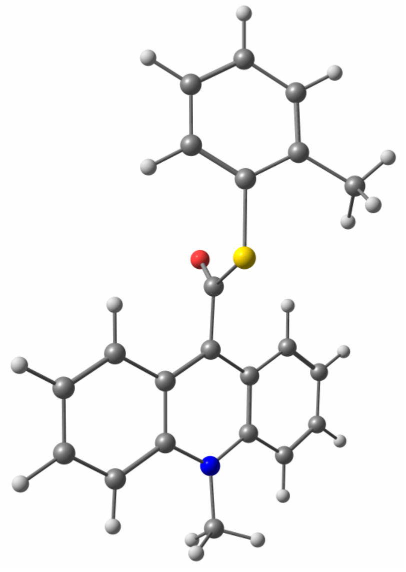

Geom.No
2= -1528.33703195 Ha

Atom	X	Y	Z
C	-0.000001659	-0.000001641	-0.000001761
C	0.000010220	0.000005736	-0.000001879
C	0.000002649	-0.000000942	0.000001543
C	-0.000000418	0.000000090	-0.000003730
C	0.000000228	0.000002438	-0.000005179
C	-0.000002371	0.000002228	-0.000002937
S	-0.000003680	-0.000006613	0.000001877
C	-0.000010072	-0.000010813	-0.000000675
O	-0.000000291	-0.000000577	0.000007371
C	-0.000002390	0.000004468	0.000001153
C	0.000028117	0.000024887	-0.000010065
C	-0.000006488	0.000005032	-0.000011450
C	-0.000000636	-0.000003012	0.000002263
N	-0.000000386	0.000003397	0.000001887
C	-0.000006189	-0.000005255	-0.000003399
C	-0.000006052	-0.000018669	0.000011085
C	0.000001457	0.000001458	-0.000000237
C	-0.000001680	0.000003044	0.000000370
C	-0.000002607	0.000003404	0.000000434
C	-0.000004176	-0.000003906	0.000006060
C	-0.000000460	-0.000000094	-0.000003598
C	0.000005441	-0.000003363	0.000001043
C	-0.000001126	-0.000007569	0.000000823
C	-0.000001870	0.000004528	-0.000005436
O	0.000002703	-0.000004212	0.000009570
O	-0.000002452	0.000007877	0.000007779
C	0.000001477	-0.000006093	-0.000000051
H	0.000000443	-0.000002330	-0.000001649
H	0.000000668	-0.000004114	-0.000002850
H	0.000001881	-0.000003844	-0.000002644
H	0.000003237	-0.000001193	-0.000001256
H	-0.000007243	0.000002113	-0.000002144
H	-0.000000232	0.000003152	0.000003382
H	-0.000000917	0.000002288	0.000002838
H	0.000000423	0.000002572	0.000000692
H	0.000000906	-0.000001437	0.000003586
H	0.000000131	-0.000000919	0.000002466
H	0.000000765	-0.000000740	0.000000127
H	0.000001154	-0.000000173	-0.000003802
H	0.000000835	-0.000000178	-0.000004649
H	-0.000000196	0.000001280	-0.000001905
H	-0.000000729	0.000002917	-0.000000632
H	0.000005081	-0.000004506	0.000004074
H	0.000000007	0.000004596	0.000002409
H	0.000000141	0.000001915	-0.000000237
H	-0.000003640	0.000002770	-0.000000669

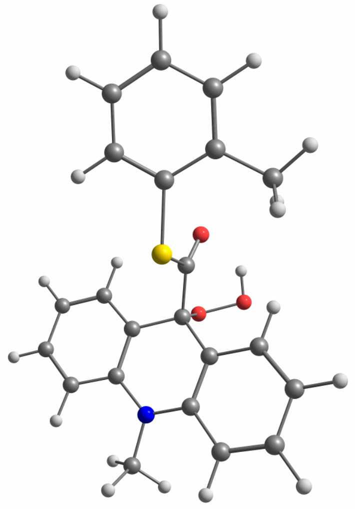

Geom.No

4=-669.207200992 Ha			
Atom	X	Y	Z
C	0.000011021	-0.000028308	-0.000055238
C	0.000021708	-0.000016526	0.000067795
C	-0.000062348	0.000035924	0.000024428
C	0.000023909	0.000012915	-0.000029362
C	0.000045696	-0.000002760	0.000039652
C	-0.000032555	0.000012306	-0.000012045
S	-0.000008608	-0.000008951	-0.000007092
C	-0.000024395	-0.000074897	0.000001617
H	0.000000874	-0.000015346	-0.000005454
H	0.000014789	-0.000001543	0.000001386
H	-0.000001454	0.000012207	0.000008666
H	-0.000006142	0.000005674	-0.000011324
H	-0.000004643	0.000025031	-0.000005254
H	0.000012199	0.000022980	-0.000013043
H	0.000009951	0.000021293	-0.000004731

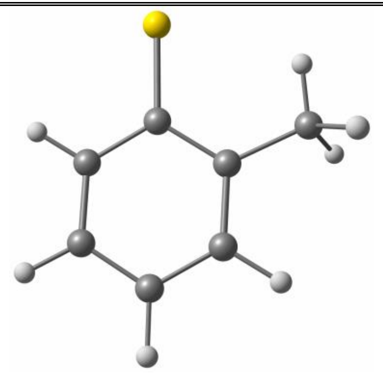

Geom.No<br>6= -1453.20811578 Ha			
Atom	X	Y	Z
C	0.000002774	0.000004833	-0.000006463
C	0.000020995	0.000002357	0.000010622
C	-0.000019444	0.000009035	0.000003014
C	0.000003367	0.000001028	-0.000007315
C	0.000008829	-0.000003311	0.000012371
C	-0.000000304	-0.000000112	0.000005423
C	-0.000008325	0.000022241	-0.000017502
C	-0.000005434	-0.000023892	0.000019730
C	-0.000002739	0.000005607	-0.000004519
N	-0.000009794	-0.000016448	-0.000005688
C	-0.000009931	-0.000001022	0.000001486
C	-0.000002965	-0.000004796	0.000001103
C	-0.000005256	-0.000004026	0.000004647
C	-0.000006232	0.000004772	-0.000001360
C	0.000021675	0.000003852	0.000011849
O	-0.000012029	0.000007469	-0.000001984
O	0.000014237	-0.000013028	0.000000664
C	0.000008974	0.000011111	0.000005653
S	-0.000012314	-0.000013023	-0.000016567
H	-0.000000899	-0.000000412	-0.000003865
H	-0.000008169	-0.000002829	-0.000003157
H	-0.000006421	-0.000001992	-0.000004141
H	-0.000001234	-0.000004146	-0.000005530
H	0.000003428	0.000000000	0.000003061
H	0.000004679	0.000003575	0.000003483
H	0.000001433	0.000001795	0.000003873
H	0.000002884	0.000001254	0.000010751
H	-0.000006256	-0.000004843	0.000005573
H	-0.000003778	-0.000000665	0.000000934
H	-0.000003304	-0.000001706	0.000002137

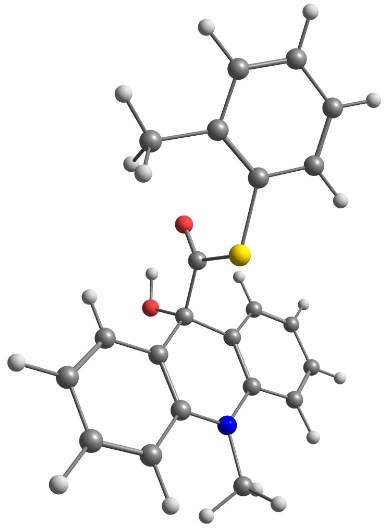

H	-0.000005967	0.000006565	-0.000004738	
C	0.000003898	-0.000000926	0.000009299	
C	0.000000225	0.000006976	-0.000009399	
C	0.000003612	0.000002512	0.000001070	
C	0.000003058	-0.000005406	-0.000002204	
C	0.000004027	-0.000000283	0.000001310	
C	-0.000002518	0.000002476	-0.000015690	
C	0.000005410	-0.000003647	0.000001629	
H	0.000004974	0.000000931	-0.000001070	
H	0.000000801	0.000000327	-0.000001038	
H	-0.000002003	0.000001287	-0.000000820	
H	-0.000001447	-0.000000190	0.000000342	
H	0.000007350	0.000001405	0.000000684	
H	0.000005337	0.000003995	-0.000001966	
H	0.000004798	0.000001300	-0.000005694	

**Geom.No 7= -1452.62798978 Ha**				
Atom	X	Y	Z	
C	0.000003754	0.000002144	-0.000003721	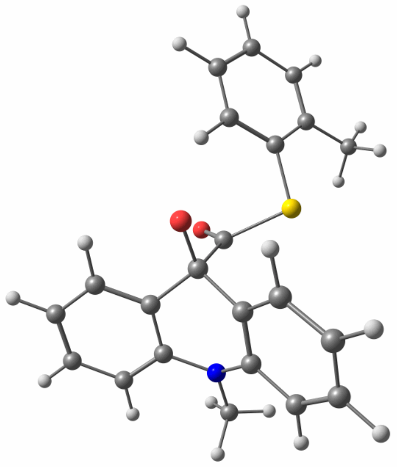
C	0.000000058	0.000002703	0.000000418	
C	0.000001638	0.000003347	-0.000003792	
C	0.000001766	0.000002707	-0.000001200	
C	0.000001338	0.000003341	0.000001176	
C	0.000000229	0.000003925	0.000001424	
C	-0.000014113	-0.000001697	0.000000110	
C	-0.000003439	0.000001274	0.000004217	
C	0.000003509	-0.000001480	0.000000977	
N	-0.000001290	0.000001797	0.000001685	
C	0.000001212	0.000002476	-0.000005257	
C	-0.000002645	-0.000002518	-0.000003481	
C	0.000002255	-0.000001932	-0.000000077	
C	0.000001379	-0.000000893	-0.000004187	
C	0.000027139	0.000033872	-0.000013596	
O	-0.000015594	-0.000026406	0.000018821	
O	0.000004128	0.000000085	0.000000438	
C	0.000001009	-0.000002417	0.000002270	
S	-0.000033803	0.000012458	-0.000028984	
C	0.000021538	-0.000017481	0.000016421	
C	0.000006751	-0.000010119	0.000008818	
C	-0.000007426	0.000005178	-0.000002857	
C	0.000004266	-0.000006709	0.000000532	
C	-0.000003715	0.000006070	0.000001259	
C	0.000012047	-0.000007281	0.000011586	
C	-0.000006619	-0.000000671	-0.000000083	
H	0.000000501	-0.000002353	-0.000003783	
H	0.000000200	-0.000000099	-0.000003484	
H	0.000000940	-0.000002078	-0.000002779	
H	0.000001338	-0.000000781	-0.000000610	
H	0.000000002	0.000002141	0.000000007	
H	0.000000033	0.000002570	0.000000545	

H	0.000001226	0.000004675	0.000000163	
H	0.000000740	0.000004294	0.000000438	
H	-0.000001300	0.000000824	0.000002065	
H	-0.000001244	-0.000000755	0.000001369	
H	-0.000000114	0.000000991	-0.000000207	
H	-0.000001744	-0.000003569	0.000001297	
H	-0.000000933	-0.000000352	0.000000953	
H	0.000000584	0.000000832	0.000000388	
H	-0.000003070	0.000002963	-0.000001206	
H	-0.000002901	-0.000005429	0.000001132	
H	0.000002037	-0.000004252	-0.000000535	
H	-0.000001665	-0.000001397	0.000001331	

Geom.No
VIII-TS=-1452.62353583 Ha

Atom	X	Y	Z	
C	0.000003369	-0.000001998	0.000003806	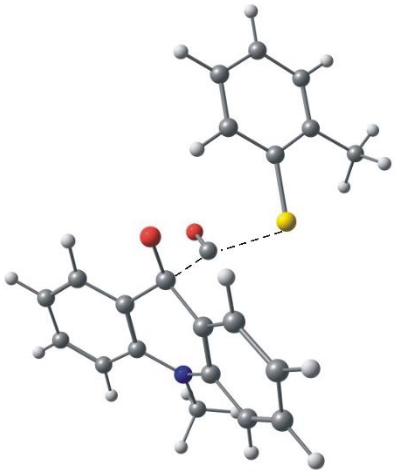
C	0.000000171	0.000004646	-0.000000894	
C	0.000004086	-0.000002693	0.000001345	
C	-0.000000805	0.000000117	0.000001847	
C	0.000001535	-0.000000202	0.000000754	
C	0.000002283	-0.000002893	0.000000169	
S	0.001230593	0.004382469	0.004733161	
C	-0.001472059	-0.002433729	-0.008583978	
O	-0.000002120	-0.000000973	0.000000319	
C	0.000000782	-0.000003199	0.000000490	
C	0.000237926	-0.001949670	0.003852321	
C	0.000000154	0.000001798	-0.000000885	
C	-0.000001883	0.000001468	-0.000001075	
N	-0.000001435	-0.000000859	-0.000000637	
C	0.000000341	0.000001523	-0.000000825	
C	0.000000814	0.000001520	0.000000340	
C	-0.000002871	0.000001319	-0.000001997	
C	-0.000001094	0.000002414	-0.000001880	
C	-0.000003068	0.000004010	-0.000000735	
C	-0.000001265	0.000002150	-0.000001058	
C	-0.000000688	-0.000001838	0.000000068	
C	0.000000716	-0.000000214	0.000000629	
C	0.000001001	-0.000000216	0.000001207	
C	0.000001206	-0.000001501	0.000000817	
O	0.000003303	0.000000400	0.000000577	
C	-0.000001160	-0.000000691	-0.000002529	
H	0.000001926	-0.000000520	0.000002270	
H	0.000001015	0.000001045	0.000002186	
H	0.000000982	-0.000001104	0.000000913	
H	-0.000000155	-0.000000980	-0.000000847	
H	-0.000002281	0.000001641	-0.000002744	
H	-0.000001151	0.000002858	0.000000178	
H	-0.000002399	0.000003559	-0.000001185	
H	-0.000003551	0.000003067	-0.000002573	
H	-0.000001979	-0.000000360	-0.000002612	

H	-0.000001069	-0.000001221	-0.000001742
H	-0.000001666	0.000000046	-0.000001988
H	0.000001429	-0.000002190	0.000001264
H	0.000001301	-0.000000432	0.000001665
H	0.000001182	0.000000981	0.000002136
H	0.000000709	0.000001397	0.000001253
H	0.000001766	-0.000003790	0.000000042
H	0.000002309	-0.000003702	0.000000589
H	0.000001800	-0.000003455	-0.000000162

R_2=C(CH_3)$_3$ R_4=H R_6=H, $R_{2'}$=H

Geom.No
1=-1495.11262934 Ha

Atom	X	Y	Z
C	-0.000000004	-0.000000345	-0.000001122
C	-0.000001229	0.000000091	0.000001157
C	0.000001940	0.000003857	0.000003758
C	-0.000000122	0.000000695	0.000001329
C	0.000000346	-0.000000106	0.000001630
C	0.000000762	0.000001873	0.000001698
C	0.000009170	0.000006593	0.000005191
C	-0.000001088	0.000001313	0.000000756
C	-0.000005025	-0.000003213	0.000001637
N	0.000001571	0.000000642	-0.000003743
C	0.000000460	-0.000000225	0.000003225
C	-0.000001042	-0.000000263	-0.000000025
C	-0.000000815	-0.000000340	-0.000001298
C	-0.000001981	-0.000001691	0.000000352
C	-0.000020851	0.000001614	-0.000005726
O	0.000002027	0.000002675	-0.000002415
C	0.000000385	-0.000000583	0.000003343
S	0.000045903	-0.000009188	0.000015879
C	-0.000031224	0.000007715	-0.000014002
C	0.000006520	0.000000175	0.000001088
C	-0.000003678	-0.000001353	-0.000004911
C	-0.000001691	-0.000004882	-0.000001612
C	0.000009083	-0.000000928	0.000000648
C	-0.000006290	-0.000010170	-0.000009890
C	0.000022107	-0.000009437	-0.000019386
H	-0.000001085	0.000000236	-0.000000080
H	-0.000002314	-0.000000770	0.000000343
H	-0.000001301	-0.000000904	-0.000000240
H	-0.000000107	-0.000000465	-0.000000691
H	0.000000410	-0.000000022	0.000001356
H	0.000001788	0.000000356	0.000000946
H	0.000000880	0.000002282	0.000001751
H	-0.000000211	0.000001718	-0.000000041
H	-0.000003112	0.000003090	0.000001520
H	-0.000001116	0.000000771	0.000002047
H	-0.000000061	-0.000000966	0.000003143
H	0.000000339	0.000002294	-0.000003932

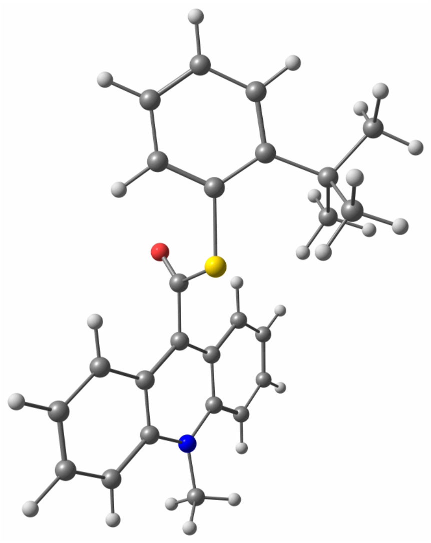

H	0.000001472	0.000000862	-0.000003088
H	0.000001619	-0.000001330	-0.000000862
H	-0.000001132	-0.000002124	-0.000000076
C	-0.000005777	-0.000003569	0.000009329
C	-0.000001977	0.000009424	0.000015396
C	-0.000027059	0.000013794	0.000008384
H	0.000002553	-0.000001697	-0.000005361
H	-0.000000349	0.000000634	-0.000003640
H	-0.000000495	-0.000000801	0.000000187
H	0.000005313	-0.000000414	0.000002486
H	0.000002375	-0.000005989	-0.000000019
H	0.000004435	0.000000845	-0.000003158
H	0.000001502	-0.000002070	-0.000000334
H	-0.000000583	0.000000967	-0.000003978
H	-0.000001241	-0.000000670	0.000001052

Geom.No
2=-1646.27261227 Ha

Atom	X	Y	Z
C	-0.000003052	0.000000686	0.000000393
C	0.000000664	0.000001204	0.000000258
C	0.000001399	0.000000852	-0.000000519
C	0.000000559	0.000000253	-0.000002316
C	-0.000002042	0.000000395	-0.000002431
C	0.000001439	0.000000125	-0.000001511
C	0.000003130	-0.000009291	-0.000001811
C	-0.000002536	0.000000942	-0.000000585
C	0.000001051	0.000000789	0.000000078
N	0.000001295	-0.000000576	-0.000001253
C	0.000000928	-0.000000389	0.000001272
C	0.000001769	0.000000496	0.000000342
C	0.000001798	0.000000486	-0.000000768
C	0.000001271	-0.000000607	0.000000725
C	-0.000003838	0.000014081	-0.000001273
O	0.000002538	-0.000004372	0.000003901
O	0.000000778	0.000002652	-0.000004172
O	-0.000004489	-0.000001282	0.000001730
C	0.000001404	0.000001019	-0.000001164
S	0.000002633	-0.000009040	-0.000000505
C	0.000004498	-0.000001976	0.000003371
C	-0.000000721	0.000000482	-0.000000806
C	-0.000001630	0.000000521	0.000002515
C	-0.000001741	-0.000001444	0.000001581
C	-0.000000082	-0.000000041	0.000001744
C	-0.000002568	-0.000000337	0.000002975
C	0.000000406	-0.000000266	-0.000001004
C	-0.000001186	-0.000000561	-0.000000911
C	-0.000000496	0.000000354	-0.000000004
C	-0.000001713	-0.000000298	-0.000000729
H	-0.000000056	0.000000250	0.000001844
H	-0.000000207	0.000000051	-0.000002224

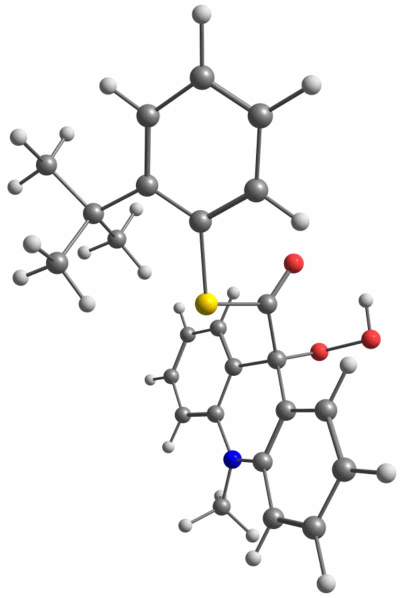

H	-0.000000788	0.000000002	-0.000001934
H	-0.000001439	0.000000145	-0.000000944
H	-0.000000614	-0.000000451	0.000000140
H	0.000000021	0.000000002	-0.000000112
H	0.000001857	-0.000000160	0.000000644
H	0.000002384	0.000000146	-0.000000177
H	0.000001376	0.000000210	-0.000001671
H	0.000000578	0.000000729	-0.000001496
H	0.000001488	0.000000111	-0.000001907
H	0.000001368	0.000000492	-0.000001960
H	-0.000000104	-0.000000153	0.000002510
H	-0.000000528	-0.000000521	0.000001731
H	-0.000001450	0.000000351	0.000001762
H	0.000000529	0.000001364	0.000001234
H	-0.000000695	-0.000000228	0.000000366
H	-0.000000916	-0.000000293	0.000000326
H	-0.000000710	-0.000000649	0.000000396
H	-0.000000846	0.000000295	-0.000000478
H	-0.000000295	0.000000306	0.000000766
H	-0.000001316	0.000000107	0.000001296
H	-0.000000285	0.000000098	-0.000000540
H	-0.000001403	0.000002957	0.000000523
H	0.000000586	-0.000000019	0.000000786

Geom.No
4=-787.147229284 Ha

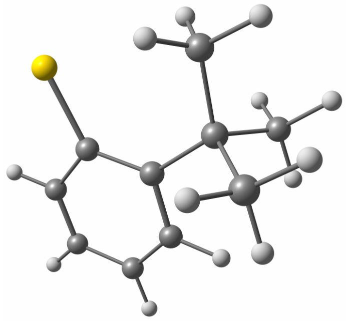

Atom	X	Y	Z
C	0.000008649	0.000005330	0.000007881
C	-0.000010122	-0.000016144	-0.000008874
C	0.000017132	0.000006096	-0.000001545
C	-0.000000944	0.000011562	-0.000002122
C	0.000014806	-0.000030185	0.000000648
C	-0.000010990	0.000004899	-0.000008596
S	-0.000010413	0.000009150	-0.000011672
C	0.000005153	-0.000012036	0.000017453
C	-0.000003731	-0.000001685	0.000008884
C	0.000018230	0.000031944	-0.000016054
C	-0.000010897	0.000011795	-0.000000344
H	-0.000005132	-0.000001515	-0.000005057
H	0.000001547	0.000006660	0.000000126
H	0.000001108	0.000000368	-0.000000425
H	0.000003139	0.000000759	-0.000007233
H	0.000000854	-0.000001135	0.000005455
H	-0.000006174	-0.000006828	0.000005473
H	0.000007926	-0.000001990	0.000003040
H	-0.000006063	-0.000001229	-0.000004549
H	-0.000008477	-0.000001235	0.000010761
H	0.000001123	-0.000000098	0.000000194
H	-0.000002082	-0.000005289	0.000008594
H	-0.000002062	-0.000004844	0.000001174
H	-0.000002580	-0.000004350	-0.000003213

Geom.No
6= -1571.14382109 Ha

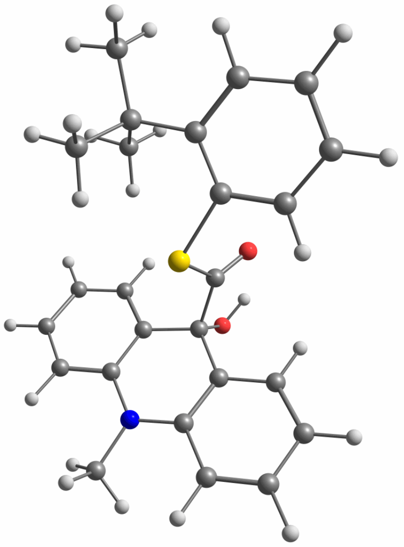

Atom	X	Y	Z
C	0.000001809	-0.000000598	0.000004884
C	-0.000001341	0.000000127	0.000003159
C	-0.000000609	-0.000003775	0.000003125
C	-0.000002682	-0.000001969	0.000006421
C	-0.000000415	-0.000002813	0.000004656
C	0.000002084	-0.000003331	0.000004818
C	-0.000004018	-0.000004783	0.000008907
C	0.000003536	-0.000002945	-0.000008233
C	-0.000003416	-0.000000189	0.000001860
N	-0.000004273	-0.000001622	0.000004402
C	-0.000004362	-0.000000399	0.000001383
C	-0.000002494	-0.000001325	0.000001317
C	-0.000001492	0.000000922	-0.000002501
C	-0.000001422	-0.000000253	0.000002329
C	0.000010289	0.000007127	-0.000001273
O	0.000003484	-0.000000572	-0.000000878
O	0.000004456	0.000001452	0.000000990
C	-0.000004185	-0.000003090	0.000004475
S	-0.000002909	-0.000004999	0.000002504
C	-0.000000311	0.000002752	-0.000004472
C	0.000004169	0.000002005	-0.000003642
C	0.000002803	0.000002843	-0.000006601
C	0.000003537	0.000004393	-0.000006061
C	0.000002918	0.000002169	-0.000004623
C	0.000001188	0.000001724	-0.000004284
C	0.000003287	0.000001435	-0.000002847
H	-0.000005202	-0.000001322	0.000001997
H	-0.000004864	0.000000086	-0.000000219
H	-0.000001378	0.000000804	-0.000001639
H	0.000001627	0.000000632	-0.000001036
H	0.000003995	-0.000001838	0.000002717
H	0.000002846	-0.000002521	0.000005302
H	-0.000000876	-0.000003740	0.000006838
H	-0.000003167	-0.000003156	0.000006282
H	-0.000005686	-0.000003083	0.000004679
H	-0.000005941	-0.000002384	0.000003507
H	-0.000003771	-0.000003183	0.000005356
H	0.000002450	0.000003227	-0.000005228
H	0.000004456	0.000004159	-0.000007472
H	0.000004540	0.000004046	-0.000006893
H	0.000001894	0.000002605	-0.000004351
H	0.000004510	-0.000000458	0.000001957
C	0.000000546	0.000002716	-0.000002348
C	-0.000001342	-0.000001343	0.000001267
C	-0.000002930	0.000001291	-0.000003231
H	0.000000215	0.000001251	-0.000002824
H	0.000000236	0.000002288	-0.000004066

H	0.000001881	0.000001758	-0.000003024
H	-0.000002406	0.000001215	-0.000001644
H	-0.000002983	0.000001058	-0.000001569
H	-0.000002619	0.000001539	-0.000004010
H	0.000000497	-0.000000116	0.000000382
H	0.000003070	0.000000352	-0.000000471
H	0.000000773	-0.000000169	-0.000000069

Geom.No
7=-1570.56610072 Ha

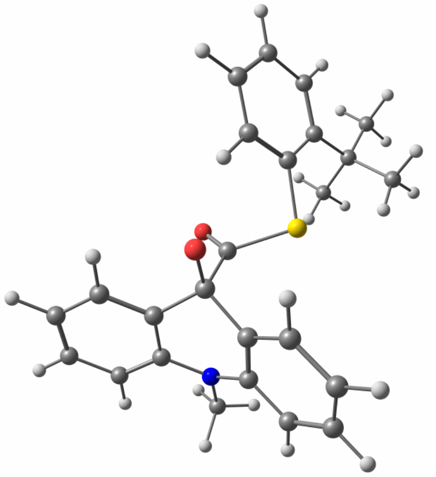

Atom	X	Y	Z
C	0.000003652	0.000000242	0.000000833
C	-0.000007756	-0.000000553	-0.000000725
C	0.000006657	0.000001098	0.000000273
C	-0.000000323	-0.000000056	0.000005545
C	0.000001209	-0.000000036	0.000000412
C	0.000004623	-0.000000212	0.000001843
C	-0.000000269	-0.000010396	0.000001172
C	-0.000014860	-0.000000675	-0.000000420
C	0.000008062	-0.000001209	0.000002563
N	-0.000003364	-0.000003228	-0.000001046
C	0.000000053	0.000004390	-0.000003483
C	-0.000009795	-0.000003975	-0.000001191
C	0.000002915	-0.000002458	0.000003733
C	0.000001241	0.000003756	-0.000004632
C	0.000000997	0.000016471	0.000002477
O	-0.000001334	-0.000005799	0.000012168
O	0.000002949	-0.000007746	-0.000001513
C	0.000000350	-0.000000526	0.000002744
S	-0.000007388	0.000019754	-0.000011962
C	-0.000000161	-0.000000753	-0.000017209
C	0.000023985	-0.000023130	0.000006699
C	-0.000017600	0.000023155	0.000001053
C	0.000012804	-0.000016234	-0.000010617
C	-0.000031291	0.000030314	-0.000014291
C	0.000043255	0.000001891	0.000085178
C	0.000001057	-0.000038826	-0.000039172
H	-0.000002916	-0.000000316	-0.000002185
H	-0.000002040	0.000002054	-0.000001090
H	-0.000002669	-0.000000189	0.000000701
H	-0.000002538	-0.000001796	0.000002529
H	0.000001380	-0.000001843	0.000003040
H	0.000002079	0.000000492	0.000000923
H	0.000002679	0.000000861	0.000001760
H	0.000002587	-0.000000611	0.000003027
H	-0.000000336	-0.000001892	0.000002877
H	-0.000001225	-0.000001076	0.000002988
H	-0.000001713	-0.000001923	0.000003143
H	0.000003558	-0.000011377	-0.000013987
H	0.000000302	0.000002076	-0.000004728
H	0.000003240	0.000002585	-0.000004858

H	-0.000001342	0.000005276	-0.000005606
C	-0.000011382	0.000019184	0.000011509
C	0.000009768	0.000021721	-0.000014223
C	-0.000020448	-0.000008465	0.000007248
H	-0.000000980	0.000002270	-0.000003619
H	0.000002712	-0.000001651	-0.000001289
H	0.000005586	-0.000001508	-0.000002817
H	-0.000001601	-0.000001812	-0.000002305
H	0.000001722	-0.000001896	0.000001054
H	0.000002893	-0.000000248	-0.000001276
H	-0.000001561	-0.000002267	-0.000002314
H	-0.000002855	-0.000001265	0.000001388
H	-0.000004566	-0.000001645	-0.000002322

Geom.No
VIII–TS= -1570.56087897 Ha

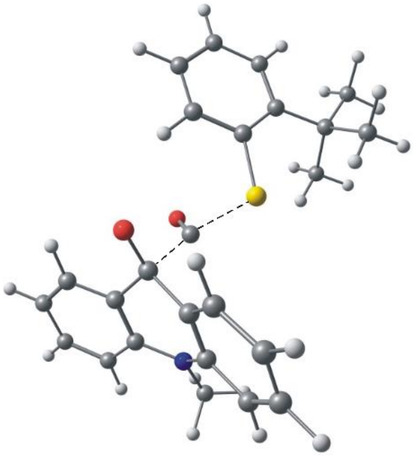

Atom	X	Y	Z
C	-0.000003622	-0.000006641	-0.000000168
C	0.000019821	-0.000009225	-0.000004781
C	-0.000015544	0.000011085	0.000004173
C	0.000004688	-0.000002774	-0.000006836
C	-0.000001650	-0.000004869	-0.000000846
C	-0.000002214	0.000001237	0.000003469
S	-0.000772162	-0.001437275	-0.000880281
C	0.002649372	0.000825243	0.001848257
O	0.000008700	-0.000012452	-0.000003259
C	0.000002766	-0.000001525	-0.000005172
C	-0.000001706	-0.000002880	0.000001630
C	0.000001158	-0.000003711	0.000002729
C	0.000000310	-0.000003607	-0.000002434
C	-0.001902272	0.000606105	-0.000936724
C	0.000009533	0.000004603	-0.000015307
C	0.000001717	-0.000004999	0.000004924
N	-0.000012493	0.000004702	-0.000010625
C	0.000000894	-0.000005721	0.000005501
C	0.000003980	0.000014015	0.000001244
C	-0.000001642	-0.000000856	0.000001688
C	0.000001698	-0.000000078	0.000002589
C	-0.000000395	-0.000001085	0.000000890
C	-0.000000555	0.000002906	0.000003713
C	-0.000002716	0.000000330	-0.000001042
C	-0.000001856	-0.000000424	0.000002762
C	0.000001636	0.000006060	0.000001514
C	-0.000003744	-0.000006416	-0.000004274
O	0.000014018	0.000019743	-0.000013571
C	0.000004502	0.000001916	-0.000000938
H	0.000000190	0.000001639	0.000002444
H	0.000000497	0.000000915	0.000001941
H	0.000000924	0.000001141	0.000001329
H	0.000000673	-0.000001834	0.000001623
H	-0.000001382	0.000001856	0.000001348

H	-0.000001840	0.000001233	0.000001854
H	-0.000003251	0.000001824	-0.000002369
H	-0.000001757	0.000002083	-0.000003103
H	-0.000000915	-0.000001352	-0.000001159
H	-0.000000459	0.000001184	0.000001476
H	-0.000000478	-0.000000172	-0.000000851
H	-0.000000295	-0.000002037	-0.000002132
H	-0.000002838	0.000001799	-0.000002367
H	0.000001650	0.000001558	-0.000001867
H	0.000003995	0.000007582	0.000003161
H	-0.000000232	-0.000003782	-0.000001948
H	-0.000000096	0.000004604	0.000002868
H	0.000002931	-0.000001222	-0.000001504
H	-0.000000356	-0.000003737	0.000002628
H	-0.000001430	-0.000000904	-0.000002104
H	-0.000001971	-0.000002265	0.000001771
H	0.000001647	-0.000001939	-0.000001464
H	0.000001137	-0.000001019	0.000000075
H	0.000001430	-0.000000559	-0.000000474

R_2=H R_4=H, R_6=H $R_{2'}$=OCH_3

Geom.No
1= -1452.38560725 Ha

Atom	X	Y	Z
C	-0.000000284	0.000002939	-0.000001184
C	0.000004205	0.000001314	0.000009880
C	-0.000001822	-0.000005659	-0.000009613
C	0.000000878	0.000000143	-0.000000047
C	0.000000270	-0.000001721	0.000003333
C	0.000000529	0.000004462	-0.000012691
C	0.000000446	0.000005513	-0.000019026
C	-0.000009083	-0.000005046	0.000002661
C	0.000015520	0.000000770	-0.000009907
N	-0.000006771	-0.000001343	0.000011484
C	-0.000004478	-0.000000020	0.000002746
C	0.000003759	-0.000000622	-0.000001093
C	-0.000003202	-0.000002047	-0.000000799
C	0.000004527	0.000001748	0.000000000
C	-0.000058408	0.000082221	0.000014781
O	-0.000009545	0.000000478	0.000011688
C	0.000001038	-0.000001740	-0.000011472
S	0.000100222	-0.000085192	0.000045540
C	-0.000056243	-0.000004308	-0.000062746
C	0.000005484	0.000004253	0.000003257
C	-0.000000598	0.000003320	-0.000000728
C	-0.000002419	0.000000961	0.000003356
C	-0.000001257	0.000000048	0.000006505
C	0.000014529	0.000007802	0.000001888
H	-0.000002280	0.000001701	0.000002472
H	0.000000969	-0.000001833	-0.000000124
H	0.000002638	-0.000002640	-0.000000705

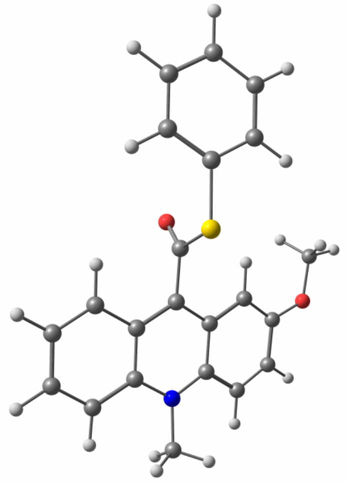

H	0.000001602	-0.000000669	0.000000172
H	-0.000000336	-0.000001694	-0.000000574
H	0.000000104	-0.000000125	-0.000001254
O	-0.000006982	0.000002877	0.000004369
H	-0.000000684	-0.000004329	-0.000002118
H	0.000001340	-0.000005109	0.000001222
H	0.000003267	-0.000005165	0.000000599
H	0.000001184	-0.000004264	-0.000000942
H	-0.000000013	-0.000001064	-0.000003282
H	-0.000000227	0.000004180	0.000001808
H	0.000000630	0.000004886	0.000001871
H	-0.000001240	0.000003760	0.000003051
H	0.000005917	0.000001692	0.000006528
C	0.000001941	-0.000002985	-0.000000627
H	-0.000001726	0.000000768	-0.000000322
H	-0.000002702	0.000000517	-0.000003444
H	-0.000000699	0.000001225	0.000003484

Geom.No
2= -1603.53970619 Ha

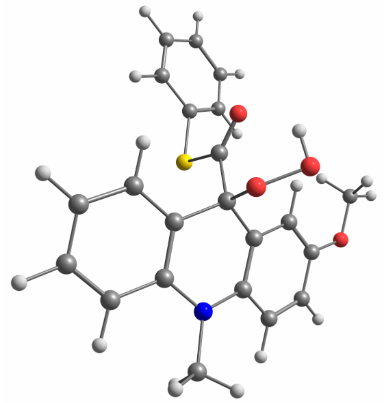

Atom	X	Y	Z
C	0.000000250	-0.000001726	-0.000000176
C	-0.000017449	0.000000150	0.000009320
C	0.000000400	0.000000422	-0.000001451
C	-0.000000706	-0.000000193	-0.000000226
C	-0.000000702	-0.000000522	-0.000001715
C	-0.000000200	-0.000001197	-0.000000254
S	0.000024907	0.000025798	-0.000014221
C	-0.000021190	-0.000031978	0.000009489
O	0.000003856	0.000002759	0.000001136
C	0.000018192	0.000001552	-0.000008832
C	-0.000008136	0.000003123	0.000007561
C	0.000003318	-0.000001076	-0.000004118
N	-0.000000275	-0.000000151	0.000003081
C	-0.000000997	0.000002650	0.000000307
C	0.000002610	0.000000190	0.000001942
C	-0.000001852	-0.000000358	-0.000001037
C	0.000000035	0.000000159	0.000000185
C	0.000000515	-0.000001739	0.000001765
C	-0.000000422	-0.000003842	-0.000001859
C	0.000000779	0.000001896	0.000000038
C	0.000001866	0.000002114	-0.000000375
C	-0.000000217	0.000002384	0.000001739
C	-0.000001430	0.000001866	-0.000000086
O	-0.000001144	-0.000003624	-0.000001860
C	-0.000001793	-0.000002556	-0.000001657
O	-0.000003260	-0.000001356	0.000001234
O	0.000003668	0.000000668	0.000001253
C	-0.000000597	0.000002556	-0.000002302
H	-0.000002009	-0.000001240	0.000000237
H	0.000000731	0.000001298	0.000000339

H	0.000000068	-0.000000964	-0.000000828	
H	0.000001230	-0.000001191	0.000002098	
H	0.000000579	0.000001744	0.000002234	
H	0.000000672	0.000002892	0.000000186	
H	0.000000177	0.000003313	0.000000070	
H	0.000000648	0.000002475	-0.000000510	
H	0.000000032	-0.000000465	-0.000000835	
H	0.000000152	0.000002490	0.000000004	
H	0.000000087	0.000001795	-0.000000550	
H	-0.000000208	0.000001030	-0.000001460	
H	-0.000000115	0.000000193	-0.000001366	
H	-0.000000275	-0.000001073	-0.000000448	
H	-0.000000496	-0.000002032	-0.000000308	
H	-0.000000873	0.000000016	0.000000885	
H	-0.000000133	-0.000003115	0.000000268	
H	-0.000000213	-0.000002784	0.000000479	

Geom.No<br>3= -973.086548205 Ha				
Atom	X	Y	Z	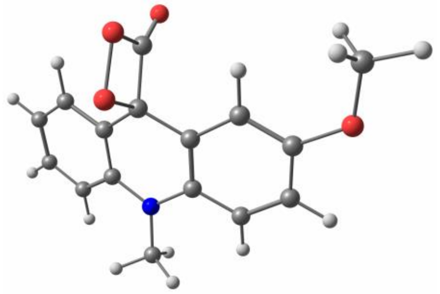
C	-0.000004306	0.000000010	0.000006200	
C	0.000003891	-0.000004276	0.000014364	
C	-0.000001340	0.000004846	0.000000215	
C	0.000001352	0.000002905	0.000002583	
C	0.000005219	0.000000421	-0.000005713	
C	0.000002578	-0.000003021	0.000001896	
N	-0.000001067	0.000006687	0.000006159	
C	-0.000013214	0.000003982	-0.000000342	
C	-0.000002407	0.000001609	-0.000011675	
C	0.000002159	-0.000021287	0.000001886	
C	-0.000001291	0.000001525	-0.000001063	
C	-0.000001260	-0.000000200	-0.000003872	
C	-0.000002178	-0.000000927	-0.000007955	
C	0.000016135	-0.000006360	-0.000006705	
C	0.000000808	0.000014038	0.000002200	
O	0.000000782	-0.000005146	-0.000001585	
O	-0.000006221	0.000011432	-0.000000031	
C	0.000001290	-0.000004606	-0.000000090	
O	0.000001465	0.000001118	-0.000000738	
C	-0.000001751	-0.000001303	0.000000978	
O	-0.000001790	-0.000000069	-0.000001275	
H	-0.000000984	-0.000001510	-0.000001395	
H	-0.000001385	-0.000001182	-0.000000580	
H	-0.000000825	0.000000587	-0.000000933	
H	-0.000000806	0.000000800	0.000001249	
H	-0.000000913	-0.000000378	0.000000822	
H	0.000003399	-0.000000261	-0.000000075	
H	0.000000477	-0.000001116	0.000000405	
H	0.000000214	-0.000002211	-0.000000786	
H	-0.000000334	-0.000001817	-0.000001769	
H	0.000000441	-0.000000004	0.000000930	

H	0.000000124	0.000002368	0.000001500	
H	0.000001626	0.000001733	0.000002494	
H	0.000000113	0.000001611		

Geom.No
III-TS=-973.063867097 Ha

Atom	X	Y	Z	
C	0.000664833	-	0.001330079	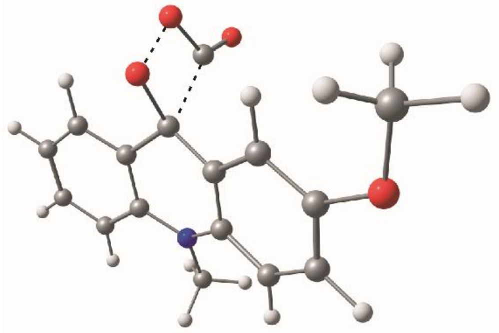
C	0.002306026	0.000072329	-	
C	0.000215488	0.000048687	0.002134878	
C	-	0.000535347	-	
C	0.001458730	-	0.001100421	
C	-	0.000145943	0.000155326	
C	0.000699907	-	-	
C	0.000937454	0.000052075	0.000692557	
C	0.026479276	0.000028674	-	
N	-	0.036032754	0.000243855	
C	0.001159846	0.000145004	0.020724559	
C	-	-	0.001070405	
C	0.000474069	0.000795024	0.002864651	
C	-	0.001190445	-	
C	0.002749128	0.000177828	0.001117836	
O	0.001170577	-	-	
O	0.000994435	0.000085828	0.000155687	
C	-	-	0.001039255	
O	0.001486393	0.000041806	-	
O	-	0.000104888	0.000984938	
C	0.000219288	-	-	
H	0.002891136	0.000282403	0.001186225	
H	-	-	0.001632575	
H	0.001520544	0.000423603	-	
H	-	-	0.001037199	
H	0.001940058	0.000716141	-	
H	-	-	0.001314950	
H	0.019883018	0.025225166	-	
H	-	-	0.015024211	
H	0.003070121	0.009912026	-	
H	0.002148858	0.000005508	0.003436639	
H	-	0.000001766	0.000694253	
H	0.002966480	-	-	
H	-	0.000165712	0.001037748	
	0.000327831	0.000052357	0.000458761	
	0.000515728	-	0.000037303	
	-	0.000164847	-	
	0.000063846	-	0.000061914	
	-	0.000171278	-	
	0.000072488	0.000000848	0.000034080	
	0.000196130	0.000030741	-	
	-	0.000007371	0.000471339	
	0.000042319	-	-	
		0.000003944	0.000008997	

	-<br>0.000058044<br>-<br>0.000106645<br>0.000137235<br>-<br>0.000501716<br>-<br>0.000105491<br>0.000135585<br>0.000113200	0.000079330<br>-<br>0.000210436<br>0.000014793<br>0.000134419<br>-<br>0.000122199	0.000113513<br>0.000251819<br>-<br>0.000207346<br>-<br>0.000349135<br>-<br>0.000219221<br>0.000207619<br>0.000239057	

Geom.No 5=-784.655818845 Ha				
Atom	X	Y	Z	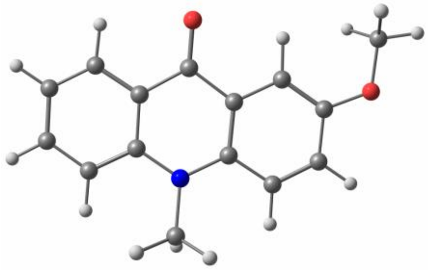
C	0.000007862	-0.000005242	-0.000007303	
C	0.000004159	0.000002930	0.000013759	
C	-0.000025539	0.000011120	0.000005921	
C	0.000023113	-0.000015639	-0.000005301	
C	0.000010356	0.000024071	-0.000000500	
C	-0.000007082	-0.000020153	0.000004608	
C	0.000027804	-0.000007753	-0.000011490	
C	-0.000014712	0.000020703	0.000005798	
C	-0.000000755	0.000001366	-0.000027723	
N	0.000000342	0.000007066	0.000033169	
C	-0.000022888	-0.000031164	0.000015981	
C	0.000018640	0.000014451	0.000015415	
C	-0.000008606	0.000008796	-0.000021853	
C	-0.000003631	-0.000010849	0.000004324	
O	-0.000005783	-0.000030022	-0.000020465	
C	0.000008184	0.000013990	0.000022382	
C	-0.000008464	-0.000014348	-0.000010526	
H	0.000002120	0.000012142	0.000001910	
H	0.000005144	0.000008364	0.000001270	
H	-0.000001555	-0.000009843	-0.000006107	
H	-0.000002811	0.000004035	0.000000492	
H	-0.000003613	0.000006419	-0.000008324	
H	-0.000001289	0.000004954	0.000000015	
H	-0.000005700	0.000011811	-0.000007599	
H	0.000002337	0.000002128	0.000004331	
H	0.000001798	0.000004123	0.000002919	
H	0.000004314	0.000005096	0.000012275	
H	-0.000000501	0.000001458	0.000001353	
H	-0.000003868	-0.000006806	-0.000004256	

H	-0.000001934	-0.000010276	0.000001259	
H	0.000002555	-0.000002930	-0.000015734	

Geom.No
5*–S1= -784.537026295 Ha

Atom	X	Y	Z	
C	-0.000014247	0.000003349	-0.000000569	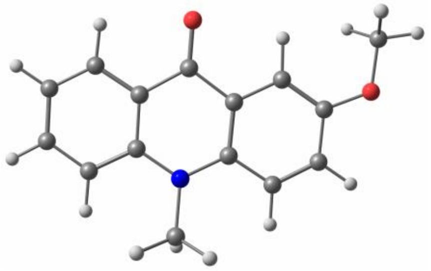
C	0.000001418	0.000003113	0.000008734	
C	-0.000001261	0.000003136	-0.000009659	
C	0.000000035	-0.000001941	0.000015778	
C	-0.000004948	-0.000000756	-0.000018941	
C	0.000014012	0.000006416	-0.000007865	
C	-0.000005961	-0.000012697	0.000004496	
C	0.000006652	0.000010405	-0.000005840	
C	-0.000003337	0.000007307	0.000012585	
N	0.000000841	-0.000013732	-0.000015142	
C	0.000009698	-0.000001164	0.000002131	
C	-0.000012290	-0.000002622	0.000000883	
C	-0.000002234	-0.000007141	0.000009284	
C	-0.000000728	0.000000991	-0.000005020	
O	0.000002040	0.000000982	-0.000000483	
C	0.000003606	-0.000000379	0.000002576	
C	-0.000001583	0.000003063	0.000003260	
H	0.000001660	0.000002603	-0.000000185	
H	-0.000001803	-0.000001798	-0.000000535	
H	-0.000001241	-0.000002059	-0.000001650	
H	-0.000000351	-0.000001916	-0.000001566	
H	0.000001947	-0.000005877	-0.000001235	
H	0.000000353	0.000003245	0.000001576	
H	-0.000000219	0.000002463	0.000001481	
H	-0.000001805	0.000000628	-0.000000064	
H	0.000000781	0.000004048	0.000003691	
H	-0.000000816	0.000003215	0.000002376	
H	0.000001805	-0.000001800	-0.000000210	
H	0.000000811	-0.000002278	-0.000001522	
H	0.000002110	-0.000002221	0.000000074	
O	0.000005056	0.000003418	0.000001563	

Geom.No
5*–T1=-784.569801133 Ha

Atom	X	Y	Z	
C	-0.000004567	0.000000790	-0.000000533	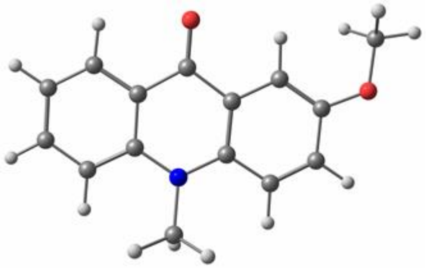
C	0.000010252	0.000000232	0.000002182	
C	-0.000003662	0.000001559	-0.000014026	
C	-0.000006748	-0.000002571	0.000008818	
C	0.000001986	0.000001025	-0.000011263	
C	0.000005059	-0.000000050	0.000009372	
C	0.000023679	-0.000003011	0.000010361	
C	-0.000008404	-0.000005956	0.000031072	
C	0.000015077	0.000011590	-0.000039440	

N	-0.000016267	-0.000002000	0.000019124
C	-0.000008169	0.000000339	-0.000021450
C	0.000005981	0.000000727	0.000006033
C	0.000012454	0.000003780	-0.000010400
C	-0.000006082	-0.000003591	0.000021223
O	-0.000007241	-0.000003379	0.000003614
C	0.000004078	-0.000000340	-0.000003346
C	0.000005511	0.000000385	0.000000120
H	0.000002588	0.000002110	-0.000002085
H	-0.000003392	0.000000562	-0.000001878
H	-0.000001026	-0.000001255	0.000001443
H	0.000001253	-0.000002128	-0.000002431
H	0.000001965	-0.000003042	0.000004069
H	-0.000003555	0.000001275	-0.000001544
H	-0.000001134	0.000001659	0.000001697
H	-0.000000414	0.000002792	0.000001934
H	-0.000001777	0.000002931	0.000000521
H	-0.000000705	0.000002357	0.000000341
H	0.000000439	-0.000001933	-0.000000458
H	0.000000235	-0.000002707	-0.000001080
H	0.000001433	-0.000001699	-0.000000476
O	-0.000018850	-0.000000452	-0.000011512

Geom.No
6=-1528.41076993 Ha

Atom	X	Y	Z
C	-0.000004899	0.000000741	0.000001605
C	-0.000008815	0.000001115	-0.000003032
C	0.000007934	0.000002174	-0.000003997
C	-0.000006267	0.000002581	0.000004290
C	-0.000009067	0.000001931	-0.000010710
C	0.000003029	0.000002090	-0.000004455
S	0.000000065	-0.000003096	-0.000002738
C	-0.000000293	0.000005009	0.000000647
O	-0.000003679	-0.000001725	-0.000001394
H	-0.000000082	0.000000376	0.000001281
C	-0.000001997	-0.000004192	0.000003871
C	0.000001095	0.000000032	-0.000001806
C	0.000001681	0.000001547	0.000001447
N	0.000002644	-0.000004487	-0.000000331
C	0.000000783	-0.000000816	0.000003143
C	-0.000000153	-0.000001674	0.000001240
C	0.000004187	-0.000000694	0.000000975
C	0.000003578	0.000000202	-0.000000190
C	0.000003028	0.000002103	-0.000001356
C	0.000000004	0.000000755	-0.000000673
C	0.000000007	-0.000002888	0.000003343
C	-0.000001344	-0.000003698	0.000003898
C	-0.000002169	-0.000002740	0.000003175
C	-0.000002543	-0.000002251	0.000001355
O	0.000002938	0.000002070	-0.000001989

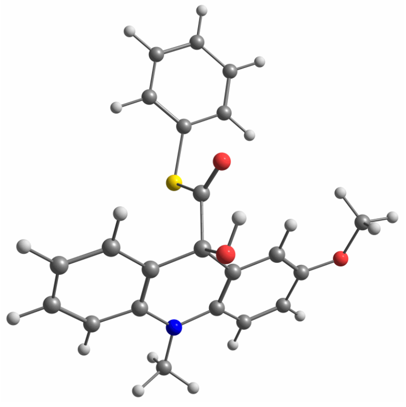

C	0.000002674	0.000003162	-0.000003110
O	-0.000000369	0.000001615	-0.000000078
C	0.000002659	-0.000001512	0.000004019
H	0.000000992	-0.000003074	0.000003667
H	-0.000001422	-0.000004015	0.000004528
H	-0.000003468	-0.000003306	0.000003161
H	-0.000003030	-0.000001702	0.000001471
H	0.000000709	0.000000884	-0.000000944
H	0.000004952	0.000001034	-0.000000389
H	0.000004614	-0.000000576	0.000001340
H	0.000002326	-0.000003024	0.000004280
H	0.000003406	-0.000001941	0.000002890
H	0.000003471	-0.000002061	0.000002474
H	-0.000005179	0.000001312	-0.000001834
H	-0.000002838	0.000002955	-0.000002292
H	-0.000000914	0.000003447	-0.000005130
H	-0.000001556	0.000002640	-0.000002518
H	-0.000001734	0.000001269	-0.000000033
H	0.000002525	0.000003427	-0.000003546
H	0.000001223	0.000002740	-0.000003060
H	0.000001294	0.000002262	-0.000002491

Geom.No
7= -1527.83331883 Ha

Atom	X	Y	Z
C	-0.000003373	-0.000002209	0.000001538
C	0.000002724	0.000001532	-0.000003933
C	-0.000002370	0.000003984	-0.000000497
C	-0.000001610	0.000001617	-0.000006360
C	-0.000004655	-0.000000809	-0.000007473
C	-0.000003392	0.000000770	-0.000003691
C	-0.000035628	-0.000041820	-0.000036249
C	0.000004042	0.000004626	0.000004233
C	0.000000334	-0.000000460	-0.000001451
N	0.000003157	-0.000000276	-0.000003393
C	0.000005248	0.000002229	-0.000001333
C	0.000006336	0.000006750	-0.000002286
C	-0.000008300	-0.000007139	0.000014448
C	0.000004624	0.000001963	-0.000001557
C	-0.000001018	0.000039551	0.000023312
O	-0.000006283	-0.000004802	-0.000007704
O	0.000029612	0.000004490	0.000023926
C	-0.000001933	0.000002828	-0.000003523
S	-0.000004314	-0.000010520	-0.000011889
C	0.000009337	0.000002120	0.000010482
C	0.000002931	-0.000000970	0.000002973
C	-0.000005026	-0.000002674	0.000000902
C	-0.000002719	-0.000004635	0.000004218
C	0.000001973	-0.000002177	0.000003573
C	-0.000004287	0.000001058	0.000007014
H	0.000000613	-0.000001602	-0.000001612

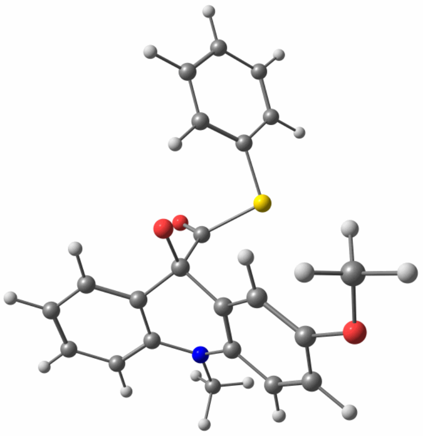

O	0.000031215	-0.000000020	0.000000795
H	-0.000002755	-0.000001234	0.000001506
H	0.000007098	0.000002388	0.000001001
H	0.000005971	0.000004224	-0.000003166
H	-0.000002256	0.000001222	-0.000009441
H	-0.000003875	-0.000001179	-0.000001442
H	-0.000006098	-0.000000843	-0.000003324
H	-0.000004506	0.000000429	-0.000006119
H	-0.000000089	0.000001651	-0.000004351
H	0.000002843	0.000002337	-0.000003306
H	0.000002436	0.000002690	-0.000005471
H	-0.000002360	-0.000001698	0.000003316
H	-0.000004080	-0.000002759	0.000005607
H	-0.000004357	-0.000003183	0.000004613
H	-0.000006949	-0.000002741	0.000001872
C	-0.000021725	0.000004270	-0.000002505
H	0.000009289	0.000000754	0.000005984
H	0.000008018	-0.000000243	0.000004369
H	0.000006157	0.000000510	0.000006395

Geom.No
TS-VIII= -1527.82770160 Ha

Atom	X	Y	Z
C	-0.000001347	-0.000002013	0.000000612
C	-0.000004205	0.000001359	-0.000000969
C	0.000003304	-0.000005311	-0.000004248
C	-0.000002307	0.000000873	0.000000401
C	-0.000001699	-0.000001557	-0.000002187
C	-0.000001605	-0.000002688	-0.000000785
C	0.000033496	-0.000546467	-0.000110390
C	-0.000000243	0.000000474	0.000001923
C	0.000000509	-0.000006438	0.000004920
N	-0.000001334	0.000018323	0.000001985
C	0.000002824	0.000005176	-0.000000429
C	0.000000958	0.000002346	0.000003045
C	0.000005063	-0.000001930	0.000002681
C	-0.000000099	0.000004679	0.000002841
O	-0.000005330	-0.000005562	-0.000004397
C	0.000003379	-0.000000618	-0.000003057
O	0.000001682	0.000008323	0.000005903
C	0.000005069	-0.000001859	0.000004065
C	-0.000994555	0.000792842	0.000890941
O	-0.000000699	0.000003263	-0.000004090
S	0.000968556	-0.000246102	-0.000773286
C	-0.000005488	-0.000010867	-0.000013689
C	0.000001695	0.000001893	0.000003789
C	-0.000004984	0.000001137	-0.000001673
C	-0.000007249	-0.000008598	-0.000007842
C	0.000005827	-0.000000214	0.000000446
C	-0.000007414	-0.000002391	-0.000005290
H	0.000001571	0.000002838	0.000005083

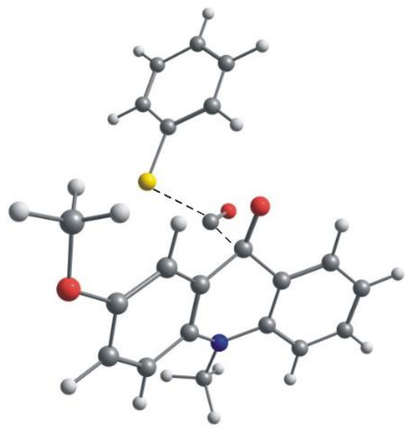

H	0.000001597	0.000000081	0.000002195	
H	0.000004145	0.000004975	0.000002826	
H	0.000001696	0.000004006	0.000001169	
H	0.000000170	0.000001366	-0.000003151	
H	-0.000001287	-0.000003588	0.000000262	
H	-0.000002564	-0.000003764	-0.000000864	
H	-0.000001555	-0.000001530	-0.000001966	
H	0.000000174	0.000003770	-0.000002672	
H	0.000001905	0.000004682	-0.000003148	
H	0.000001594	0.000003409	-0.000001922	
H	-0.000003414	-0.000002329	-0.000002085	
H	-0.000003372	-0.000005804	-0.000000976	
H	-0.000002606	-0.000007504	-0.000000632	
H	-0.000000523	-0.000003459	-0.000000655	
H	0.000003691	0.000001784	0.000005727	
H	0.000001769	0.000001576	0.000004292	
H	0.000003204	0.000001418	0.000005298	

Geom.No 8= -1049.52702930 Ha				
Atom	X	Y	Z	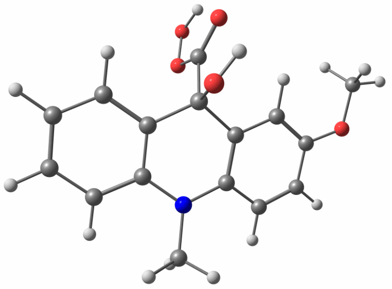
C	0.000016014	0.000000550	-0.000021685	
C	0.000014752	0.000027341	0.000039228	
C	-0.000017750	0.000023787	-0.000000483	
C	0.000006488	-0.000001662	-0.000010959	
C	0.000009147	0.000005503	0.000017789	
C	-0.000022902	-0.000010861	-0.000004702	
C	-0.000018718	0.000012991	-0.000014783	
C	0.000008855	-0.000008746	-0.000018364	
C	0.000013622	0.000020640	0.000006987	
N	0.000013312	-0.000053962	-0.000009351	
C	-0.000005759	0.000006650	0.000000202	
C	0.000015341	-0.000002933	0.000021602	
C	-0.000006901	-0.000006527	-0.000010959	
C	0.000002199	-0.000003728	-0.000002498	
O	0.000005244	0.000019739	-0.000007053	
C	-0.000005440	-0.000023068	-0.000009394	
C	-0.000009919	0.000004948	-0.000003027	
C	0.000008040	-0.000009304	0.000062440	
O	0.000012711	0.000004702	0.000005468	
O	-0.000002666	0.000002745	0.000000072	
O	0.000013102	-0.000014173	-0.000008413	
O	-0.000021200	-0.000006789	-0.000038511	
H	-0.000007710	-0.000005886	0.000004956	
H	0.000003360	0.000000599	0.000000638	
H	0.000000301	-0.000002141	-0.000000740	
H	-0.000003961	0.000000643	-0.000005670	
H	0.000004079	0.000000212	0.000003930	
H	-0.000000966	-0.000001297	-0.000002698	

H	-0.000003019	-0.000001753	-0.000011482
H	0.000004589	-0.000004739	-0.000001479
H	-0.000001391	-0.000002647	0.000006191
H	-0.000004325	-0.000000285	0.000006448
H	-0.000011244	0.000002725	0.000004830
H	-0.000001719	0.000004706	0.000000461
H	-0.000002969	0.000006880	-0.000000039
H	-0.000001123	0.000008391	-0.000004362
H	-0.000001477	0.000006748	0.000005412

Geom.No
8-TS-X= -1049.46413855 Ha

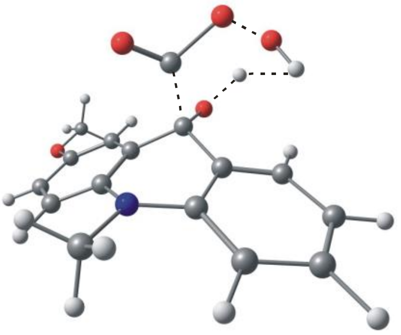

Atom	X	Y	Z
C	0.000003131	0.000004229	0.000010831
C	0.000000146	-0.000007223	-0.000004871
C	-0.000001242	0.000007406	0.000002650
C	0.000007611	0.000000335	-0.000003985
C	-0.000002628	0.000000512	-0.000000842
C	-0.000006649	-0.000002262	0.000006394
N	-0.000011207	0.000020933	0.000012826
C	0.000020076	-0.000017378	-0.000017961
C	0.000000291	0.000000913	0.000010836
C	-0.007484680	-0.030785992	0.004611327
C	0.000000686	-0.000012338	-0.000013925
C	-0.000011749	-0.000018158	-0.000035669
C	0.000005428	0.000013179	0.000028268
C	-0.000004062	-0.000008021	-0.000011883
O	-0.000039432	0.000015555	0.000013259
C	0.000032927	-0.000017244	-0.000010590
O	0.033979430	-0.057539583	-0.021012848
C	0.007488510	0.030761639	-0.004628054
O	-0.002034903	-0.000995767	0.000621520
O	0.002011681	0.000985032	-0.000661893
C	0.000002952	-0.000006976	0.000000705
O	0.000006306	0.000013914	-0.000004069
H	-0.000001337	-0.000005935	-0.000002782
H	-0.000000555	-0.000003518	0.000001600
H	-0.000005717	0.000006278	0.000003685
H	-0.000002239	0.000000927	0.000002462
H	-0.000001514	0.000004504	-0.000001528
H	0.000000844	0.000003882	0.000000039

H	-0.000000363	-0.000000072	0.000000881	
H	-0.000003078	-0.000003437	0.000001556	
H	-0.000000293	-0.000001089	0.000001902	
H	-0.000000431	-0.000001314	0.000000083	
H	-0.033955640	0.057581781	0.021039704	
H	0.000000654	-0.000008058	0.000005380	
H	-0.000009640	0.000018900	0.000035690	
H	0.000007841	-0.000008289	0.000003024	
H	0.000008846	0.000002731	-0.000003720	

Geom.No<br>9= -898.37584698 Ha				
Atom	X	Y	Z	
C	-0.000042747	0.000001605	0.000036150	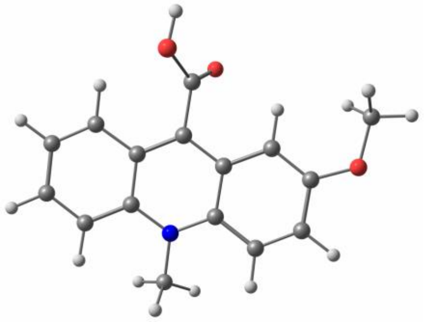
C	0.000004751	-0.000007079	-0.000014416	
C	-0.000030053	-0.000001070	0.000013079	
C	0.000096068	-0.000007244	-0.000025425	
C	-0.000028042	0.000025794	0.000008581	
C	0.000071623	0.000016750	-0.000047774	
C	-0.000034532	0.000017786	0.000035200	
C	0.000000633	-0.000002896	-0.000018682	
C	0.000001299	-0.000009396	0.000005867	
N	0.000003641	0.000005258	0.000002839	
C	-0.000004935	0.000004241	0.000002032	
C	0.000000573	-0.000001424	-0.000005757	
C	0.000015872	0.000000639	-0.000005065	
C	-0.000014769	-0.000001039	0.000021397	
C	-0.000000501	0.000006703	-0.000004983	
C	0.000047213	-0.000035823	-0.000067391	
O	0.000009347	0.000031906	0.000010046	
O	-0.000025444	-0.000015909	0.000037322	
O	-0.000050047	-0.000057329	-0.000037285	
H	-0.000016081	-0.000001780	-0.000010267	
H	-0.000011677	0.000004442	0.000030761	
H	-0.000000541	0.000005977	0.000008359	
H	0.000009633	0.000003726	0.000005880	
H	-0.000009682	-0.000001677	-0.000008418	
H	0.000000729	-0.000002873	-0.000008354	
H	0.000010526	0.000001275	-0.000002599	
H	0.000008261	0.000000081	-0.000003805	
H	-0.000001859	-0.000008576	0.000011390	

H	0.000001100	0.000008479	0.000005180	
H	-0.000004713	-0.000004416	-0.000001856	
C	-0.000000547	0.000031205	0.000054505	
H	0.000011379	0.000000893	-0.000011991	
H	-0.000012535	-0.000001103	-0.000002176	
H	-0.000003941	-0.000007124	-0.000012345	

R_2=CL R_4=H, R_6=H $R_{2'}$=OCH_3				
Geom.No<br>1= -1911.97584791 Ha				
Atom	X	Y	Z	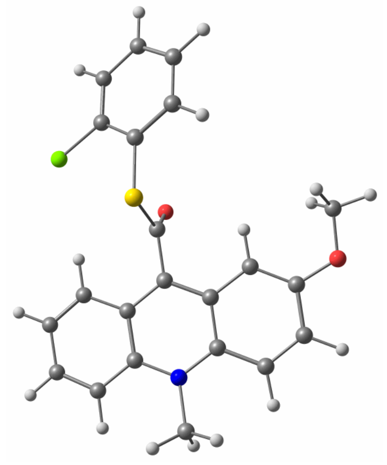
C	0.000005514	-0.000005110	0.000001692	
C	-0.000054238	0.000004760	0.000018249	
C	0.000002965	-0.000002545	-0.000002028	
C	-0.000001473	0.000002396	0.000000234	
C	0.000000512	0.000003719	0.000000528	
C	0.000001749	0.000001352	0.000000178	
S	0.000045787	0.000004677	-0.000037416	
C	0.000012819	-0.000006269	0.000027893	
O	-0.000003696	0.000000591	-0.000007373	
C	0.000000994	0.000011482	-0.000003238	
C	-0.000003029	-0.000004707	0.000000640	
C	-0.000004579	-0.000004011	-0.000000575	
N	0.000006427	0.000001624	-0.000000456	
C	-0.000003196	0.000000977	0.000001336	
C	-0.000002339	-0.000006706	-0.000005590	
C	0.000002177	0.000001567	0.000000889	
C	-0.000001483	-0.000002574	0.000001931	
C	0.000006049	0.000003378	-0.000002868	
C	0.000001745	0.000004500	0.000002840	
C	-0.000000616	0.000003255	0.000002027	
C	0.000002476	-0.000001064	-0.000002967	
C	-0.000002985	-0.000001188	-0.000001583	
C	0.000002588	0.000001701	0.000000278	
O	-0.000005913	-0.000005829	0.000004741	
C	0.000002199	-0.000002582	-0.000000047	
C	-0.000001344	-0.000003908	0.000004110	

H	-0.000001826	0.000005093	0.000001007
H	-0.000003389	0.000001850	-0.000003455
H	-0.000001119	0.000000342	-0.000002796
H	0.000000224	-0.000000006	-0.000001083
H	-0.000000635	-0.000000077	-0.000001452
H	0.000001429	-0.000001730	-0.000001073
H	-0.000000486	-0.000000659	0.000000676
H	0.000001471	0.000002118	-0.000000716
H	-0.000000083	-0.000003208	-0.000005478
H	0.000000023	-0.000001504	-0.000001711
H	-0.000001742	-0.000002585	0.000000649
H	0.000000692	0.000001349	0.000001219
H	0.000000791	0.000001344	0.000000963
H	-0.000000074	0.000000982	-0.000000848
Cl	-0.000003538	-0.000000135	0.000004386
H	-0.000000459	-0.000000941	0.000002601
H	-0.000000007	-0.000000916	0.000001717
H	-0.000000380	-0.000000805	0.000001969

Geom.No
2= -2063.13028762 Ha

Atom	X	Y	Z
C	-0.000000456	0.000001295	0.000005036
C	-0.000007383	-0.000003636	-0.000000468
C	0.000002914	-0.000000323	0.000002446
C	-0.000003646	-0.000002266	0.000002356
C	-0.000001924	0.000001332	-0.000001145
C	0.000000813	0.000000702	0.000000846
C	-0.000004658	0.000006294	-0.000004461
C	-0.000001210	0.000000432	0.000012996
C	-0.000003640	-0.000006042	0.000009018
N	0.000005602	0.000006157	-0.000010673
C	0.000005377	0.000001795	-0.000007385
C	-0.000009398	0.000000241	-0.000001596
C	0.000005120	-0.000001851	0.000012630
C	0.000004774	0.000000201	-0.000014859
C	-0.000013491	0.000029493	0.000004464
O	0.000013001	-0.000003382	0.000001898
O	0.000003644	-0.000009760	0.000012472
O	0.000000959	0.000004519	-0.000015642
C	-0.000004874	0.000000582	-0.000000214
O	-0.000000867	0.000000271	-0.000001615
C	0.000000644	-0.000002137	-0.000000448
S	0.000008608	-0.000022121	-0.000006957
C	0.000005404	0.000002257	0.000007778
C	-0.000006237	-0.000003483	-0.000003223
C	0.000002293	0.000003034	0.000002357
C	0.000001834	0.000000848	-0.000003126
C	-0.000001400	-0.000002644	0.000000637

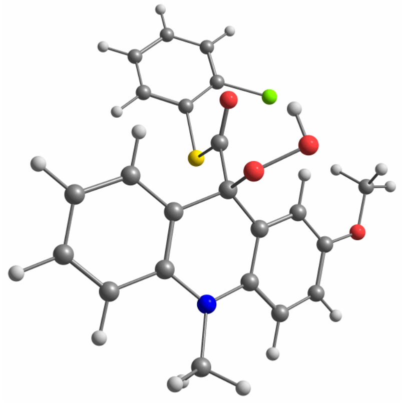

C	0.000000251	0.000000231	-0.000001118
Cl	-0.000001544	-0.000000756	-0.000002577
H	0.000000765	0.000001995	0.000003496
H	0.000000382	0.000000962	0.000001468
H	-0.000001686	0.000001280	-0.000001044
H	-0.000001388	-0.000000744	0.000000321
H	-0.000001325	0.000001082	-0.000001003
H	0.000000040	-0.000001429	-0.000000165
H	0.000002976	0.000000387	-0.000001099
H	0.000001307	-0.000001545	-0.000002545
H	-0.000003628	-0.000001461	0.000003006
H	0.000001139	-0.000000073	0.000001230
H	0.000001122	-0.000000423	-0.000001303
H	-0.000000018	-0.000001127	-0.000001194
H	0.000000439	-0.000000158	-0.000000699
H	0.000000276	-0.000000180	-0.000000984
H	-0.000001994	-0.000002920	0.000001531
H	-0.000000058	0.000000980	-0.000000671
H	0.000000489	0.000001529	-0.000000249
H	0.000000656	0.000000558	0.000000478

Geom.No
6= -1988.00040002 Ha

Atom	X	Y	Z
C	-0.000016123	0.000007690	0.000016174
C	0.000024761	-0.000010500	-0.000027176
C	-0.000003744	-0.000004690	0.000001143
C	-0.000000955	0.000005594	0.000007727
C	0.000001501	0.000010988	-0.000002757
C	0.000010178	-0.000009786	0.000004382
C	-0.000047725	-0.000007671	0.000043867
C	0.000013289	0.000022569	-0.000026727
C	-0.000011578	-0.000008447	-0.000005584
N	0.000014928	0.000006783	0.000012423
C	0.000002609	0.000002341	0.000003318
C	0.000001780	0.000000110	0.000001999
C	-0.000003440	-0.000002601	-0.000000351
C	0.000001374	-0.000004963	-0.000001024
C	-0.000002014	-0.000000477	-0.000007202
O	-0.000000439	-0.000016282	-0.000005224
O	0.000005476	0.000005037	-0.000008024
C	-0.000003575	0.000015282	-0.000005508
S	0.000035446	-0.000001440	0.000020810
C	-0.000038884	0.000009006	-0.000016097
C	0.000001881	-0.000000302	-0.000001895
C	0.000007067	-0.000003474	0.000002365
C	-0.000006932	-0.000009120	0.000003402
C	-0.000000928	-0.000007162	-0.000008089
C	0.000024658	0.000001151	-0.000011596

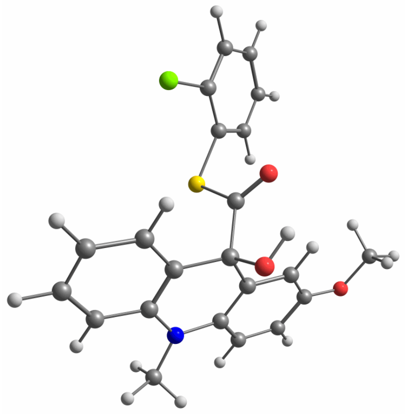

Cl	-0.000006236	-0.000011231	0.000008259
H	0.000002582	0.000000316	-0.000002106
H	-0.000000745	-0.000001030	-0.000000685
H	-0.000001398	-0.000001029	-0.000000725
H	-0.000006053	-0.000003115	0.000001300
H	-0.000002185	0.000001737	-0.000000127
O	-0.000007768	0.000013477	-0.000005984
H	0.000002373	0.000005553	0.000000937
H	0.000003582	0.000002962	0.000001274
H	0.000000459	0.000002750	0.000002497
H	0.000004653	-0.000000864	0.000001333
H	0.000002799	0.000003497	0.000000296
H	0.000000578	-0.000009256	-0.000000317
H	0.000001596	-0.000007091	-0.000002719
H	-0.000000398	-0.000004243	-0.000001316
H	0.000002303	-0.000002442	0.000001404
H	-0.000005628	0.000003456	0.000002899
C	0.000008632	-0.000007577	0.000004379
H	-0.000001300	0.000006460	-0.000000024
H	-0.000002415	0.000003203	-0.000001931
H	-0.000004045	0.000004831	0.000001001

Geom.No
7= -1987.42880981 Ha

Atom	X	Y	Z
C	0.000003239	-0.000001666	-0.000004981
C	-0.000000211	-0.000007384	-0.000002646
C	0.000000174	-0.000006201	-0.000001753
C	-0.000002169	0.000000384	0.000001773
C	0.000002770	-0.000002011	0.000003026
C	-0.000003323	-0.000003230	0.000000932
C	0.000028800	0.000085594	0.000055126
C	0.000016866	-0.000004626	0.000001657
C	-0.000009558	0.000010096	-0.000014414
N	0.000000886	0.000003004	-0.000001190
C	-0.000005604	-0.000013137	0.000013949
C	0.000019977	0.000000602	-0.000000986
C	0.000005040	0.000001716	-0.000023423
C	-0.000015245	-0.000006181	0.000018146
C	0.000034210	-0.000053281	-0.000039659
O	-0.000010235	-0.000002331	-0.000000173
O	-0.000039047	-0.000008028	-0.000017705
S	0.000002642	0.000004105	-0.000002800
C	-0.000068052	0.000030534	-0.000018469
C	0.000015086	-0.000014666	0.000015993
C	0.000012915	-0.000017173	-0.000001015
C	-0.000000328	-0.000003637	-0.000004495
C	0.000012310	-0.000012835	0.000003826
C	-0.000033178	0.000027863	-0.000006264
C	0.000038940	-0.000002027	0.000074133
Cl	0.000017796	-0.000030609	-0.000045663

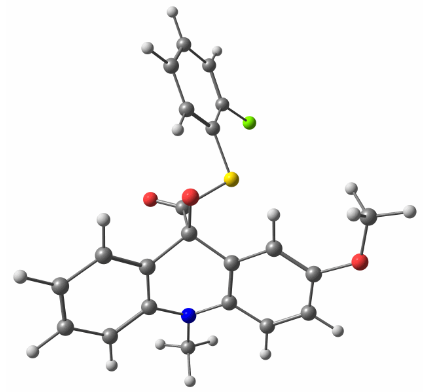

O	-0.000032552	0.000023865	0.000012996	
H	0.000003646	0.000004586	-0.000000815	
H	-0.000004704	0.000001760	0.000001200	
H	-0.000001380	0.000004046	0.000000005	
H	0.000001515	0.000001584	0.000002712	
H	-0.000001422	-0.000001647	0.000002723	
H	-0.000000039	-0.000002554	-0.000000297	
H	-0.000000332	-0.000001127	-0.000000679	
H	0.000001157	0.000002234	-0.000002793	
H	0.000001012	0.000003622	-0.000002486	
H	0.000000121	0.000001700	0.000000645	
H	-0.000017582	0.000006368	-0.000028034	
H	-0.000002705	-0.000000397	-0.000000780	
H	-0.000001774	-0.000001102	-0.000001112	
H	0.000003342	-0.000003194	0.000000256	
C	0.000033559	-0.000017970	0.000008351	
H	-0.000003202	0.000000193	0.000001289	
H	-0.000003037	0.000000979	0.000002713	
H	-0.000000325	0.000002178	0.000001180	

Geom.No<br>TS-VIII= -1987.42374554 Ha				
Atom	X	Y	Z	
C	0.000009101	0.000003073	0.000006154	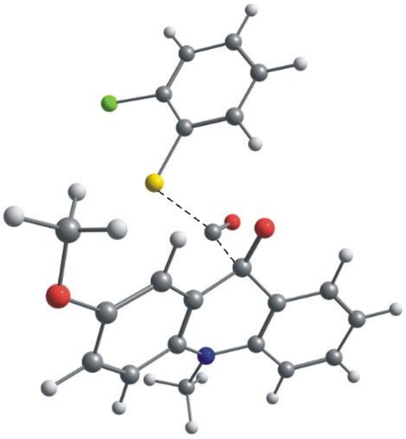
C	-0.000026951	0.000000658	0.000003787	
C	0.000009151	-0.000002991	-0.000011754	
C	-0.000002130	0.000001084	0.000006881	
C	0.000001216	0.000002445	-0.000002382	
C	0.000002822	0.000000601	0.000001836	
S	0.002020643	0.002607214	0.001897847	
C	-0.005363842	-0.000612347	-0.004210210	
O	0.000000027	-0.000002842	-0.000006779	
Cl	-0.000001374	-0.000000235	0.000007928	
C	0.003363863	-0.001972729	0.002299522	
C	-0.000001387	-0.000005611	0.000006741	
C	-0.000008749	0.000011844	-0.000001152	
N	0.000018589	-0.000006910	0.000007397	
C	-0.000003781	-0.000003414	-0.000004536	
C	-0.000003838	-0.000007001	-0.000003307	
C	0.000004546	-0.000006327	-0.000003060	
C	-0.000002059	-0.000002953	0.000000828	
C	0.000001780	0.000004223	0.000000195	
C	-0.000000169	-0.000004610	0.000000989	
C	0.000002736	0.000001858	-0.000001133	
C	0.000002342	0.000000832	-0.000004905	
C	-0.000000232	-0.000001818	-0.000004708	
C	0.000003357	0.000007439	0.000005675	
O	-0.000002727	-0.000003088	-0.000001918	
C	0.000000857	-0.000001282	0.000002951	

O	-0.000011087	-0.000009205	0.000013351
C	-0.000013583	0.000001824	-0.000001313
H	-0.000001305	0.000002451	0.000000966
H	-0.000001691	-0.000002286	-0.000000189
H	-0.000002095	-0.000003066	-0.000000050
H	-0.000000368	-0.000000218	-0.000006495
H	0.000001273	-0.000000036	-0.000001908
H	0.000001936	0.000001854	-0.000001569
H	0.000001135	0.000000758	-0.000002103
H	0.000001557	0.000001548	-0.000000282
H	0.000000976	-0.000000578	-0.000000946
H	0.000001245	-0.000000629	-0.000003066
H	-0.000001028	0.000000400	0.000002932
H	0.000000190	0.000001470	0.000002396
H	-0.000001029	0.000001702	0.000001530
H	0.000003243	-0.000001931	-0.000002155
H	-0.000001051	-0.000000781	0.000002429
H	-0.000000941	-0.000000766	0.000001643
H	-0.000001167	0.000000375	0.000001944

$R_2=CH_3$ $R_4=H$, $R_6=H$ $R_{2'}=OCH_3$

Geom.No
1= -1491.70525299 Ha

Atom	X	Y	Z
C	-0.000004019	-0.000000730	0.000007995
C	-0.000001571	-0.000001682	-0.000001493
C	-0.000004184	-0.000000885	-0.000002777
C	-0.000003952	-0.000003414	-0.000008118
C	-0.000009826	-0.000002492	0.000009453
C	0.000008350	-0.000001587	-0.000011505
C	0.000004349	-0.000004856	-0.000001173
C	-0.000004614	0.000000891	-0.000002127
C	0.000007445	0.000000958	-0.000005514
N	0.000002273	-0.000002416	-0.000004572
C	-0.000001693	-0.000002529	0.000007526
C	0.000007616	0.000001466	-0.000001412
C	0.000001361	0.000002270	-0.000001836
C	-0.000000434	0.000001838	0.000004891
C	-0.000014375	0.000028235	0.000000087
O	0.000000856	-0.000007779	0.000002987
C	0.000004011	-0.000003466	-0.000007005
S	0.000011581	-0.000014108	0.000000462
C	-0.000005662	-0.000002744	0.000002269
C	-0.000000253	0.000004208	0.000003434
C	0.000002202	0.000005291	0.000004525
C	-0.000001744	0.000005322	0.000001518
C	-0.000003669	0.000000139	0.000001304

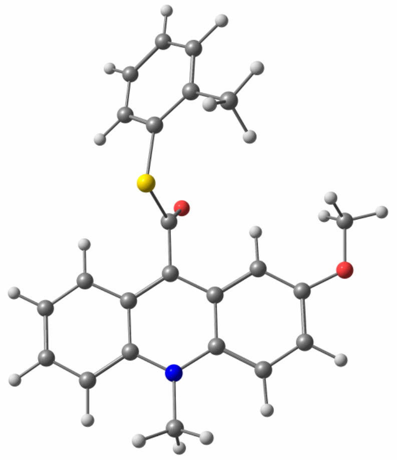

C	0.000005740	0.000004296	0.000004118
C	0.000002001	0.000000891	0.000000308
H	-0.000000809	-0.000002086	-0.000000841
H	0.000002675	0.000001384	0.000000092
H	0.000003215	0.000002821	0.000002067
H	0.000001908	0.000002099	0.000003007
H	0.000001427	-0.000000311	0.000001849
O	-0.000014994	-0.000003409	-0.000010639
H	0.000000338	-0.000004813	-0.000006753
H	0.000001316	-0.000002138	-0.000001085
H	0.000005603	0.000000826	-0.000006427
H	0.000000283	-0.000002765	-0.000002071
H	0.000002996	-0.000005486	-0.000006080
H	-0.000000414	0.000002779	0.000002484
H	-0.000001727	0.000003502	0.000004679
H	-0.000002157	0.000004218	0.000004912
H	-0.000000919	0.000002421	0.000002777
H	-0.000000103	0.000001242	0.000001422
H	0.000001749	0.000002326	0.000000309
H	-0.000000698	-0.000000004	-0.000000924
C	0.000017460	-0.000004930	0.000018646
H	-0.000009877	-0.000002333	-0.000003437
H	-0.000005492	0.000001467	-0.000003877
H	-0.000003571	-0.000003925	-0.000003456

Geom.No
2= -1642.85987970 Ha

Atom	X	Y	Z
C	0.000005442	-0.000006259	-0.000010519
C	0.000002669	0.000001301	0.000012197
C	-0.000011606	0.000003578	0.000001760
C	0.000006540	-0.000000976	-0.000002815
C	0.000004180	-0.000007002	0.000001866
C	-0.000019629	-0.000000853	0.000000434
C	0.000001623	-0.000001019	-0.000010582
C	-0.000009027	0.000004802	0.000002871
C	0.000002330	0.000002698	0.000002462
N	0.000003477	-0.000013009	0.000002487
C	-0.000000735	0.000000250	-0.000001806
C	-0.000002604	0.000001471	-0.000000191
C	0.000000713	0.000003700	0.000001912
C	0.000002883	0.000002147	-0.000001631
C	-0.000006125	-0.000007104	0.000005385
O	0.000002229	0.000005261	-0.000000314
O	-0.000005447	-0.000001306	0.000000937
O	-0.000000748	0.000000271	0.000002772
C	-0.000003247	0.000001861	-0.000000047
O	0.000012720	-0.000007972	0.000005433
C	-0.000006909	0.000003407	-0.000000561
S	0.000002558	0.000003262	-0.000003385
C	-0.000000871	0.000002605	0.000000459

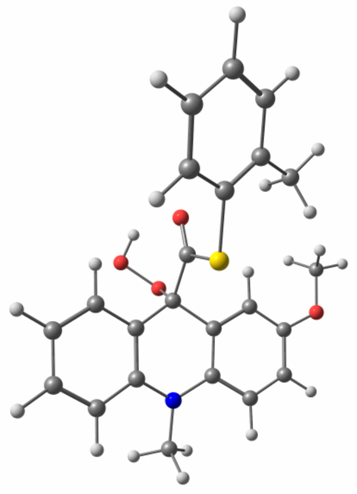

C	0.000004705	-0.000000573	0.000001054	
C	0.000001751	0.000001534	-0.000002235	
C	0.000002668	0.000002448	-0.000003186	
C	0.000002863	0.000002818	-0.000001885	
C	0.000002670	0.000002926	-0.000002952	
C	0.000001576	0.000001135	-0.000000980	
H	-0.000002254	0.000000652	0.000001254	
H	-0.000001190	0.000002811	0.000000009	
H	-0.000000737	0.000003660	-0.000000118	
H	-0.000001189	0.000002961	-0.000000234	
H	-0.000001475	0.000000891	-0.000000614	
H	-0.000001859	-0.000002894	0.000001071	
H	-0.000001219	-0.000004264	0.000005188	
H	-0.000002039	-0.000001194	-0.000000150	
H	-0.000001298	-0.000003465	-0.000000594	
H	-0.000000281	-0.000002164	0.000000637	
H	0.000002440	0.000002478	-0.000001812	
H	0.000002796	0.000004157	-0.000002837	
H	0.000003555	0.000003897	-0.000002386	
H	0.000002735	0.000001917	-0.000001895	
H	0.000002699	0.000004435	0.000000302	
H	0.000001759	-0.000000289	-0.000000944	
H	0.000001964	-0.000000927	0.000000216	
H	-0.000000574	-0.000000658	-0.000000797	
H	0.000000748	-0.000003396	0.000002997	
H	-0.000002743	-0.000004415	0.000001911	
H	0.000001517	-0.000005599	-0.000000143	

Geom.No 6= -1567.73156204 Ha				
Atom	X	Y	Z	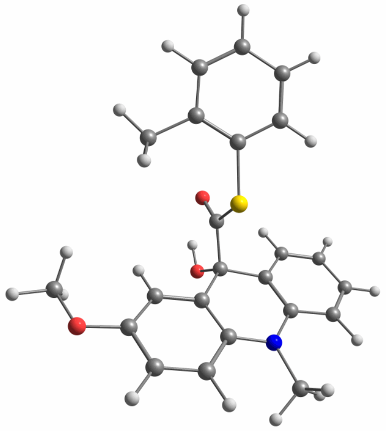
C	0.000002644	-0.000001787	-0.000001788	
C	0.000001155	-0.000000229	-0.000010593	
C	-0.000007004	0.000003208	0.000003959	
C	0.000001359	-0.000000030	-0.000004477	
C	0.000001799	-0.000000082	0.000000605	
C	-0.000003317	-0.000001892	-0.000001101	
S	0.000001687	0.000003237	0.000000651	
C	-0.000004673	-0.000018882	-0.000001672	
O	0.000001271	0.000005845	-0.000001009	
C	0.000002208	-0.000000989	-0.000001577	
C	0.000009243	0.000023353	0.000003810	
C	-0.000000079	-0.000005364	-0.000002922	
C	-0.000005147	0.000007648	0.000002980	
N	-0.000000162	-0.000006189	0.000001941	
C	-0.000000108	0.000001828	-0.000008144	
C	-0.000003419	-0.000000640	0.000001705	
C	0.000005032	0.000000697	0.000006507	
C	0.000001414	-0.000001887	-0.000002331	
C	-0.000001659	-0.000002137	-0.000009481	
C	-0.000000794	0.000002177	0.000006687	

C	0.000000055	-0.000002324	0.000002888
C	-0.000002535	0.000000264	0.000000285
C	0.000001593	-0.000003142	-0.000003739
C	0.000003503	-0.000001781	0.000004333
O	-0.000006666	-0.000009867	0.000008243
C	-0.000002124	-0.000004002	0.000002302
H	0.000000133	-0.000002032	0.000001558
H	-0.000000826	-0.000002752	0.000000392
H	-0.000000663	-0.000002030	0.000000505
H	-0.000001261	0.000000504	-0.000001415
H	0.000001023	0.000001020	0.000000175
O	0.000005925	-0.000000348	0.000005951
H	0.000001932	0.000003340	0.000004008
H	-0.000000905	0.000001130	-0.000001149
H	0.000000535	-0.000000153	0.000001322
H	0.000000807	-0.000002489	-0.000000193
H	0.000001013	-0.000000764	0.000003430
H	0.000001779	0.000001675	-0.000004084
H	0.000000221	0.000000201	-0.000001456
H	-0.000000533	-0.000000995	-0.000001670
H	-0.000000090	-0.000002414	-0.000001486
H	-0.000000812	-0.000001012	-0.000000489
H	-0.000000149	0.000000364	-0.000001969
H	0.000000562	0.000002369	-0.000001906
H	-0.000000450	0.000003078	-0.000000288
C	-0.000006880	0.000007402	-0.000000555
H	-0.000000475	0.000002503	0.000001720
H	0.000001433	0.000001526	0.000000026
H	0.000002406	0.000002845	-0.000000489

Geom.No
7= -1567.15318939 Ha

Atom	X	Y	Z
C	0.000001184	-0.000001736	0.000001354
C	0.000004875	0.000003728	0.000000573
C	-0.000002574	0.000004022	0.000007091
C	0.000001137	0.000001391	-0.000001455
C	0.000000080	-0.000000967	0.000000797
C	-0.000000540	-0.000000574	-0.000001014
C	-0.000039844	-0.000041839	-0.000029621
C	0.000005807	0.000003447	0.000007230
C	-0.000001634	-0.000000983	0.000002043
N	0.000003587	-0.000004345	-0.000003412
C	0.000002206	0.000002144	0.000001971
C	0.000000953	0.000003716	-0.000001605
C	-0.000021613	-0.000021300	0.000022000
C	0.000002481	0.000005652	-0.000003843
C	0.000012002	0.000038082	0.000003307
O	-0.000006279	-0.000005720	-0.000006170
O	0.000032344	0.000007356	0.000013788
C	-0.000003690	0.000003496	0.000001622

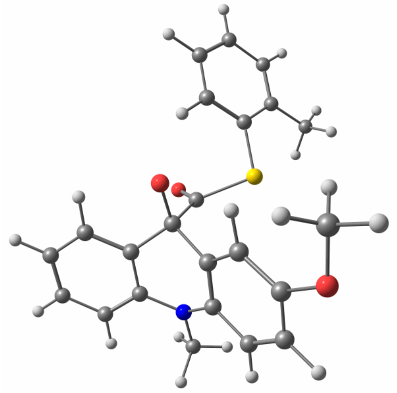

S	-0.000018399	-0.000006765	-0.000000436	
C	0.000008536	-0.000001844	0.000005259	
C	0.000006674	-0.000003984	0.000003400	
C	-0.000003337	-0.000002397	-0.000005029	
C	-0.000000711	-0.000004494	-0.000003228	
C	0.000002369	0.000000722	-0.000002806	
C	-0.000005233	0.000000672	-0.000001997	
C	-0.000003094	-0.000000896	0.000000168	
O	0.000038417	0.000015972	-0.000003784	
H	0.000000292	-0.000001122	0.000000861	
H	0.000003090	0.000002324	0.000003103	
H	0.000001861	0.000003647	0.000001560	
H	-0.000000571	0.000000552	0.000000174	
H	-0.000000411	-0.000001074	-0.000000140	
H	0.000000551	-0.000001152	0.000000162	
H	0.000000271	0.000000156	0.000000580	
H	-0.000000375	0.000001770	0.000001045	
H	-0.000000340	0.000002210	0.000002149	
H	0.000000516	0.000002600	0.000001860	
H	-0.000002796	-0.000001610	-0.000002579	
H	-0.000001323	-0.000002146	-0.000002314	
H	-0.000000234	-0.000003130	-0.000001327	
H	-0.000003709	-0.000000000	-0.000001175	
C	-0.000022769	0.000001835	-0.000007581	
H	0.000004621	0.000001038	0.000002005	
H	0.000004486	-0.000000799	0.000000629	
H	0.000004478	0.000000621	0.000003327	
H	-0.000003346	-0.000001092	-0.000002929	
H	0.000001558	-0.000000566	-0.000003697	
H	-0.000001555	0.000003383	-0.000001918	

Geom.No TS-VIII= -1567.14780310 Ha				
Atom	X	Y	Z	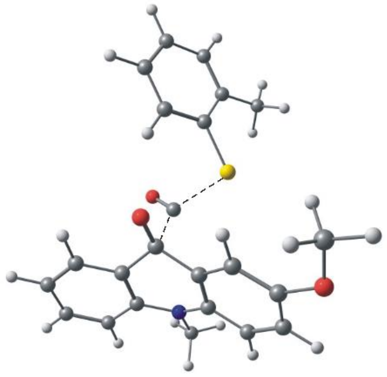
C	0.000005590	0.000003953	-0.000004136	
C	0.000006256	-0.000008272	0.000008300	
C	-0.000000746	0.000000537	0.000001478	
C	-0.000000662	-0.000000002	-0.000000600	
C	0.000002551	-0.000001247	0.000005393	
C	-0.000005799	0.000000610	0.000000869	
S	-0.000479903	-0.000702686	-0.000499474	
C	0.001174866	0.000350014	0.000944493	
O	-0.000007999	0.000011241	-0.000005319	
C	0.000003336	-0.000001212	-0.000001887	
C	-0.000702342	0.000333639	-0.000429266	
C	0.000007802	-0.000003004	-0.000019986	
C	-0.000012629	0.000005587	0.000004052	
N	0.000020395	-0.000003053	0.000006976	
C	-0.000014619	-0.000013081	-0.000009316	
C	0.000009212	0.000021132	0.000003300	
C	-0.000001551	-0.000001471	0.000001014	

C	0.000003216	-0.000002240	0.000001550
C	0.000007647	-0.000007234	0.000007346
C	-0.000008276	0.000002898	0.000000470
C	0.000002357	0.000005164	0.000002304
C	-0.000000264	-0.000000920	-0.000003143
C	0.000001635	0.000006871	0.000001370
C	-0.000003490	-0.000007249	-0.000006185
O	-0.000012265	0.000005375	-0.000009704
C	0.000008164	-0.000001014	0.000007826
O	0.000006723	0.000005362	-0.000003091
C	-0.000006531	-0.000001015	-0.000003245
H	0.000002019	0.000000102	0.000003363
H	0.000000289	0.000000760	-0.000000004
H	0.000000502	0.000001842	-0.000001822
H	-0.000000755	0.000001102	-0.000002535
H	0.000001108	0.000001569	-0.000000522
H	0.000000441	0.000001297	-0.000002472
H	0.000000267	0.000002435	-0.000004327
H	-0.000000529	-0.000000429	-0.000002236
H	-0.000000284	-0.000002784	-0.000002300
H	-0.000000886	0.000000234	-0.000001842
H	0.000000456	-0.000001423	0.000001396
H	0.000000160	0.000000196	0.000000705
H	0.000002425	0.000000988	0.000001302
H	-0.000003341	-0.000001080	0.000000357
H	0.000001096	0.000000253	0.000002315
H	-0.000000548	-0.000001748	0.000000517
H	-0.000000411	0.000003178	0.000002311
H	-0.000001464	-0.000001655	0.000000760
H	-0.000001942	-0.000001539	0.000002390
H	-0.000001279	-0.000001981	0.000001254

R_2=C(CH$_3$)$_3$ R_4=H, R_6=H $R_{2'}$=OCH$_3$

Geom.No
1= -1609.64285040 Ha

Atom	X	Y	Z
C	0.000000305	-0.000000495	0.000002897
C	0.000002081	-0.000000903	0.000001350
C	0.000000681	-0.000000442	0.000001134
C	0.000001388	0.000001466	0.000001993
C	0.000000955	0.000000716	0.000002014
C	0.000003072	-0.000001359	0.000002213
C	-0.000000629	0.000003502	-0.000008294
C	-0.000003753	-0.000000438	-0.000001361
C	-0.000006223	0.000004466	0.000002903
N	-0.000000271	0.000003159	0.000003606
C	-0.000001836	0.000002765	-0.000001341
C	0.000000057	0.000001037	0.000002144
C	-0.000008927	0.000004445	0.000006968
C	-0.000000679	0.000001098	-0.000005478
C	0.000007341	-0.000011790	0.000014553

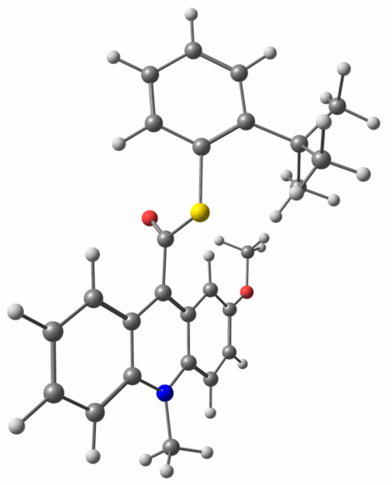

O	-0.000003621	0.000005819	-0.000003970	
C	-0.000002399	0.000001654	0.000004825	
O	0.000003027	0.000001409	-0.000009583	
C	-0.000018519	-0.000000814	-0.000000117	
S	0.000003214	-0.000003209	-0.000001193	
C	-0.000001824	0.000005107	-0.000007502	
C	0.000001010	0.000003588	0.000003972	
C	-0.000002889	-0.000002300	-0.000008902	
C	0.000003642	-0.000004245	-0.000001153	
C	0.000002524	-0.000005429	0.000000398	
C	0.000003297	-0.000005640	-0.000005460	
C	0.000000009	0.000000746	0.000002622	
C	0.000004777	-0.000001012	-0.000000661	
C	0.000000271	-0.000005429	-0.000004503	
C	0.000005418	0.000001861	0.000004764	
H	-0.000000902	0.000001944	0.000002523	
H	0.000001809	-0.000000755	0.000003719	
H	0.000002671	-0.000001024	0.000002163	
H	0.000001210	-0.000001341	0.000000162	
H	0.000001110	-0.000000186	-0.000000983	
H	-0.000005725	0.000005342	-0.000000584	
H	-0.000000668	0.000002521	0.000000326	
H	-0.000001585	0.000002430	0.000006090	
H	-0.000001515	0.000003051	0.000003118	
H	-0.000003426	0.000004198	0.000005739	
H	0.000002139	-0.000003659	-0.000004629	
H	0.000003002	-0.000004544	-0.000004986	
H	0.000003019	-0.000004297	-0.000003994	
H	0.000002009	-0.000002689	-0.000000946	
H	-0.000000790	-0.000002945	-0.000003212	
H	0.000002609	-0.000004577	0.000001129	
H	0.000000488	-0.000000262	-0.000004621	
H	-0.000001041	0.000000850	-0.000001339	
H	0.000000050	0.000001944	0.000002002	
H	-0.000002734	0.000001215	-0.000000297	
H	0.000001955	-0.000001344	0.000001171	
H	0.000001425	-0.000000358	0.000000899	
H	0.000001860	-0.000002325	0.000002941	
H	-0.000003174	0.000004465	-0.000005373	
H	0.000001849	-0.000001233	0.000001695	
H	0.000002858	0.000004246	-0.000001550	

Geom.No<br>2= -1760.79577458 Ha				
Atom	X	Y	Z	

C	-0.000003443	0.000001609	0.000000066	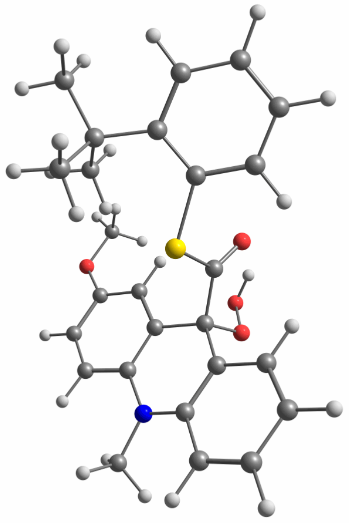
C	-0.000005958	0.000002535	0.000004837	
C	-0.000001707	0.000002091	-0.000002248	
C	-0.000003540	0.000004176	-0.000000656	
C	-0.000002038	0.000003446	-0.000000535	
C	-0.000002752	0.000002404	0.000000315	
C	0.000009489	-0.000012963	-0.000001961	
C	-0.000006112	-0.000000595	-0.000001527	
C	-0.000000918	-0.000000029	0.000004122	
N	-0.000002436	0.000004628	-0.000002424	
C	-0.000000593	0.000001699	-0.000003732	
C	-0.000005136	-0.000000671	0.000000207	
C	0.000001210	-0.000001127	0.000004171	
C	0.000000895	-0.000001471	-0.000003927	
C	-0.000011181	0.000007354	-0.000004757	
O	0.000004719	-0.000004371	0.000004464	
O	-0.000008031	0.000007191	-0.000003164	
O	-0.000004854	-0.000002186	0.000001314	
C	-0.000003026	0.000003745	-0.000001135	
O	-0.000003525	0.000002805	-0.000002342	
C	-0.000000966	0.000000716	-0.000000123	
S	0.000003126	-0.000007758	-0.000000051	
C	-0.000000856	0.000005129	0.000004948	
C	0.000005905	-0.000004337	0.000001197	
C	0.000002554	-0.000007626	-0.000000795	
C	0.000001325	-0.000003414	0.000002553	
C	0.000007580	-0.000002209	0.000001681	
C	0.000002572	-0.000005645	-0.000002630	
C	0.000004384	0.000000047	0.000000054	
C	0.000002796	0.000000700	0.000001025	
C	0.000004546	0.000000995	0.000001279	
C	0.000004807	-0.000001018	0.000000571	
H	0.000000803	-0.000004517	0.000000944	
H	-0.000002719	0.000004766	-0.000001215	
H	-0.000002898	0.000004896	-0.000001429	
H	-0.000002047	-0.000000547	-0.000000213	
H	-0.000001606	-0.000002901	0.000000672	
H	-0.000001469	-0.000002118	-0.000000415	
H	-0.000000854	0.000000021	-0.000000116	
H	-0.000001297	0.000002415	-0.000000824	
H	-0.000004086	0.000003947	-0.000000943	
H	-0.000004192	0.000003880	-0.000000681	
H	-0.000002046	0.000004790	-0.000000630	
H	0.000002828	-0.000006171	0.000001919	
H	0.000004661	-0.000005454	0.000001350	
H	0.000004896	-0.000003036	0.000000904	
H	-0.000003438	-0.000002253	-0.000000577	
H	0.000004649	0.000000907	0.000000109	
H	0.000006089	-0.000000736	0.000000543	
H	0.000004308	-0.000001014	0.000000598	
H	0.000001966	0.000000983	0.000000127	

H	0.000001344	-0.000000620	0.000000131	
H	0.000001299	0.000001026	-0.000000167	
H	0.000004508	0.000002215	0.000000265	
H	0.000003298	0.000000914	0.000000260	
H	0.000005167	0.000000584	0.000000409	
H	-0.000002863	0.000001647	-0.000000758	
H	-0.000001610	0.000000216	-0.000000306	
H	-0.000003527	0.000000309	-0.000000756	

Geom.No<br>6= -1685.66762639 Ha				
Atom	X	Y	Z	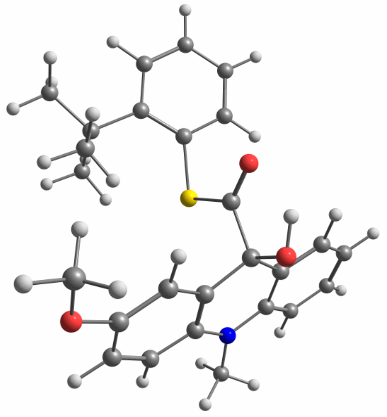
C	-0.000001480	0.000000001	-0.000001491	
C	0.000003101	0.000001296	0.000000504	
C	-0.000001050	0.000004098	-0.000004969	
C	0.000000127	0.000001776	-0.000002482	
C	-0.000000724	0.000001076	-0.000002008	
C	-0.000002362	0.000000266	-0.000000609	
C	-0.000002572	-0.000001024	0.000000719	
C	0.000002068	0.000000812	0.000000989	
C	0.000004107	0.000002770	-0.000003142	
N	-0.000000322	-0.000003396	0.000003771	
C	0.000002542	0.000001081	-0.000000749	
C	0.000005154	0.000000996	-0.000000057	
C	0.000003479	0.000002396	-0.000002601	
C	0.000004562	0.000000046	-0.000000378	
C	0.000001806	-0.000002927	0.000004155	
O	-0.000002781	-0.000001339	-0.000000719	
O	0.000002375	0.000001649	0.000001252	
C	-0.000000050	0.000003720	-0.000002442	
O	0.000001270	-0.000000728	0.000002382	
C	0.000003072	-0.000001007	0.000003192	
S	0.000000340	0.000000478	-0.000001899	
C	-0.000005163	-0.000000122	0.000005098	
C	-0.000000878	-0.000003001	-0.000001508	
C	-0.000002618	-0.000001315	-0.000000252	
C	-0.000004332	-0.000001832	0.000001938	
C	-0.000003276	-0.000002513	-0.000000587	
C	-0.000001374	-0.000001376	-0.000000786	
C	0.000000238	0.000000912	0.000002112	
C	-0.000001176	-0.000001609	0.000000418	
C	-0.000000919	0.000000510	-0.000000574	
C	-0.000000349	-0.000000844	0.000000522	
H	-0.000000682	0.000001879	-0.000003301	
H	-0.000002007	0.000001433	-0.000002901	
H	-0.000002247	0.000000106	-0.000001395	
H	-0.000001446	-0.000000921	0.000000260	
H	0.000001105	-0.000000379	0.000002089	
H	0.000003819	0.000002236	-0.000000479	
H	0.000003520	0.000003082	-0.000001960	
H	0.000001224	0.000001519	-0.000002769	

H	0.000001698	0.000003085	-0.000003640
H	0.000001521	0.000003834	-0.000001799
H	-0.000002973	-0.000001752	0.000001757
H	-0.000004293	-0.000002594	0.000001204
H	-0.000004427	-0.000001967	0.000000587
H	-0.000002532	-0.000001513	-0.000000164
H	-0.000001718	-0.000001212	-0.000000641
H	-0.000000396	-0.000001068	0.000000338
H	-0.000002032	-0.000000993	0.000000377
H	-0.000001181	-0.000001751	0.000001783
H	-0.000000296	0.000000630	-0.000000101
H	-0.000000930	0.000000255	-0.000000727
H	-0.000001007	0.000000202	-0.000000468
H	0.000001530	-0.000001412	0.000001723
H	0.000000260	-0.000001325	0.000001787
H	0.000001726	-0.000000937	0.000001163
H	0.000003419	-0.000000247	0.000002639
H	0.000002134	-0.000000587	0.000002430
H	0.000003397	-0.000000452	0.000002408

Geom.No 7= -1685.09123221 Ha

Atom	X	Y	Z
C	0.000001022	-0.000002302	-0.000006433
C	-0.000004609	-0.000001878	0.000000536
C	0.000003280	-0.000002115	-0.000005835
C	-0.000003636	-0.000001855	-0.000000344
C	-0.000001214	-0.000003175	-0.000000572
C	-0.000003602	-0.000004750	-0.000000338
C	-0.000012785	0.000014104	0.000002052
C	0.000004583	0.000006260	0.000003779
C	-0.000006350	-0.000004300	0.000002830
N	0.000002022	0.000002278	-0.000001589
C	0.000001761	-0.000002964	0.000005748
C	0.000010449	0.000004180	0.000000740
C	-0.000008854	-0.000002088	0.000000982
C	-0.000007180	-0.000004482	0.000007037
C	0.000031080	0.000016162	-0.000015242
O	-0.000012586	-0.000020132	0.000009686
O	-0.000000376	-0.000012205	-0.000001189
C	0.000003898	0.000000398	-0.000005449
S	-0.000018192	-0.000008262	0.000004457

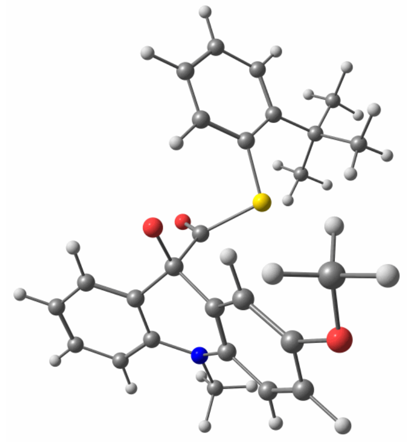

C	-0.000002524	0.000017752	-0.000003190
C	0.000015120	-0.000016007	0.000014874
C	-0.000014734	0.000014462	-0.000004676
C	0.000007411	-0.000013957	-0.000001721
C	-0.000018882	0.000017317	-0.000002835
C	0.000022066	-0.000016437	0.000012825
C	-0.000004378	0.000000423	-0.000014387
O	-0.000006592	0.000004823	0.000001847
H	-0.000000652	0.000002488	0.000000327
H	-0.000001205	0.000002919	0.000001357
H	0.000001069	0.000003448	-0.000003041
H	-0.000000471	-0.000002386	-0.000001673
H	-0.000003759	-0.000004946	0.000002591
H	-0.000004414	-0.000004360	-0.000001335
H	-0.000002844	-0.000004177	-0.000002854
H	-0.000000077	0.000000585	-0.000003914
H	0.000001446	0.000002926	-0.000002631
H	-0.000001163	0.000000359	-0.000001309
H	0.000002386	-0.000004703	-0.000006742
H	-0.000001987	-0.000000595	-0.000001863
H	-0.000002736	-0.000004479	0.000001093
H	-0.000008085	0.000001376	-0.000003016
C	0.000016547	0.000000530	0.000012276
H	-0.000000136	0.000000882	0.000006465
H	-0.000001722	0.000003276	0.000004169
H	-0.000001833	-0.000001213	0.000001672
C	0.000000918	-0.000001996	-0.000003151
C	0.000000404	-0.000005705	0.000010750
C	0.000025504	0.000009236	0.000006427
H	-0.000000028	0.000002527	-0.000000455
H	0.000002947	0.000002563	-0.000000949
H	0.000002744	0.000004347	-0.000001411
H	0.000003049	0.000003644	-0.000002672
H	0.000001673	0.000001450	0.000002211
H	0.000000405	0.000000917	-0.000000898
H	-0.000005238	0.000008450	-0.000006436
H	-0.000001836	0.000002391	-0.000001082
H	0.000002899	-0.000001004	-0.000007497

Geom.No
TS-VIII= -1685.08630081 Ha

Atom	X	Y	Z

O	-0.000001750	0.000000698	0.000000601	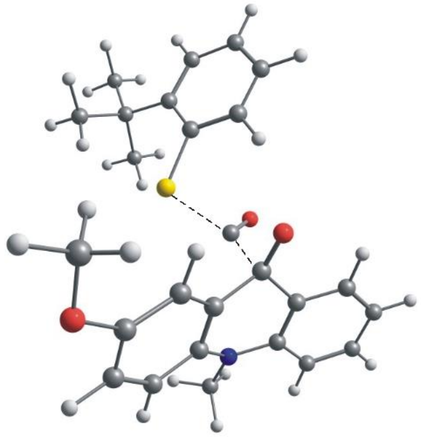
C	0.000001211	-0.000001912	0.000003256	
C	0.000000905	0.000000256	-0.000000373	
C	0.000000422	-0.000000271	0.000000277	
N	0.000000519	0.000000043	0.000000166	
C	0.000000302	0.000000114	-0.000000213	
C	-0.000000052	0.000000073	-0.000000950	
C	0.000000380	-0.000000306	0.000000274	
C	0.000000601	-0.000000227	0.000000226	
C	0.000000253	-0.000000719	-0.000000083	
C	-0.000000127	-0.000000878	0.000000276	
C	0.000000062	0.000000427	0.000000548	
C	-0.000000367	0.000000287	-0.000000604	
C	-0.000001473	-0.000000079	0.000002956	
C	-0.000000750	-0.000000900	-0.000000233	
O	-0.000001390	0.000000748	-0.000001707	
C	0.000000471	0.000000255	0.000000023	
C	0.000005839	0.000001461	0.000000279	
O	-0.000001328	0.000001128	-0.000003543	
H	0.000000011	-0.000001715	-0.000000551	
H	-0.000000145	0.000000244	-0.000000055	
H	-0.000000017	0.000000502	0.000000084	
H	0.000000632	0.000000074	0.000000167	
H	0.000000194	-0.000000534	-0.000000034	
H	0.000000846	-0.000000511	0.000000449	
H	0.000000746	-0.000000286	0.000000315	
H	0.000000553	0.000000145	0.000000041	
H	0.000000139	0.000000240	0.000000230	
H	0.000000499	0.000000493	0.000000301	
H	0.000000610	0.000000207	0.000000230	
H	-0.000000136	-0.000000334	-0.000000017	
H	-0.000000063	-0.000000740	-0.000000057	
H	0.000000227	-0.000001251	-0.000000754	
C	0.000000268	0.000001948	0.000001323	
H	-0.000000780	0.000000092	-0.000000059	
H	-0.000000440	-0.000000309	-0.000000570	
H	0.000000347	0.000000695	-0.000000475	
C	-0.000000906	0.000000404	0.000000339	
C	-0.000000046	0.000000837	0.000003976	
C	-0.000000012	-0.000002861	0.000001993	
H	-0.000000309	0.000000680	0.000000015	
H	-0.000000124	0.000000538	-0.000000258	
H	-0.000001609	0.000000162	-0.000000314	
H	-0.000000031	-0.000000040	-0.000000986	
H	-0.000000058	0.000000132	0.000000710	
H	0.000000262	0.000002057	0.000003385	
H	0.000000018	0.000001169	-0.000000593	
H	0.000000294	-0.000001096	0.000001216	
H	0.000000357	0.000000341	0.000000850	

Geom.No

CO_2 =-188.580938479 Ha

Atom	X	Y	Z
C	-0.000683228	-0.000000000	-0.000001192
O	0.000341891	0.000000000	-0.000158006
O	0.000341337	0.000000000	0.000159199

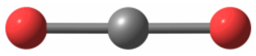

Geom.No
H_2O=-76.4197366208 Ha

Atom	X	Y	Z
O	-0.000092754	0.000000000	-0.000065587
H	0.000031598	0.000000000	0.000053695
H	0.000061157	-0.000000000	0.000011892

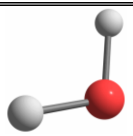

Geom.No
OH^- =-75.7262923857 Ha

Atom	X	Y	Z
O	0.000000000	0.000000000	0.000008875
H	0.000000000	0.000000000	-0.000008875

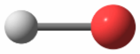

Geom.No
OOH^-.

Atom	X	Y	Z
O	0.000158315	0.000000000	-0.000030230
O	-0.000018291	-0.000000000	0.000013673
H	-0.000140024	-0.000000000	0.000016557

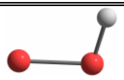

Supplement: Supplementary file 1 [file ijms-21-04417-s001.pdf]
